# Radiolabeled Antimicrobials for Infection Imaging: A Scoping Review

**DOI:** 10.3390/ijms27125313

**Published:** 2026-06-11

**Authors:** Sichen Liu, James Townley, Chuen-Yen Lau

**Affiliations:** 1 Laboratory of Clinical Immunology and Microbiology, National Institute of Allergy and Infectious Diseases, National Institutes of Health, Bethesda, MD 20892, USA; 2 Center for Infectious Disease Imaging, Radiology and Imaging Sciences, Clinical Center, National Institutes of Health, Bethesda, MD 20892, USA; 3 Alice L Walton School of Medicine, Bentonville, AR 72712, USA; james.townley@alwmed.org; 4 HIV Dynamics and Replication Program, National Cancer Institute, National Institutes of Health, Bethesda, MD 20892, USA; lauc@mail.nih.gov

**Keywords:** PET imaging, infection imaging, *in vivo* PET imaging, radiolabeled antimicrobials, infectious disease diagnostics

## Abstract

Imaging of infections has the potential to improve clinical outcomes, but pathogen-specific imaging strategies are currently unavailable. Given their target specificity, antimicrobials may be useful as molecular imaging ligands to target infections. Despite substantial development efforts, no antimicrobial-based ligands are approved for clinical use. This scoping review comprehensively surveys radiolabeled antimicrobials across antibacterial, antimycobacterial, antiviral, and antifungal drug classes, examining their progression through the translational pipeline. The review utilized PubMed and Google Scholar databases (1970–2025), following PRISMA Extension for Scoping Reviews (PRISMA-ScR) guidelines. Two reviewers independently screened titles, abstracts, and full-text articles; data were extracted, and content duplicates were removed. In total, 143 preclinical and 25 clinical articles met the selection criteria. In clinical studies, most tracers showed suboptimal specificity for infections, while some proved useful for pharmacokinetic characterization. Among preclinical studies, radiolabeled plazomicin and echinocandins (caspofungin and anidulafungin) exhibited the greatest number of preferred characteristics. In conclusion, ideal antimicrobial pharmacologic properties can be counterproductive for imaging, where rapid background clearance and a high target-to-non-target ratio (T/NT) are essential. Many radioligands demonstrate good tissue penetration but suboptimal washout, limiting their diagnostic value. *In vivo* pharmacokinetic applications during active infections are promising, though significant challenges remain for infection imaging.

## 1. Introduction

While infectious disease diagnostics have made tremendous progress in recent years, progress in infectious disease imaging is still gaining momentum. Clinicians currently diagnose infections using a combination of techniques to complement clinical evaluations, with imaging typically focused on structural approaches such as Computed Tomography (CT) and Magnetic Resonance Imaging (MRI). Structural imaging, however, often cannot distinguish infectious from non-infectious etiologies or distinguish between different types of infections with certainty. Molecular imaging approaches, especially those utilizing nuclear medicine imaging techniques such as Single-Photon Emission Computed Tomography (SPECT) and Positron Emission Tomography (PET), can potentially enable a more specific diagnosis through the *in vivo* detection of biological processes associated with various pathogens.

### 1.1. Rationale

**Figure 1:**
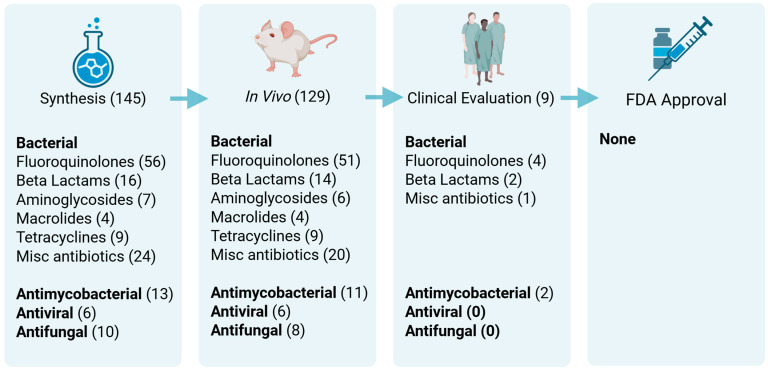
Distribution of radiolabeled antimicrobials by drug class and their progression through the translational pipeline. Of the 145 synthesized compounds—predominantly fluoroquinolones (56) and miscellaneous antibiotics (23), 129 compounds were evaluated in animal models. However, only nine compounds were tested in clinical trials, primarily focused on fluoroquinolones (4) and beta-lactams (2); tracers intended solely for pharmacokinetic studies in healthy patients were excluded from this count. No radiolabeled antimicrobial compound has yet received FDA approval. Created in BioRender. Martinez-Orengo, N. (2026) https://BioRender.com/jpsn94q, accessed on 4 June 2026.

Since antimicrobial efficacy is predicated on a drug’s ability to target specific microbial components, radiolabeled antimicrobials have been pursued as potential specific tracers for the detection of infection. As summarized in Figure 1, more than 100 radiolabeled antimicrobial-based tracers have been synthesized to date; among these, only nine have progressed to clinical evaluations, and none has received the US Food and Drug Administration (FDA) approval. Despite substantial efforts, no antimicrobial-based imaging ligands are currently approved for clinical use.

### 1.2. Objectives

Several reviews have previously examined radiolabeled antimicrobials in the context of infection imaging [1,2]. Ordonez and Jain described pathogen-specific bacterial imaging strategies, including radiolabeled antibiotics, focusing their coverage on fluoroquinolones, beta-lactams, and trimethoprim [3]. Northrup et al. provided an excellent discussion on bacterial imaging ligands in relation to extracellular and intracellular targets [4]. Welling et al. added perspectives on multimodal bacterial imaging with optical and radiological strategies [5]. We sought to build on these reviews with a comprehensive, class-by-class survey of radiolabeled antimicrobials across the spectrum of infections.

Our scoping review aims to summarize the existing literature on antimicrobial agents that have been radiolabeled with gamma- or positron-emitting isotopes for SPECT or PET imaging of bacterial, viral and fungal infections. The mechanisms of localization, stages of preclinical or clinical evaluations, key knowledge gaps and challenges will be explored. Where data are available, we assessed the desirable radiotracer properties, such as *in vivo* stability, pathogen-specific targeting, the ability to distinguish infection from sterile inflammation, adequate tissue penetration, rapid clearance from non-target tissues, and conjugation with practical isotopes, such as technetium-99m and fluorine-18. We additionally provide our perspective on emerging opportunities and priority areas for advancing the development of infection-specific radiotracers.

## 2. Methods

### 2.1. Drug and Radioisotope Selection

Antimicrobials were selected from the list of antibiotics by class using the drug classification system on Drugs.com, specifically under the category of anti-infectives. For antivirals, we limited the inclusion to widely used antivirals (see Supplemental Table S1). The following drug classes were excluded from this review: antiparasitics, amebicides, antimalarials, leprostatics, antiviral boosters, and monoclonal antibodies. The SPECT and PET radioisotope lists were derived from Karageorgou et al. and Northrup et al. [4,6].

### 2.2. Eligibility Criteria

We restricted our search to literature published in English from January 1970 through 30 June 2025, when the search was conducted. Although original full-length research articles were preferred, we also included non-redundant conference abstracts to more fully represent the research landscape. We excluded antiparasitic drugs, antimicrobial peptides, antimicrobial antibodies, review articles, books, and preprints. We only included studies utilizing radioisotopes compatible with SPECT or PET imaging, such as technetium-99m, fluorine-18, carbon-11, iodine-123, iodine-124, iodine-131, copper-64, and zirconium-89 (see Supplemental Table S2 for a complete radioisotope list).

### 2.3. Search Strategy, Information Sources and Evidence Selection

This scoping review was conducted following the PRISMA-ScR guidelines. This protocol has been uploaded onto the Open Science Framework, and it is accessible via the following reference [6]. We first compiled a comprehensive list of antimicrobial agents by class for bacterial, antimycobacterial, viral, and fungal pathogens as noted in Section 2.1 (see the Supplemental Table S1 for the complete list). We then conducted a literature search in the Google Scholar and PubMed electronic databases using the antibiotic names and the term “radiolabeled” via the Publish or Perish (PoP) software version 8.19 [7]; for example, “Radiolabeled gatifloxacin” in the keyword section of the software. This broad search strategy was deliberately chosen to avoid restricting results to specific radioisotopes, while minimizing the total number of individual searches required. Due to the exploratory nature of this scoping review and the large number of antimicrobial agents searched, we screened the first 50 results per antimicrobial agent in Google Scholar via PoP, ranked by citation/relevance. All PubMed results were also screened using the PoP software without numerical limitation, since limitation selection is not implemented in PubMed. The American spelling of “radiolabeled” was used and isotope-specific terms were not systematically searched. To supplement our database searches, we reviewed the reference lists of two comprehensive reviews on radiolabeled antimicrobials to identify additional relevant citations [1,2].

### 2.4. Evidence Selection

All citations were imported into the EndNote software version 21.5, and duplicate citations were deleted using the built-in duplicate detection function [8]. Afterwards, the screening process proceeded in two stages:

Stage 1—Title and Abstract Screening: Two reviewers independently screened the titles and abstracts against the eligibility criteria. Studies were excluded if they: (1) were review articles, books, or preprints; (2) utilized non-SPECT/PET compatible radioisotopes; (3) investigated antimicrobial peptides or antibodies, not developed small-molecule antimicrobials; (4) did not involve the radiolabeling of antimicrobial compounds; or (5) were written in a language other than English, or their abstract/article could not be obtained.

Citations identified from the radiolabeled antimicrobial review articles were merged and underwent the same screening process before being integrated with the database-based citation library [1,2]. Then another duplication check was performed on the merged library.

Stage 2—Full-Text Screening: The first two authors independently assessed the full-text articles for final inclusion. Preclinical studies were evaluated for information on chemical synthesis, radiochemistry, and animal models. Clinical studies, including randomized and non-randomized controlled trials, as well as observational studies, were evaluated for the use of radiolabeled antimicrobials for infection detection or pharmacokinetic assessment.

**Figure 2:**
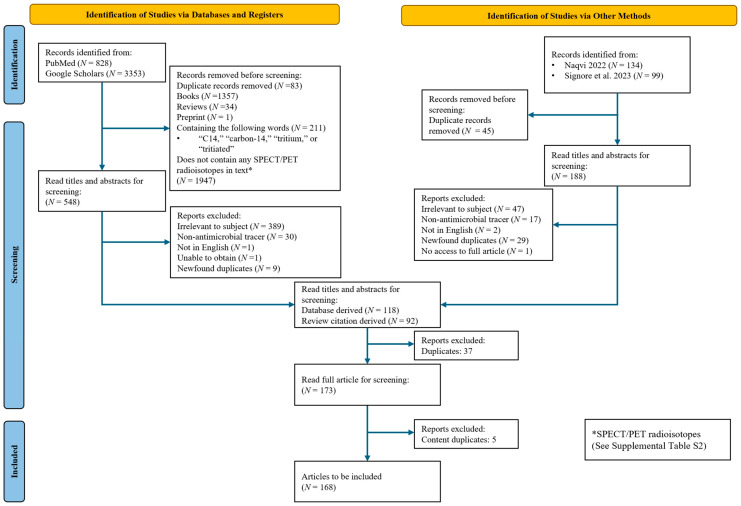
Study Selection Process Flow Diagram. * see Supplemental Table S2 for the complete list of SPECT/PET isotopes. Naqvi, 2022 [1]; Signore et al [2].

Following full-text screening, we identified “content duplicates.” Content duplicates were defined as studies that: (1) used the same base antimicrobial, linker, and radioisotope, (2) yielded the same ligand, even if via different synthesis steps, and (3) tested the compound in similar infection models without providing new pharmacokinetic, biodistribution, or clinical data. When content duplicates were identified, we retained the earliest or most comprehensive article. The study selection process is documented in a PRISMA flow diagram (Figure 2).

### 2.5. Data Charting and Results Synthesis

Tables were made for each antimicrobial class. For each radioligand derived from an antimicrobial in that class, the following data were extracted by reference as available: base antibiotic, radiolabeled ligand, peak binding, animal or human model used, type of infection, target-to-nontarget (T/NT) ratio, and additional notes of interest. The notes of interest focused on desirable tracer characteristics such as *in vivo* stability, pathogen specificity, the ability to distinguish infection from sterile inflammation, tissue penetration, clearance from non-target areas, and conjugation with practical isotopes such as technetium-99m and fluorine-18. Trends within classes were considered during the critical evaluation of future development potential.

#### T/NT Ratios by Mechanism

To characterize tracer performance, the T/NT ratios were extracted from all eligible preclinical studies and organized by antimicrobial class according to their mechanism of action. Only T/NT ratios derived from ex vivo biodistribution data were included; T/NT ratios measured via scintigraphy or calculated from Area Under the Curve (AUC) were not included because few studies measured T/NT ratios via imaging. Additionally, T/NT ratios for non-target organisms were excluded, such as T/NT measured from the antifungal-based ^99m^Tc(CO)_3_-caspofungin in *S. aureus* bacterial myositis. Where a single tracer was evaluated against multiple target pathogens, each pathogen-specific T/NT ratio was considered independently.

### 2.6. Graphics and Proofreading

Figures were created using BioRender (BioRender.com). Graphs were generated using GraphPad Prism (version 10.4.1). Claude Sonnet 4.6 was used to identify grammatical, abbreviation, and logical concerns; these concerns were addressed by authors manually to retain context. No autocorrect was used.

## 3. Results

We identified 143 preclinical and 25 clinical articles. In summary, 80 antimicrobials were labeled with SPECT or PET isotopes (e.g., carbon-11, fluorine-18, technetium-99m), including different oxidation states (e.g., technetium-99m nitride [99mTcN-] vs. technetium-99m carbonyl [^99m^T(CO)_3_-]), coordination complexes (e.g., dithiocarbamate), and linkers (e.g., 2,2′,2″,2‴-(1,4,7,10-Tetraazacyclododecane-1,4,7,10-tetrayl)tetraacetic acid or DOTA), yielding a total of 145 antimicrobial-based radioligands (Figure 1). Among all synthesized radioligands and animal studies, technetium-99m was utilized five times more frequently than fluorine-18 in synthesized radioligands and seven times more frequently in animal studies. However, fluorine-18 accounted for eight out of the 25 clinical studies conducted. For a full breakdown of radioisotopes, please see Supplemental Table S3. Most preclinical studies (86) were done in the 2010s; 23 studies have been published so far in the 2020s (Supplemental Table S4). Of these radioligands, most were evaluated *in vivo* in small-animal studies. Ultimately, 14 radioligands were administered to humans, but only nine were evaluated in patients with infections. A summary of clinical studies is presented in Table 1; the characteristics of all studies are summarized in Table 2, Table 3, Table 4, Table 5, Table 6, Table 7, Table 8, Table 9 and Table 10 by antimicrobial class. Table 1 includes ^99m^Tc-ubiquicidin_29-41_, which is a small synthetic antimicrobial peptide that binds preferentially to bacteria *in vitro* and not to activated leukocytes. It has performed well in clinical evaluations but is not yet FDA-approved in the United States for *in vivo* imaging applications [9,10]. We include it here as a benchmark comparator for antibiotic-based tracers.

As ^99m^Tc-ciprofloxacin is the radiolabeled antimicrobial that has progressed furthest on the developmental path, experience with this compound is summarized in greater depth as a case study. This is followed by a descriptive synthesis of experience with other radiolabeled antimicrobials that have undergone clinical evaluation. Findings regarding radiolabeled antibiotics that have been evaluated in preclinical and laboratory studies are subsequently described according to their level of promise. Details for each individual antibiotic are listed in the summary tables.

### 3.1. From Breakthrough to Setback: Case Study of ^99m^Tc-Ciprofloxacin

^99m^Tc-ciprofloxacin (^99m^Tc-CIP) was developed in the 1990s to specifically detect bacterial infections. As a radiolabeled form of a broad-spectrum antibiotic, it was designed to accumulate at infection sites by targeting bacterial DNA gyrase. ^99m^Tc-CIP, which was marketed as Infecton^®^, is the only tracer in this review that has undergone multiple clinical evaluations at different institutions. In a study involving 879 patients with osteomyelitis, prosthetic joint infection, and tuberculosis, Britton et al. initially found that ^99m^Tc-CIP had a sensitivity and specificity of 85.4% and 81.7%, respectively [12]. The FDA subsequently approved phase II clinical trials for Infecton. However, additional evaluations suggested a much lower specificity of 20–37.5% [14,16].

While ^99m^Tc-CIP showed early promise, including good *in vivo* stability and a relatively practical labeling, the inconsistency of results and concerns regarding its specificity curtailed further development. Even at a basic level, the broad-spectrum activity of the parent antibiotic ciprofloxacin against both Gram-positive and Gram-negative bacteria would inherently limit its ability to discriminate between bacterial species, even if it could successfully distinguish bacterial infections from other etiologies such as inflammation, malignancy or fungal infections. ^99m^Tc-CIP is also a good example of how prolonged blood retention (desirable for an antibiotic) results in a high background signal and a secondary unfavorably low T/NT. For comparison, the FDA-approved radiotracer Netspot, which targets neuroendocrine tumors, clears approximately 12-fold faster than Infecton and has a target-to-blood ratio of 80 at 1 h post-injection, whereas Infecton has a target-to-blood ratio of 3 at 12 h [37,175].

Eventually, the performance characteristics of ^99m^Tc-CIP were deemed insufficient for reliable infection diagnosis or the tracking of disease progression. The drug never achieved full FDA approval in the U.S.

Subsequently, additional ^18^F-CIP was synthesized and given to patients with confirmed bacterial infection as a proof-of-concept study where the AUC for infection loci was significant for all four patients tested [19], but no further clinical trials were conducted.

### 3.2. Clinical Evaluation of Radiolabeled Antimicrobials

A total of 14 radiolabeled antibiotics have been evaluated in humans across 25 clinical trials. However, among radiotracers other than ^99m^Tc-CIP, only three clinical trials, which separately evaluated ^99m^Tc-levofloxacin, ^99m^Tc-ceftriaxone and ^99m^Tc-ethambutol included uninfected control patients, enabling the calculation of their sensitivity and specificity [22,23,30].

Among these, ^99m^Tc-levofloxacin, another fluoroquinolone-based radioligand, demonstrated similar performance to ^99m^Tc-CIP. Its sole clinical trial reported a high sensitivity of 93.8% and a respectable specificity of 85.7% [22]. These figures are notably similar to the results from the largest clinical trial of ^99m^Tc-CIP that initially suggested its clinical utility. However, given that the fundamental issue with ^99m^Tc-CIP was not its performance in a single trial but its highly variable and often poor specificity in subsequent studies, the risk that ^99m^Tc-levofloxacin would result in a similar outcome constrained further development. Another radiolabeled fluoroquinolone that reached clinical trials is ^18^F-fleroxacin; however, it failed to specifically detect infections in patients with chronic bronchitis or complicated urinary tract infections (cUTI) [21,48]. For patients with chronic bronchitis, the lungs showed low uptake, which the investigators attributed to fibrosis. Similarly, the patients with cUTI did not show increased uptake in the kidneys or prostate [21].

The third radiotracer, ^99m^Tc-ceftriaxone (CRO), based on a beta-lactam antibiotic, exhibited inferior performance compared to both ^99m^Tc-CIP and ^99m^Tc-levofloxacin, demonstrating a sensitivity of 85.2% and a specificity of 77.8% [23]. This performance was consistent with prior preclinical studies that demonstrated good uptake in *E. coli* myositis but poor washout kinetics during imaging from organs such as the lungs over a similar timeframe. Infection visualization was poor [95,96,97]. It is possible that the decision to advance ^99m^Tc-CRO to human trials was made before the unfavorable preclinical imaging characteristics were published.

The antimycobacterial-based radioligand ^99m^Tc-ethambutol demonstrated a high sensitivity (94.9%) for the detection of extrapulmonary *M. tuberculosis* (MTB) infection in patients, though its specificity was suboptimal (83.3%) [30]. Of note, some patients were categorized as having true infection based on clinical improvement following anti-MTB therapy without supporting microbiological data. Further evaluation in more well-defined populations or structural modifications to the ligand could improve its performance. As with ^99m^Tc-CRO, preclinical models had already predicted lower specificity, yielding a T/NT of only 1.8 at 4 h post injection [160].

Finally, several other radiolabeled antibiotics, including ^99m^Tc-ceftizoxime, ^11^C-trimethoprim, and ^18^F-CIP, have been evaluated in humans [19,21,24,25]. However, these clinical trials were designed as proof-of-concept studies, enrolling only patients with confirmed infections of interest but lacking a non-infected control group. While these trials yielded promising results, their true clinical utility cannot be determined without more rigorous investigations that include appropriate control populations.

Lastly, it is worth noting that radiolabeled antibiotics not optimized for infection imaging can serve another important purpose: characterizing drug PK during active infections. For example, Tucker et al. used ^11^C-rifampin to demonstrate that the parent drug, rifampin, exhibits heterogeneous uptake among *M. tuberculosis* (MTB) brain lesions in rabbits, and subsequently confirmed similar findings in humans with MTB meningitis [27]. Through PK modeling with the radiolabeled antibiotic in rabbits, they proposed higher rifampin dosing to achieve adequate intralesional concentrations in young children with MTB meningitis [27]. Then, another study using radiolabeled rifampin revealed that blood–brain barrier (BBB) disruption during active infections plays an important role in rifampin penetration into infected regions [176]. As a result, once healing started and the BBB became less disrupted, lower drug concentrations were achieved in the lesions compared to earlier time points. This supports the need for increasing the drug dose over time to maintain a constant level of the drug the intracranial lesions. Similarly, Gordon et al. used ^11^C-rifampin to model PK in active *S. aureus* bone implant infections in preclinical studies, then correlated their findings with drug levels measured in patients and found a need to increase rifampin dosing for better drug penetration into the bone [26]. Together, these studies demonstrate how radiolabeled antibiotics can be leveraged to inform and advance pharmacological decisions in clinical practice.

### 3.3. Evaluation of Antimicrobial Based Tracers

#### 3.3.1. Radiolabeled Antimicrobials with High Translational Potential

A total of 145 radiolabeled antibiotics have been synthesized, of which 129 were tested in preclinical models (Figure 1). In this section, we focus on the ligands that showed the most promise based on preclinical model findings. We note that most radioligands undergo renal excretion and hepatic metabolism, resulting in high uptake in these organs. Therefore, when biodistribution is discussed, high uptake in the liver, kidneys, and bladder is implied unless otherwise stated.

To identify antibiotic-based radioligands with the most promising preclinical results, we evaluated studies based on the completeness of their data, prioritizing those that provided a comprehensive characterization of tracer behavior (Table 11). The likelihood of successful translation into humans was assessed based on how many of the following criteria were fulfilled: (1) favorable results on SPECT or PET imaging with a high target-to-non-target ratio; (2) T/NT close to 1 in a sterile inflammation model; (3) an appropriately low uptake in a non-target pathogen infection model, supporting tracer specificity; (4) high target signal retention measured via the percentage of injected dose per gram of tissue (%ID/g) over time (%ID/g of last time point collected − %ID/g of first time point collected)/(last time point–first time point); (5) a favorable biodistribution profile across relevant organs; and (6) favorable findings in more than one target infection model. Lastly, reports of tracer pharmacokinetic parameters were considered if available, but were not deemed essential.

Antibiotic-based tracers with the greatest potential for successful translation into humans are shown in Table 12. Of note, a clinical evaluation of ^18^F-fluoropropyl-trimethoprim (^18^F-FPTMP) was recently initiated (NCT04263792); as results are not yet available, this tracer is included in this section. The labeled antimicrobial peptide ^99m^Tc-Ubiquicidin and the fluoroquinolone ^99m^Tc-CIP are not listed because data from human studies are already available.

##### Plazomicin

Aminoglycosides are primarily used to treat serious infections caused by aerobic Gram-negative bacilli and may be used with other agents for certain Gram-positive infections. They bind to 16S ribosomal RNA within the 30S subunit to inhibit translocation. One of these, plazomicin, was labeled with technetium-99m and showed a good T/NT of 7.0 in *S. aureus* myositis. While this tracer would benefit from further validation in a clinically relevant infection model, normal muscle uptake (1.1 %ID/g) by 4 h was similar to most other organs such as the lungs (1.2 %ID/g), suggesting that muscle uptake may serve as a proxy for lung uptake in biodistribution studies. Scintigraphy showed significantly higher uptake in *S. aureus*-infected thigh compared to *C. albicans* or sterile inflammation. While the authors tested *E.coli* and *P. aeruginosa in vitro*, they did not test them *in vivo* [114].

##### Caspofungin/Anidulafungin

Two of the four approved echinocandins, which inhibit fungal β-D-glucan synthase, have been radiolabeled—caspofungin and anidulafungin. Both drugs are first-line choices for invasive candidiasis and are also efficacious against *Aspergillus* spp. *in vitro*. While biodistribution studies using ^99m^Tc(CO)_3_-caspofungin showed higher or comparable blood pool uptake relative to muscle and good T/NT ratios for both *C. albicans* (5.1) or *A. niger* (3.6) infected muscle, *in vivo* imaging showed much lower background than the biodistribution studies [171]. Further evaluation of labeled caspofungin should be considered. Similarly, ^99m^Tc(CO)_3_-anidulafungin had plasma protein binding of 77%, similar to ^99m^Tc(CO)_3_-caspofungin’s 78.7%. The biodistribution of ^99m^Tc(CO)_3_-anidulafungin demonstrated strong T/NT ratios for *C. albicans* (5.9) and *A. fumigatus* (6.3), while *S. aureus* T/NT was much lower (1.6), but *in vivo* imaging was not performed [172].

##### Trimethoprim

^18^F-FPTMP is currently being evaluated for the detection of bacterial infections in a Phase 1 clinical trial (NCT04263792), but no clinical data has been published. Trimethoprim inhibits the enzyme dihydrofolate reductase (DHFR) in bacteria to block the conversion of dihydrofolate to the active tetrahydrofolate and was labeled with fluorine-18 by Sellmyer et al. [135]. ^18^F-FPTMP successfully detected thigh infections caused by *E. coli*, but not those caused by *S. aureus.* This ligand displayed some favorable pharmacokinetic characteristics such as excellent washout by 2 h with low background seen in most organs.

In preclinical models, ^18^F-FPTMP was also able to distinguish *E. coli* infection from sterile inflammation, although some low-level uptake was observed in malignancies. Its favorable lung clearance suggests potential utility for pneumonia imaging. However, high abdominal uptake was observed on PET/CT, which the authors attributed to hepatobiliary excretion. This could limit applications involving the gastrointestinal tract [135]. While the preclinical T/NT ratio for *E. coli* infection was modest at 2.8, the ongoing Phase 1 clinical trial will be an important step in determining whether this level of target contrast is sufficient for clinically meaningful infection detection.

In a separate study, the same research group developed ^11^C-trimethoprim as a PET reporter gene imaging agent. This tracer was used to detect xenograft tumors in a murine model, which were formed from cells transfected to express *E. coli* dihydrofolate reductase. The system demonstrated high sensitivity, with ^11^C-trimethoprim being able to detect tumors comprising as few as 3 × 10^5^ cells [134]. This work highlights the potential of using this approach for non-invasively monitoring engineered cells *in vivo*.

### 3.4. Radiolabeled Antimicrobials with Moderate Translational Potential

Several additional radiolabeled antimicrobials show encouraging preliminary results but require further preclinical characterization and validation. Radiolabeled antimicrobials with moderate translational potential have shown positive results but do not fulfill one or more of the criteria for evaluating translational potential of radiolabeled antimicrobial tracers listed in Table 11.

Some radiolabeled fluoroquinolones not discussed previously, largely third- and fourth-generation agents, have demonstrated favorable imaging characteristics such as T/NT ratios well above background activity in uninfected muscle and measurable washout that reduces undesirable normal organ signal. For example, ^99m^TcN- and ^99m^TcCO_3_-labeled complexes of sitafloxacin, moxifloxacin, and garenoxacin, and ^99m^Tc-nemonoxacin retained robust signal in infected muscle while demonstrating good washout from most normal organs by 2 h post-injection [72,73,75,81,82,87]. Related compounds, including conjugates of trovafloxacin, pazufloxacin, tosufloxacin, and clinafloxacin showed slightly less washout than the tracers above but still maintained a strong infection-associated signal [62,65,77,78,79,83,84]. Future studies of these radioligands would benefit from the inclusion of lung biodistribution, the inclusion of Gram-negative infection models, and the reporting of representative scintigraphy images to better characterize contrast and biodistribution.

Other fluoroquinolones that show some promise include ^99m^Tc-danofloxacin. This is a second-generation fluoroquinolone like CIP. It achieved an encouraging T/NT ratio of 6.2 at 4 h with low lung background in a murine *S. aureus* myositis model and showed adequate washout by 4 h post-injection, but it has not been evaluated against Gram-negative pathogens [67]. In contrast, ^99m^Tc-gemifloxacin, achieved a very high T/NT ratio of 16 for *P. aeruginosa* infection, but background organ uptake was not characterized [69].

Among the beta-lactams, ^99m^Tc-ertapenem, based on the namesake carbapenem beta-lactam, demonstrated good washout by 4 h post-injection. Biodistribution data in a rat myositis model had higher uptake in *S. aureus* than *E. coli*. Conversely, visually, imaging in rabbit animal models revealed higher signal in *E. coli* infection than in *S. aureus* as no quantification was given [106]. Both models showed good washout of the radiotracer from all organs except the intestines. Further evaluation in more clinically relevant models, such as bacterial pneumonia, would be valuable. On the other hand, radiolabeled cefepime, ^99m^Tc-cefepime, showed good uptake in *E. coli* infected muscle with low lung background, especially after 24 h. Additionally, *in vitro* studies showed poor *S. aureus* uptake of only 4% [71]. It would be valuable to determine whether this tracer exhibits Gram-negative specificity. ^99m^Tc-cefoperazone also demonstrated favorably low background organ uptake at 5 h. Limited data are available at other time points, and only *S. aureus* myositis was evaluated. This tracer may be worth exploring in more clinically relevant infection models [103].

For antivirals, 9-[(1-[^18^F]fluoro-3-hydroxy-2-propoxy) methyl]guanine (^18^F-FHPG), a ganciclovir derivative, has been evaluated for herpes simplex virus (HSV) infection imaging in rats. In an HSV encephalitis model, autoradiography suggested focal signal in infected brain regions; however, other viral infections and sterile inflammation controls were not assessed, limiting conclusions regarding specificity [161]. *In vitro*, it also demonstrated strong binding affinity toward cytomegalovirus-infected cells, a herpesvirus related to HSV [162]. A related tracer, 9-[4-[^18^F]fluoro-3-(hydroxymethyl) butyl]guanine (^18^F-FHBG) has been used successfully to image HSV thymidine kinase reporter gene expression in liver tumor models, but has not been evaluated for the direct detection of HSV infections [177].

From preclinical data, other antimycobacterials and antifungals have less potential for supporting further evaluation.

### 3.5. Radiolabeled Antimicrobials with Low Translational Potential

Antimicrobial-based ligands that have demonstrated unfavorable characteristics in preclinical evaluations are described in the Supplementary Note, organized by class. Details are included in Summary Table 2, Table 3, Table 4, Table 5, Table 6, Table 7, Table 8, Table 9 and Table 10.

### 3.6. T/NT by Mechanism of Action

**Figure 3:**
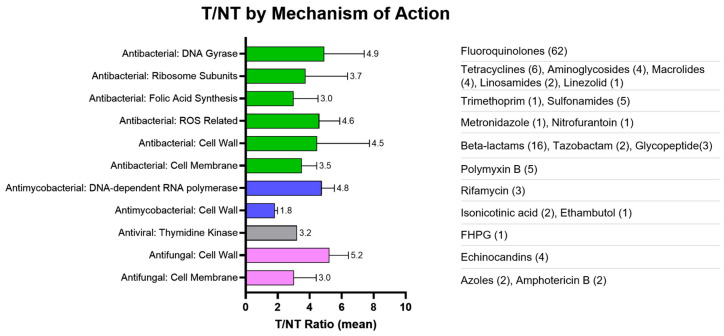
T/NT ratios by mechanism of action. The left bar graph shows mean T/NT ratios for radiolabeled antimicrobial tracers organized by mechanism of action. The number of T/NT ratios contributing to each category is indicated on the right panel. Antibacterial agents are depicted in green, antimycobacterial agents in blue, antiviral agents in gray, and antifungal agents in purple. Means are displayed. Data are graphed with Mean + SD.

We organized study results by their Mechanism of Action (MOA). Figure 3 shows a total of one hundred and thirty-eight T/NT ratios that were identified across preclinical studies within the following mechanistic categories: bacterial DNA gyrase inhibitors, comprising fluoroquinolones (62); bacterial ribosome subunit inhibitors, comprising tetracyclines (6), aminoglycosides (5), macrolides (4), lincosamides (2), and linezolid (1); bacterial folic acid synthesis inhibitors, comprising trimethoprim (1) and sulfonamides (5); bacterial reactive oxygen species (ROS) generators, comprising metronidazole (1) and nitrofurantoin (1); bacterial cell wall synthesis inhibitors, comprising beta-lactams (18), glycopeptides (4), and tazobactam (2); bacterial cell membrane disruptors, comprising polymyxin B (5); mycobacterial DNA-dependent RNA polymerase inhibitors, comprising rifamycins (3); mycobacterial cell wall synthesis inhibitors, comprising isonicotinic acid derivatives (2) and ethambutol (1); viral thymidine kinase inhibitors (1); fungal cell wall synthesis inhibitors, comprising echinocandins (7); and fungal cell membrane disruptors, comprising azoles (5) and amphotericin B (2). Ten T/NT ratios were excluded: three from bacterial cell wall synthesis inhibitors and one from bacterial ribosomal subunit inhibitors due to use of a non-target pathogen model; three from antifungal cell membrane disruptors due to the T/NT being calculated from AUC; two and one from fungal cell wall synthesis inhibitors as they were calculated from scintigraphy images and the use of a non-target pathogen model, respectively. Though this comparison reveals general trends between mechanisms, T/NT collection times were not uniform between studies.

Among antibacterials, DNA gyrase inhibitors, ROS generators, and cell wall inhibitors performed similarly with mean T/NT of 4.9, 4.6 and 4.5, respectively. Ribosome inhibitors (3.7), cell membrane disruptors (3.5) and folic acid synthesis inhibitors (3.0) followed. Between the two antimycobacterial MOAs, DNA-dependent-RNA polymerase inhibitors exhibited much higher mean (4.8) T/NT than cell wall inhibitors (1.8). Amongst antifungals, cell wall inhibitors showed a mean T/NT ratio of 5.2 and cell membrane inhibitors 3.0. Most of these T/NT ratios were calculated from ex vivo biodistribution data; actual imaging data might vary.

## 4. Discussion

**Figure 4:**
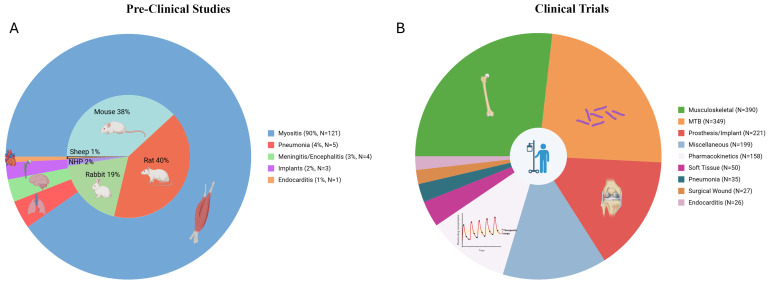
Preclinical studies (**A**) are represented in a nested pie chart. The inner ring shows animal species used, with size representing the number of preclinical studies identified; the outer ring indicates the infection model used. Among animals used, murine models predominated (mouse: 38%; rat: 41%), followed by rabbit models (19%), then NHP (2%) and sheep (1%). For infection site, 90% of preclinical studies utilized a myositis model, followed by pneumonia (4%), meningitis (3%), implants (2%) and endocarditis (1%). Clinical studies (**B**) are shown similarly with the markedly smaller inner ring reflecting the limited availability of clinical studies compared with preclinical studies. The outer ring summarizes the infection types imaged in patients across all identified clinical trials, where musculoskeletal (*N* = 390) and MTB (*N* = 349) infections were most common. This is followed by prosthetic infection (*N* = 221) and pharmacokinetic studies (*N* = 158). Less frequent infections were soft tissue infections (*N* = 50), pneumonia (*N* = 35), surgical wound infections (*N* = 27), and endocarditis (*N* = 26). Infections not clearly stated by studies or not belonging to other listed categories are categorized in Miscellaneous (*N* = 199). Not included in the graph were control patients (*N* = 41). NHP: Non-Human Primate. MTB: tuberculosis. Created in BioRender. Martinez-Orengo, N. (2026) https://BioRender.com/jpsn94q, accessed on 4 June 2026.

Infectious disease imaging is a rapidly developing field with the potential to impact the diagnosis, clinical management and research related to diverse infections. While radiolabeled antimicrobials offer some promise for advancing nuclear medicine applications, their role in infectious disease imaging remains uncertain despite the plethora of radiolabeled antimicrobial tracers already synthesized. Our review of progress to date revealed several deficiencies that continue to impede the translation of antimicrobial-based radiopharmaceuticals into clinical use. These include the inherent conflict between the ideal properties of an antimicrobial, and those of an effective radiotracer [178], the need for evaluation in relevant infection models (Figure 4), the incorporation of sterile inflammation controls, and the lack of *in vivo* imaging in preclinical studies.

Radiolabeled antimicrobials have generally performed sub-optimally as probes for *in vivo* infection detection. Antimicrobials are designed for broad tissue penetration and long half-lives to maintain therapeutic concentrations. In contrast, an ideal imaging agent requires rapid clearance of the unbound tracer from the blood and non-target tissues to minimize the background signal and achieve a high target-to-background contrast. Tetracyclines offer a clear example of this challenge. While their excellent penetration into the intracellular space is therapeutically beneficial for intracellular pathogens, their radiolabeled versions have shown a very high, non-specific background signal on SPECT scintigraphy, rendering them unsuitable for imaging. Furthermore, the broad-spectrum activity of most antibiotics, while therapeutically advantageous, reduces the target specificity desired for a pathogen-specific imaging probe.

Despite these inherent differences, antimicrobials may provide a convenient starting point for the rational development of radiopharmaceuticals but will likely require modification to optimize their utility. Modifications that could potentially improve performance include optimizing pharmacokinetics while considering alternate radionuclides and labeling strategies, and introducing structural modifications to increase pathogen specificity. For instance, reductions in molecular weight and plasma protein binding could improve renal clearance, potentially improving tracer washout [179]. Radionuclide selection could also include matching the isotope half-life to the tracer half-life while ensuring that conjugation strategies do not interfere with specific binding. Since considering these modifications can be complex and lead to unpredictable outcomes, computational modeling may facilitate the rational selection of desirable modifications or combinations of them [180]. The consideration of PK results could also be informative. Unfortunately, many studies did not explicitly study PK parameters, precluding an assessment of their utility in predicting tracer performance. The integration of PK assessments could improve future studies.

Myositis, one of the most popular infection models, offers several practical advantages, including high survival rates, accessible anatomy, and relatively low confounding from the background signal. As such, it provides a useful initial step for assessing *in vivo* tracer performance. However, myositis does not fully capture the clinical complexity of many infections. One concern with myositis models is that low background muscle signals suggest lower baseline penetration, which could result in misleadingly high T/NT ratios. This may underlie findings of low thigh muscle activity with simultaneously elevated background lung and abdomen activity. Further evaluation of ligands in more clinically relevant models, such as pneumonia, implant-associated infections, or meningitis, would strengthen translational relevance and inform further development. Pneumonia models are of particular interest as many bacterial, viral, and fungal infections occur in the lungs. Notably, radiolabeled antimycobacterial studies have already incorporated clinically relevant tuberculosis pneumonia and meningitis models, providing a precedent worth embracing [176]. Additional evaluation in clinically representative models after proof of concept in myositis models will be valuable.

Inclusion of sterile inflammation controls is also critical. Such controls are lacking in approximately 15% of studies, thus complicating the interpretation of results. Comparison between active infection models and sterile inflammation controls is needed to effectively assess performance and identify probes for further development. Lastly, a heavy reliance on ex vivo biodistribution data instead of *in vivo* imaging hinders the critical assessment of translational potential. This is because ex vivo data can be misleading and frequently differ from imaging outcomes. For example, ^99m^Tc(CO)_3_-caspofungin biodistribution results and scintigraphy T/NT ratios for *C. albicans* and *A. niger* diverge, with *A. niger* showing superior performance when assessed by scintigraphy. This discordance can also be seen in other tracers, including ^99m^Tc-CRO and ^99m^Tc-ertapenem [96,106]. Additionally, while ex vivo organ biodistribution is helpful to gauge background signal, it may not reflect *in vivo* performance. For example, ^99m^Tc-rifabutin showed high lung background only seen on scintigraphy [157]. Such discrepancies may be compounded by methodological variation, such as performing ex vivo biodistribution in murine models while conducting scintigraphy in a single rabbit model (*N* = 1) without ex vivo biodistribution (see Table 2, Table 3, Table 4, Table 5, Table 6, Table 7, Table 8, Table 9 and Table 10 for examples). Because imaging outcomes ultimately determine clinical utility, future studies should incorporate robust *in vivo* imaging assessments.

### 4.1. Translational Gap

While delineation of optimal radiotracer characteristics may seem straightforward, the development of probes is fraught with challenges, including the identification of promising ligands, infrastructure requirements, a high risk of failure even at later phases of development, and development costs. These challenges are reflected by the translational gap highlighted by our review—of 129 antibiotic-based ligands evaluated in preclinical studies, only nine have progressed to clinical trials and none are currently FDA-approved. This translational gap may at first seem discouraging, but it demonstrates efficiency in the early elimination of tracers unlikely to have clinical utility. As with any drug, the pipeline should be expected to narrow with advancing phases of development.

### 4.2. Predictive Framework

**Figure 5:**
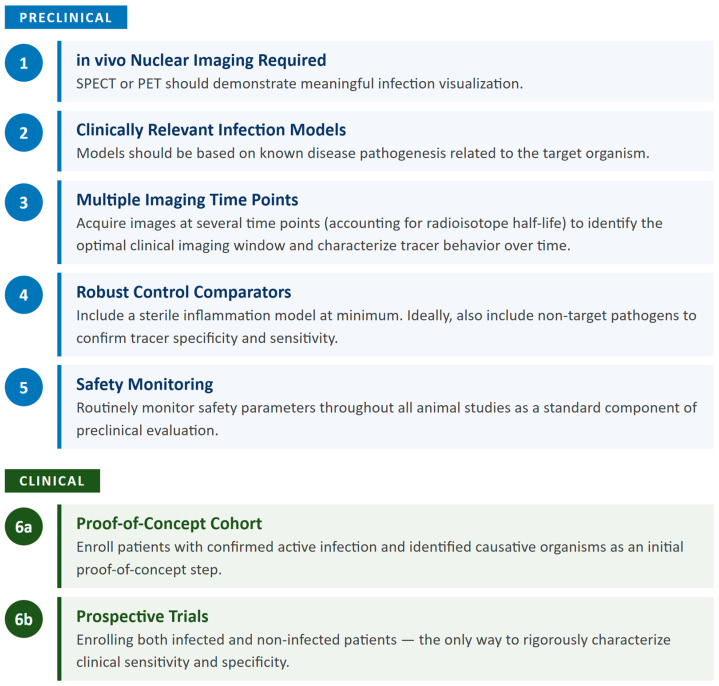
Recommended framework for future preclinical and clinical studies.

Future development of antimicrobial-based radiopharmaceuticals should adhere to the basic principles of trial design. Some suggestions to facilitate the design of relevant studies are delineated in the framework below (Figure 5).

### 4.3. Potential Clinical Impact

Accurate imaging of infectious diseases will facilitate improved patient outcomes. As a diagnostic and monitoring tool, infectious disease imaging can guide patient management, support earlier targeted treatment, and reduce unnecessary treatments, thereby minimizing treatment complications. Antibiotic stewardship could also be improved with more accurate and timely diagnoses, minimizing the development of resistance that can impair antimicrobial efficacy. In immunocompromised populations, such as those with neutropenic fever, imaging in conjunction with other diagnostic tests might distinguish among bacterial, viral and fungal etiologies or might point to a specific pathogen. Clinicians could then transition from broad-spectrum empiric antimicrobial therapies to targeted therapy.

Research applications could also support the optimization of clinical care via the characterization of infections, epidemiologic investigations, and the elucidation of pathogenesis. Pharmacokinetic data would be useful to understand tissue penetration and anatomic compartmentalization of the parent antimicrobial. Drug penetration studies could be done not only to understand biodistribution, but also to understand the impact of concomitant inflammation and the stage of infection [181]. For example, increases in BBB permeability during bacterial meningitis facilitate drug penetration; with the improvement of the infection, BBB permeability decreases. For beta-lactams, this dynamic permeability modulates drug concentrations in the central nervous system (CNS) depending on meningitis severity [182]. In an MTB meningitis model, PET imaging with ^11^C-rifampin was able to predict the need for a higher dose exceeding 30mg/kg to achieve adequate intralesional concentrations in patients [27,176]. Infectious disease imaging can support precision medicine approaches by enabling optimized dosing for specific pathogens within specific tissue compartments.

Immediate clinical impact will largely depend upon access to nuclear medicine technologies, which are disproportionately available in high-income settings. In settings where the technology is available, radiation exposure may be a concern. Appropriate safety measures will be necessary for both patients and staff. While these technologies may not be available to all, the knowledge gained from radiolabeled antimicrobial studies will be globally available. For example, pharmacokinetic insights can inform the management of infectious diseases in resource-limited settings, where the burden of infections is disproportionately high. While ethical concerns about unequal benefits may arise, all settings will benefit from the optimization of evidence-based care.

### 4.4. Limitations

Our assessment and recommendations are based on data from available reports. Unreported and/or ongoing studies may have addressed some of the questions raised in this review. Nonetheless, the general principles of radioligand innovation still apply. While antibiotic-based tracers currently have limited applications for imaging infections, other tracers, such as radiolabeled ^99m^Tc-ubiquicidin have successfully targeted bacteria-specific pathways [10]. For fungal infections, ^18^F-fluorocellobiose has emerged as a particularly promising tracer for the detection of invasive *Aspergillus fumigatus*, leveraging the unique carbohydrate metabolism of molds [183]. While antibiotic-based tracers face inherent challenges, the broader field of infection-targeted molecular imaging continues to advance through the exploration of alternative microbial mechanisms.

Because this is a scoping rather than a systematic review, we did not formally appraise study quality or perform a statistical analysis. Furthermore, the comparison of studies was challenging because the results required for our framework assessment of human translational potential were often incomplete, with missing lung biodistribution data, absent sterile inflammation controls, and inconsistent T/NT measurement time points. While our methodology was designed to maximize the retrieval of relevant literature, our strategy of searching “Radiolabeled + [antibiotic name]” and the restriction of Google Scholar searches to the first 50 results per antimicrobial agent may have resulted in the exclusion of some relevant studies, particularly those that are less frequently cited or more recently published and therefore ranked lower by Google Scholar’s algorithm for sorting by relevance. Additionally, we used the American spelling “radiolabeled” as the primary search term. Alternative spellings (e.g., “radiolabelled”) and isotope-specific terms (e.g., 99mTcCiprofloxacin) were not systematically searched, which may have resulted in the exclusion of some relevant studies, particularly from the non-US literature. Publication bias likely limited our discovery of negative or inconclusive studies, and the inclusion of conference abstracts increased the variability in data quality. Lastly, while we covered literature across several decades (Supplemental Table S4), evolving technology complicates the comparison of studies from different time periods.

## 5. Conclusions and Future Directions

Continued advances in infectious disease imaging depend on the development of radiopharmaceuticals with clinical and research utility. Current literature suggests that radiolabeled plazomicin, caspofungin and anidulafungin are among the antibiotic-based ligands potentially meriting further investigation. Several additional tracers also show potential. Modifications to optimize performance may be informed by modeling studies [184]. Findings from myositis models, a frequent choice for proof-of-concept studies, should be built upon with evaluations in more clinically representative animal models with robust *in vivo* imaging assessments prior to clinical trials. Lastly, studies evaluating the pharmacokinetics of radiolabeled antimicrobials in animal infection models have been particularly insightful and merit further development as they have the potential to impact patient care [26,27,154].

## Figures and Tables

**Table 1:** Clinical evaluations of radiolabeled antimicrobials.

Ligand	Study Size	Infection Type	Sensitivity (%)	Specificity (%)	Microbiological Data	Notes	Ref.
^99m^Tc-Ubiquicidin _29-41_	*N* = 622	Wide range, predominantly musculoskeletal infection	95.5	92.5	-	Sensitivity and specificity were calculated from pooled values from 15 clinical studies.	[9]
^99m^Tc-CIP	*N* = 56	Wide range including deep seated infections	84 (4 h)	96 (4 h)	Yes	An early study suggested ^99m^Tc-CIP performed better than tagged white blood cells. Skeletal infection subgroup performed the worst in that study.	[11]
	*N* = 879	Wide range including deep seated infections	85.4 (4 h)	81.7 (4 h)	Yes (partial)	Multinational study evaluating multiple infection types including osteomyelitis, prosthesis, endocarditis, MTB, surgical infections. Appropriate diagnostic inclusion criteria. Specificity highest for surgical wound infections and endocarditis. Orthopedic prosthesis infection showed best performance—sensitivity 96% and specificity 92%	[12]
	*N* = 71	OM/SA	84.2 (4 h)	54.5 (4 h)	Yes (partial)	Non-infectious inflammatory osteo-articular patient as control. Some patients exhibited thyroid uptake. Pediatric patient bone growth plate had high uptake, obscuring infection site. SA subgroup had higher sensitivity than OM subgroup. Hip and distal hand joints performed the worst.	[13]
	*N* = 16	Prosthesis joint infection	86 (4 h)	20 (4 h)	Yes (partial)	At 24 h, sensitivity was 80% and specificity was 78%. One patient with false negative imaging had antibiotic treatment prior to scan.	[14]
	*N* = 21	Pulmonary tuberculosis	80.0 (3 h)	90.9 (3 h)	Yes	Healthy or latent MTB patients as control group. Of active MTB patients, 50% of cases with post treatment scan had SPECT resolution, while other half had no change in uptake.	[15]
	*N* = 27	OM/SA knee	100	37.5	Yes (partial)	T/NT ratios given for all time points with no significant changes in between 4 and 24 h. Very detailed recording of patient parameters.	[16]
	*N* = 22	Prosthesis joint infection/OM/SA	85 (4 h)	92 (4 h)	Yes (partial)	Predominant prosthesis infections (n = 20). No significant changes between interpretation on SPECT imaging between 1 h and 4 h imaging. No final diagnosis given for non-infected patients for added context.	[17]
	*N* = 45	OM	97.2 (4 h)	80.0 (4 h)	Yes	*S. aureus* and *P. aeruginosa* isolates predominate. False positive seen with high bone growth and bone tumor presence.	[18]
^18^F-CIP	*N* = 4	Bacterial soft tissue infection	-	-	Yes	Patients are selected with known infections as proof of concept. Unknown underlying diagnosis of patients (chronic vs. acute infection). AUC between infected vs. not infected was significant across all patients.	[19]
	*N* = 12	PK in healthy volunteers	-	-	-	PK study equating PET imaging signal to PK parameters.	[20]
^18^F-fleroxacin	*N* = 10	Bronchitis and UTI	-	-	Yes	Did not work for either infection. Patients with bronchitis showed decreased drug accumulation, thought to be secondary to fibrosis.	[21]
^99m^Tc-levofloxacin	*N* = 30	Musculoskeletal infection	93.8 (4 h)	85.7 (4 h)	Yes	T/NT in infected patients hovered around 2.5 by 4 h. Individual patient diagnoses unclear. Culture isolates were only *S. aureus* or *E. coli*.	[22]
^99m^Tc-ceftriaxone	*N* = 36	Orthopedic infection	85.2 (1 h)	77.8 (1 h)	Yes	Individual patient diagnoses unclear. Culture isolates not shown.	[23]
^99m^Tc-ceftizoxime	*N* = 5	Diabetic foot OM	-	-	No	Confirmed patients with osteomyelitis diagnosis as proof of concept. True positives and negatives inappropriately based on ^99m^Tc-Methylene Diphosphonate scan instead of histopathology or culture.	[24]
^11^C-trimethoprim	*N* = 3	Pneumonia/OM	-	-	Yes	Radiotracer not taken up by tumors controls. Bone marrow had higher uptake and did not clear like other organs. Further clinical trial results pending.	[25]
^11^C-rifampin	*N* = 3	OM (PK)	-	-	Yes	Confirmed *S. aureus* OM patients. Pharmacokinetic study to model bone penetration showed no difference between infected vs. noninfected bone uptake. However, pulmonary MTB patient data showed increased rifampin exposure when dose increased from 35 to 45 mg/kg in OM treatment.	[26]
	*N* = 10	Tuberculosis Meningitis (PK)	-	-	Yes	Confirmed MTB with microbiological diagnosis. PK study in humans with MTB meningitis that corroborates ^11^C-rifampin PK in rabbit MTB meningitis model.	[27]
^99m^Tc-ethambutol	*N* = 16	Pulmonary and OM tuberculosis	-	-	Yes	Two subjects were healthy volunteers. No quantitative measurements. No accompanying CT scan to correlate with SPECT images.	[28]
	*N* = 19	Tuberculosis lymphadenitis	-	-	Yes	No control group. All had confirmed MTB lymphadenitis; 42% with organ involvement. Cervical lymphadenitis detected at highest rate (63.6%). Mediastinal adenopathy detected at 28.5% and no abdominal involvement detected. Poor overall performance.	[29]
	*N* = 168	Pulmonary and extra-pulmonary tuberculosis	94.9 (4 h)	83.3 (4 h)	Yes	Extra-pulmonary MTB patients with diverse infection locations. SPECT had 92.9% concordance with histopathological data. Pulmonary MTB subgroup had slightly better specificity than extra-pulmonary MTB patients.	[30]
^18^F-lemofloxacin	*N* = 2	PK in healthy volunteers	-	-	-	Labeled for pharmacokinetic study. Lung uptake was half of liver uptake.	[31]
^18^F-trovafloxacin	*N* = 16	PK in healthy volunteers	-	-	-	Biodistribution and pharmacokinetics only. AUC of lung was third highest. Penetration in CNS exceeds MIC_90_ of many non-resistant pathogens.	[32]
^18^F-pretomanid	*N* = 6	PK in healthy volunteers	-	-	-	Brain parenchyma penetration was almost 2× plasma concentration.	[33]
^18^F-FHBG	*N* = 10	PK in healthy volunteers	-	-	-	High liver and kidney/bladder signal due to metabolism. Mild intestinal accumulation over time.	[34]
^18^F-fluconazole	*N* = 9	PK in healthy volunteers	-	-	-	Uniform brain penetration. Prostate and bowel exhibited higher penetration than the rest of the organs.	[35]

^99m^Tc: technetium-99m; ^18^F: fluorine-18; ^11^C: carbon-11; CIP: ciprofloxacin; OM: osteomyelitis; SA: septic arthritis; UTI: urinary tract infection; MTB: *M. tuberculosis*; FHBG: 9-(4-^18^F-fluoro-3-[hydroxymethyl]butyl)guanine; (PK): indicating PK study to elucidate drug behavior in patients with infections, not in healthy patients; MIC_90_: Minimum Inhibitory Concentration required to inhibit 90% of bacteria.

**Table 2:** Radiolabeled fluoroquinolones.

Antibiotic	Ligand	Peak *In Vitro* Binding	Model	Infection Model	T/NT	Notes	Ref.
Ciprofloxacin (CIP)	^99m^Tc-CIP	*S. aureus*: 58.5%, *P. aeruginosa*: 50.2%, *E. coli*: 43.9%	-	-	-	This was published as a Supplemental—no preclinical models done.	[36]
		-	Mouse	*S. aureus* myositis	4.2 at 12 h	Sterile inflammation T/NT at 1.2 by 6 and 12 h. Blood pool uptake was 2× as normal muscle by 12 h. Lung has high uptake.	[37]
			Rabbit	*S. aureus* implant infection	1.8 at 24 h	Highest T/NT ratio at 19 days post-surgery/infection. However, no significant difference between implanted knee vs. normal knee.	[38]
			Human	-	-	See Table 1.	
	^99m^Tc(CO)_3_-CIP	*S. aureus*: 3.62–4.23%	Rat	*S. aureus* myositis	3.3 at 8 h	Sterile inflammation T/NT 1.4 at 8 h. Infected muscle uptake lower than blood pool and other organs. Scintigraphy showed high background signal.	[39]
	^99m^Tc(V)O-CIP	-			2.1 at 8 h	Sterile inflammation T/NT 2.9 at 4 h. Infected muscle uptake lower than blood pool and other organs. Scintigraphy showed high background signal.	
	^68^Ga-CIP-PA-SCN-Bz-DOTA	*S. aureus*: 0.9–1.0%	Rat	*S. aureus* myositis	3.0 at 2 h	Sterile inflammation T/NT 1.5. High blood pool residual. Higher organ background than for NOTA sister compound. Blood pool uptake higher than non-muscular organs (stomach, heart).	[40]
	^68^Ga-CIP-PA-SCN-Bz-NOTA	*S. aureus*: 1.6–2.3%	Rat	*S. aureus* myositis	6.7 at 2 h	Sterile inflammation T/NT 2.2. High blood pool residual. Blood pool uptake higher than non-muscular organs (stomach, heart).	
	^68^Ga-DOTA-CIP	*S. aureus*: 1.1%, *P. aeruginosa*: 1.3%	-	-	-	Poor *in vitro* binding with *S. aureus* at 1.1% and 1.3% for *P. aeruginosa*. Not used in *in vivo* studies.	[41]
	^18^F-CIP	-	-	-	-	MIC against *S. aureus*, *E. faecalis*, *E. coli*, *P. aeruginosa* did not differ between ^18^F-CIP and CIP.	[42]
		-	Human	-	-	See Table 1.	
	^18^F-COPCA	-	-	-	-	No binding *in vitro* to *S. aureus*	[43]
	^11^C-methyl-CIP	-	-	-	-	No further *in vitro* or *in vivo* studies done	[44]
	^18^F-alkylate-CIP	-	-	-	-	No further *in vitro* or *in vivo* studies done	[45]
	^99m^TcN-CP-F2XT	-	Mouse	*S. aureus* myositis	4.8 at 4 h	Sterile inflammation T/NT 2.51. Tested organs such as lungs had higher uptake than infected muscle	[46]
	^99m^TcN-CP-FXDTC	-			1.5 at 4 h	No sterile inflammation model. No other biodistribution data available. Authors stated this radioligand more lipophilic.	
	^99m^Tc(CO)_3_-CIP-DTC	-	Mouse	*S. aureus* myositis	1 to 10 at 2 h	Very high organ background signal in all six complexes tested.	[47]
Fleroxacin	^18^F-fleroxacin	*E. coli*: 70% at 12 h	Rabbit	*E. coli* myositis	-	No sterile inflammation model. T/NT not calculated. Less fleroxacin seen in infected rabbit thigh than normal muscle. Low heart and lung uptake by 2 h; high bone uptake.	[48]
		-	Human	-	-	See Table 1.	
Norfloxacin	^99m^Tc-norfloxacin	*S. aureus*: ~50% at all times	Rat	*S. aureus* myositis	6.1–6.9 at 2 h	Cannot distinguish sterile inflammation from infection.	[49]
	^99m^Tc(CO)_3_-norfloxacin	*S. aureus*: 5.58–6.64% at 1 h	Rat	*S. aureus* myositis	-	No T/NT calculated via biodistribution or scintigraphy. Scintigraphy showed high background with infection comparable to lung, and much lower than other organs.	[50]
	^99m^TcN-norfloxacin-DTC	-	Mouse	*S. aureus* myositis	3.5 at 4 h	Sterile inflammation T/NT 1.2. Very high lung background, comparable to liver, a metabolizing organ.	[51]
Difloxacin	^99m^Tc-difloxacin	*S. aureus*: ~50% at 1 h	Rat	*S. aureus* myositis	3.7 at 4 h	Sterile inflammation T/NT 3.3. Cannot distinguish sterile inflammation from infection.	[52]
Pefloxacin	^99m^Tc-pefloxacin	*S. aureus*: ~50% at 1 h	Rat	*S. aureus* myositis	4.0 at 4 h	Sterile inflammation T/NT 3. Cannot distinguish sterile inflammation from infection.	
		-	Mouse	*S. aureus* myositis	3.8 at 4 h	Sterile inflammation T/NT 1.3. Lung uptake was low but high stomach/intestinal uptake. Blood pool was 2× infected muscle.	[53]
Lomefloxacin	^99m^Tc-lemofloxacin	-	Rat	*S. aureus* myositis	6.6	No stated T/NT collection time. Blood pool uptake was ~3× higher than normal muscle.	[54]
	^99m^Tc(CO)_3_-lemofloxacin	*S. aureus*: 6.7–8.18% at 1 h.	Rat	*S. aureus* myositis	-	No T/NT done via biodistribution or scintigraphy. Scintigraphy showed high background signal with infection comparable to lungs, but much lower than other organs.	[50]
	^18^F-lemofloxacin	-	Human	-	-	See Table 1.	
Ofloxacin	^99m^Tc-ofloxacin	-	Rat	*S. aureus* myositis	4.3	No stated T/NT collection time. Blood pool uptake was ~3× higher than normal muscle.	[54]
	^99m^Tc(CO)_3_-ofloxacin	-	Mouse	*S. aureus* myositis	2.0 at 4 h	High serum protein binding at 70%. High organ background signal.	
Rufloxacin	^99m^Tc-rufloxacin	*S. aureus*: 77% at 11 h, *E. coli*: 70% at 12 h	Mouse	*E. coli* myositis	10.0 at 3 h	Sterile inflammation T/NT 3 at 3 h; 1 at 12 h. Lung uptake low. Adequate organ washout by 3 h. Blood pool still higher than most organs by 12 h. High bone uptake.	[55]
		*S. aureus*: ~72% at 2 h	Rat	*S. aureus* myositis	4.4 at 2 h	Sterile inflammation T/NT of 1. No lung biodistribution. Blood pool uptake comparable to most organs tested at 2 h.	[56]
Enrofloxacin	^99m^Tc-enrofloxacin	-	Rabbit	*Salmonella typhi* myositis	Scintigraphy: 1.6 at 2 h	Serum protein binding of 59%. T/NT measured via scintigraphy, with best visualization at 1–2 h. Limited organ biodistribution data available besides renal and hepatic parameters. T/NT at 4 h was 1.2.	[57]
		*S. aureus*: 3%; *C. albicans*: 4.5%	Rat	*S. aureus* myositis	3.8 at 22 h	Cannot distinguish sterile inflammation. Higher uptake in all organs than infected thigh. Scintigraphy confirmed finding.	[58]
Levofloxacin	^99m^Tc-levofloxacin	*S. aureus*: 75%; *E. coli*: 45%. All at 1 h	Rat	*S. aureus* and *E. coli* myositis	*S. aureus*: 11.4; *E. coli*: 3.5. All at 4 h.	Sterile inflammation T/NT 3.83 at 4 h. Lung background is relatively low, but comparable to blood pool. Parameters comparable to ^99m^Tc-CIP in same study.	[59]
		-	Rabbit	*E. coli*, *P. aeruginosa*, S. typhi myositis	*E coli*: <2; *P. aeruginosa*: 5.4; *S. typhi*: 2.7. All at 4 h.	Serum protein binding 48.9%. Sterile inflammation T/NT ratio ~2. Limited organ biodistribution data besides renal and hepatic parameters.	[60]
		-	Human	-	-	See Table 1.	
	^99m^Tc(CO)_3_-levofloxacin	*S. aureus*: 5.47–5.89% at 1 h	Rat	*S. aureus* myositis	-	No T/NT done via biodistribution or scintigraphy. Scintigraphy showed high background with infection comparable to lungs, but much lower than other organs.	[50]
Temafloxacin	^99m^Tc(CO)_3_-temafloxacin-DTC	*Streptococci pneumoniae*: ~80% at 1.5 h.	Rat	*S. pneumoniae* myositis	4.0 at 2 h.	Sterile inflammation T/NT 1.1. No lung biodistribution data available. Adequate organ washout by 2 h like ^99m^Tc-rufloxacin.	[61]
Pazufloxacin	^99m^Tc(CO)_3_-pazufloxacin-DTC	*E coli*: 75% at 1.5 h	Mouse	*E. coli* myositis	4.0 at 2 h	Sterile inflammation T/NT 1. No lung biodistribution data available. Adequate organ washout by 2 h, mildly better than ^99m^Tc-rufloxacin.	[62]
	^99m^TcN-pazufloxacin-DTC	*E coli*: 75% at 1.5 h	Rat	*E. coli* myositis	5.2 at 2 h	Sterile inflammation T/NT 1. No lung biodistribution available. Adequate organ washout by 2 h, mildly better than ^99m^Tc-rufloxacin.	[63]
Sarafloxacin	^99m^Tc-sarafloxacin	-	Mouse	*S. aureus* myositis	4.2 at 2 h	T/NT for sterile inflammation 3.25 at 2 h, cannot distinguish sterile inflammation from infection.	[64]
Tosufloxacin	^99m^Tc(CO)_3_-tosufloxacin-DTC	*Proteus mirabilis*: 80% at 1.5 h	Rat	*Proteus mirabilis* myositis	5.3 at 2 h	Model pathogen usually causes UTI; no further testing with Gram positive bacteria. Sterile inflammation T/NT 1.2, no lung biodistribution data available. Adequate organ washout by 2 h, mildly better than ^99m^Tc-rufloxacin. Liver and other organs had similar uptake except for kidneys.	[65]
Sparfloxacin	^99m^Tc-sparfloxacin	-	Rabbit	*S. aureus* myositis	4.0 at 2 h	Limited information available	[66]
Danofloxacin	^99m^Tc-danofloxacin	-	Mouse	*S. aureus* myositis	6.2 at 4 h	Sterile inflammation T/NT 2.8 at 4 h. At 4 h, infected muscle uptake was significantly lower than blood pool. However, by 24 h, good washout was observed with low background signal in the lungs and other organs, while infected muscle had good uptake retention.	[67]
Gemifloxacin	^99m^Tc-gemifloxacin	*S. pneumoniae*: 63% at 1.5 h	Rat	*S. pneumoniae* myositis	3.3 at 2 h	Limited biodistribution data reported. Sterile inflammation T/NT of 1. Serum protein binding of 55%.	[68]
			Rabbit	*Salmonella typhi*, *K. pneumonia*, *P. aeruginosa* myositis	*Salmonella typhi*: 8; *K. pneumonia*: 8.8; *P. aeruginosa*: 16.51. All at 4 h	Limited biodistribution data reported, restricted to the spleen and kidneys/bladder. Scintigraphy in mice showed high signal from liver as well, but lung background looked reasonable.	[69]
	^99m^Tc(CO)_3_-gemifloxacin		Mouse	*S. aureus* myositis	9.7 at 4 h	Relatively high lung background uptake. No sterile inflammation model. Blood pool 8x higher than infected muscle uptake at 24 h.	[70]
Gatifloxacin	^99m^Tc-gatifloxacin	*E. coli*: 50% at 1 h	Rat	*E. coli* myositis	4.5 at 3 h	Cannot distinguish sterile inflammation.	[71]
	^99m^TcN-gatifloxacin-DTC	*S. pneumoniae*: 75% at 1.5 h	Rat	*S. pneumoniae* myositis	5.0 at 2 h	Sterile inflammation performance T/NT~1. Adequate organ washout by 2 h similar to ^99m^Tc(CO)_3_-tosufloxacin-DTC. No lung biodistribution reported.	[68]
Sitafloxacin	^99m^TcN-sitafloxacin dithiocarbomate (SFDE)	*S. aureus*: 19% at 1.5 h	Rat	*S. aureus* myositis	7.4 at 2 h	Sterile inflammation T/NT at 1.1. No lung distribution data noted. Scintigraphy showed good localization of infected thigh with acceptable organ background and good washout by 2 h.	[72]
			Rabbit		-	One rabbit infected. Infection visually distinguishable, but no T/NT calculated. No thoracic region shown,	
	^99m^Tc(CO)_3_-SFDE	-	Rat	*S. aureus* myositis	5.8 at 1.5 h	Sterile inflammation T/NT at 1.2. No lung distribution data. Scintigraphy showed good localization in infected thigh with acceptable organ background with good washout by 2 h.	[73]
Moxifloxacin	^99m^Tc-moxifloxacin	-	Rat	*E. coli* myositis	6.8 at 1 h	Biodistribution showed higher blood pool value than inflamed muscle after 2 h. Relatively high lung background, no sterile inflammation tested.	[74]
		-	Rabbit	*E. coli* myositis	1.8 at 3 h	Scintigraphy shows localization in infected thigh but activity appeared lower than ex vivo biodistribution data.	
	^99m^Tc(CO)_3_-moxifloxacin dithiocarbamate (MXND)	*S. aureus*: 78% at 1.5 h	Rat	*S. aureus* myositis	4.7 at 2 h	Sterile inflammation T/NT 1.2. No lung biodistribution tested. Good organ washout by 2 h, similar to ^99m^Tc(CO)_3_-SFDE.	[75]
	^99m^TcN-MXND	*S. aureus*: 73% at 1.5 h			4.5 at 2 h	Sterile inflammation T/NT 1.2. No lung biodistribution tested. Good organ washout by 2 h, similar to ^99m^Tc(CO)_3_-SFDE.	
Trovafloxacin (TVN)	^18^F-TVN	-	Rat	*E. coli* myositis	2.0 at 2 h	No sterile inflammation model. Intestine uptake increases overtime, testicular uptakes higher in infected animals.	[76]
		-	Rabbit	*E. coli* myositis	-	Decreased ligand accumulation in infected muscle compared to normal muscle, No T/NT as Area under curve (AUC) was measured. Scintigraphy did not reveal infection well and had high abdominal uptake.	
		-	Human	-	-	See Table 1.	
	^99m^Tc-TVN	MRSA: 60% at 1.5 h	Rat	*S. aureus* myositis	4.5 at 2 h	Sterile inflammation T/NT 1.2, No lung biodistribution reported. Adequate organ washout by 2 h, mildly better than ^99m^Tc-rufloxacin.	[77]
	^99m^TcN-TVN dithiocarbamate (TVND)	MRSA: 70% at 1.5 h	Rat	MRSA myositis	5.0 at 2 h	Minimal difference compared to ^99m^Tc-TVN, no lung biodistribution reported. Adequate organ washout by 2 h, mildly better than ^99m^Tc-rufloxacin.	[78]
	^99m^Tc(CO)_3_-TVND	MRSA: 60% at 1.5 h	Rat	MRSA myositis	4.6 at 2 h	Minimal difference compared to ^99m^Tc-TVN, no lung biodistribution reported. Adequate organ washout by 2 h, mildly better than ^99m^Tc-rufloxacin.	[79]
Garenoxacin (GXN)	^99m^Tc-GXN	MRSA: 65%; *S. pneumoniae*: 67%. All at 1.5 h	Rat	MRSA and *S. pneumoniae* myositis	MRSA: 4.0; *S. pneumoniae*: 4.2 at 2 h	Sterile inflammation T/NT of 1.2. Adequate organ washout by 2 h similar to ^99m^TcN-gatifloxacin-DTC, but worse than ^99m^TcN-GXND and ^99m^Tc(CO)_3_-GXND. No lung biodistribution reported.	[80]
	^99m^TcN-GXN dithiocarbamate (GXND)	MRSA: 15%; *S. pneumoniae*: 14%. All at 1.5 h	Rat	MRSA and *S. pneumoniae* myositis	MRSA: 5.4; *S. pneumoniae*: 5.5 at 2 h	Sterile inflammation T/NT of1.2. Good organ washout by 2 h, better than ^99m^Tc-GXN, similar to ^99m^Tc(CO)_3_-SFDE. No lung biodistribution reported.	[81]
	^99m^Tc(CO)_3_-GXND	MRSA: 60%; *S. pneumoniae*: 65%. All at 1.5 h	Rat	MRSA and *S. pneumoniae* myositis	MRSA: 4.4; *S. pneumoniae*: 5.3 at 2 h	Sterile inflammation T/NT of 1.2. Good organ washout by 2 h, better than ^99m^Tc-GXN, similar to ^99m^Tc(CO)_3_-SFDE. No lung biodistribution reported.	[82]
Clinafloxacin (CNN)	^99m^Tc(CO)_3_-CNN dithiocarbamate (CNND)	MRSA: 60% at 2 h	Mouse	*S. aureus* myositis	5 at 2 h	Sterile inflammation T/NT of 1.2. Organ washout similar to ^99m^Tc-GXN. No lung biodistribution reported.	[83]
	^99m^TcN-CNND	MRSA: ~60% at 2 h	Mouse	*S. aureus* myositis	4.05 at 2 h	Sterile inflammation T/NT of 1. Organ washout similar to ^99m^Tc-GXN. No lung biodistribution reported.	[84]
	^99m^Tc-CNN	MRSA: 62% at 2 h	Rat	*S. aureus* myositis	5	Only abstract assessable.	[85]
Prulifloxacin	^99m^Tc-prulifloxacin	*S. aureus*: 40% at 2 h	Rat	*S. aureus* myositis	3.2 at 2 h	Sterile inflammation T/NT of 1. No lung biodistribution noted in study. Good organ washout by 2 h, similar to ^99m^Tc(CO)_3_-SFDE.	[86]
			Rabbit	*S. aureus* myositis	-	Adequate distinction of infection focus from background on scintigraphy. Thoracic area with notable background. High liver/gallbladder uptake not seen in rat biodistribution study.	
Nemonoxacin	^99m^Tc-nemonoxacin	MRSA: 70%, *S. pneumoniae*: ~62%. All at 2 h	Mouse	MRSA, *S. pneumoniae* myositis	MRSA: 5.2, *S. pneumoniae*: 5.4 at 2 h	Sterile inflammation T/NT at 1.1. No lung biodistribution done. Good organ washout by 2 h, similar to ^99m^Tc(CO)_3_-SFDE. Scintigraphy demonstrated clear localization with higher signal of infection site but whole mouse image displayed no signals from other organs.	[87]

^99m^Tc: technetium-99m; ^99m^TcN: technetium-99m nitrido; ^99m^Tc(CO)_3_: technetium-99m carbonyl; ^11^C: carbon-11; ^68^Ga: gallium-68; CIP: ciprofloxacin; CIP-PA: Ciprofloxacin propyl amine; DOTA: 1,4,7,10-tetraazacyclododecane-1,4,7,10-tetraacetic acid; SCN-Bz-DOTA: 2-(p-isothiocyanato)-1,4,7,10-tetraazacyclododecane-1,4,7,10-tetraacetic acid; SCN-Bz-NOTA: 2-(p-isothiocyanato)-1,4,7-triazacyclononane-1,4,7-triacetic acid; COPCA: 6-fluoro-1,4-dihydro-1-cyclopropyl-4-oxo-7-[4-[^18^F]fluoro-phenacyl-1-piperacinyl]-chinolincarboxylic acid; CPF2XT: ciprofloxacin xanthate; CPFXDTC: ciprofloxacin dithiocarbamate; DTC: dithiocarbamate; SFDE: sitafloxacin dithiocarbonate complex; MXND: moxifloxacin dithiocarbamate complex; TVN: trovafloxacin; TVND: trovafloxacin dithiocarbamate; GXN: garenoxacin; GXND: garenoxacin dithiocarbamate; CNND: clinafloxacin dithiocarbamate; MRSA: Methicillin-resistant staphylococcus aureus.

**Table 3:** Radiolabeled beta-lactams.

Antibiotic	Ligand	Peak *In Vitro* Binding	Animal Model	Infection Model	T/NT	Notes	Ref.
Benzylpenicillin	^177^Lu-benzylpenicillin	-	Rabbit	-	-	Synthesized. PK in rabbits.	[88]
Amoxicillin	^99m^Tc-amoxicillin	*S. pneumoniae*: 60% at 2 h	Rabbit	*S. pneumoniae* myositis	4.7 at 2 h	No sterile inflammation control. Higher serum protein binding of 76% compared to unlabeled compound. Scintigraphy of infected rabbit showed very high background signal at 2 h but better at 24 h.	[89]
Sultamicillin	^99m^Tc-sultamicillin	*S. aureus*: 75%, *E. coli*: 90%. All at 2 h	-	-	-	*In vitro* only.	[90]
Cefazolin	^99m^Tc-cefazolin	-	Mouse	*S. aureus* myositis	4.7 at 2 h	Sterile inflammation T/NT 2 at 2 h. Blood pool uptake 6× than infected muscle at 2 h. Infected muscle uptake lower than most organs.	[91]
Cefprozil	^99m^Tc-cefprozil	*S. aureus*: ~75% at 4 h	Mouse	*S. aureus* myositis	3.7 at 4 h	Sterile inflammation T/NT 2 at 2 h. All organs are at par or higher in uptake than infected muscle.	[92]
Cefuroxime	^99m^Tc-cefuroxime	-	Rat	*S. aureus* myositis	-	No biodistribution data, Scintigraphy showed high thoracic uptake.	[93]
	^99m^Tc-cefuroxime axetil	-	Rat	*S. aureus* myositis	2.5 at 4 h	All organs had higher uptake than infected muscle.	[94]
Ceftriaxone (CRO)	^99m^Tc-CRO	*S. aureus*: 45%, *E. coli*: 70%. All at 4 h	Mouse	*E. coli* myositis	5.6 at 4 h	Plasma protein binding 90%. High intestinal and lung uptake. Sterile inflammation T/NT 1.4, but uptake of normal muscle was 4× of uptake of normal muscle in infected mice. Lung uptake was just as high as infected muscle.	[95]
		-	Rats	*S. aureus* and *E. coli* myositis	*S. aureus*: 2.4; *E.coli*: 12.7. All at 4 h	Sterile inflammation T/NT 1.4 at 4 h. Scintigraphy of *E. coli* myositis did not reflect ex vivo biodistribution; infection focus had a much lower signal, while there were high thoracic and even higher intestinal uptake.	[96]
		*S. aureus*: 45% at 3 h	Mouse	*S. aureus* myositis	2.4 at 4 h	Sterile inflammation T/NT ~1 at 4 h. Normal muscle uptake lower than that of most other organs. Infected thigh uptake comparable to lungs and heart. Scintigraphy done but visually difficult to distinguish infection versus normal muscle.	[97]
		-	Human	-	-	See Table 1.	
Ceftizoxime	^99m^Tc-ceftizoxime	-	Rat	*S. aureus* implant infection	2.0 at 3.5 h	No biodistribution data available. Sternal implant infection not very well seen on scintigraphy.	[98]
		-		*S. aureus* subcutaneous titanium implant infection	-	Abstract has limited information available.	[99]
		-	Human	-	-	See Table 1.	
Cefotaxime	^99m^Tc-cefotaxime	*S. aureus*: 35% at 1 h	Mouse	*S. aureus* myositis	2.9	T/NT ratio collection time unclear. Plasma protein binding 25%. As high or higher organ uptake than infected muscle.	[100]
		-	Rats	*E. coli* myositis	3.8 at 1 h	Sterile inflammation T/NT of 3.3. Cannot distinguish infection from sterile inflammation.	[101]
Ceftazidime	^99m^Tc-ceftazidime	*S. aureus*: 35% at 1 h	Mouse	*S. aureus* myositis	1.1 at 2 h	Ceftazidime is mainly active against Gram negative bacteria, which might contribute to a low T/NT ratio for *S. aureus*. Blood pool uptake higher than infected thigh. Scintigraphy failed to visualize infections.	[102]
Cefoperazone	^99m^Tc-cefoperazone	-	Rat	*S. aureus* myositis	4.7 at 2 h	No sterile inflammation model. Low overall organ uptake contributing to low background signal.	[103]
Cefepime	^99m^Tc-cefepime	*E. coli*: 75% at 12 h	Mouse	*E. coli* myositis	10.0 at 3 h	Sterile inflammation T/NT 3.3, low lung background signal. Infected thigh had higher uptake than most organs except for intestine at 3 h.	[71]
	^99m^Tc-DTPA-cefepime	*S. aureus*: 4% at 6 h	-	-	-	*In vitro* only	[104]
Meropenem	^99m^Tc-meropenem	-	Mouse	*E. coli* myositis	Tumor 4.0 at 1 h and *E. coli* 1.0 at 4 h	Oncology-focused study, cannot differentiate infection from uninfected muscle.	[105]
Ertapenem	^99m^Tc-ertapenem	-	Rat	*S. aureus*, *E. coli* myositis	-	T/NT not calculated. No absolute values given. Biodistribution showed good washout by 4 h. Ex vivo biodistribution indicated *E. coli* seems to have higher uptake than *S. aureus* infected thighs.	[106]
			Rabbit	*S. aureus*, *E. coli* myositis	*S. aureus*: 2.9; *E.coli*: 2.4. All at 4 h	Sterile inflammation T/NT of 1.3, scintigraphy showed promising results with *E. coli* performing better than its ex vivo biodistribution data. Lungs on scintigraphy had much lower signal than infection.	

^99m^Tc: technetium-99m; CRO: ceftriaxone; DTPA: diethylenetriaminepenta-acetic acid.

**Table 4:** Radiolabeled aminoglycosides.

Antibiotic	Ligand	Peak *In Vitro* Binding	Animal Model	Infection Model	T/NT	Notes	Ref.
Gentamicin	^99m^Tc-gentamicin	-	Rat	-	-	Early study in the 1970s, biodistribution only.	[107]
	^125^I-gentamicin	-	-	-	-	Used for radioimmunoassay.	[108]
Tobramycin	^99m^Tc-tobramycin	-	Sheep	-	-	Used to study PK of nebulized tobramycin.	[109]
		-	Rat	-	-	Used to study PK of nebulized tobramycin with pulmonary surfactant as vehicle.	[110]
Kanamycin	^99m^Tc-kanamycin	*S. aureus*: 40% at 4 h	Rat	*S. aureus* myositis	2.4 at 4 h	Normal muscle has comparable uptake to other organs only after 24 h, No sterile inflammation model.	[111]
			Rabbit	*S. aureus* myositis	-	Similar to rat studies, high liver and kidney uptake at 2 h; however infection site was not well noted on image.	
		*S. aureus*: 53%, *E. coli*: 37%. All at 1 h	Mouse	*S. aureus*, *E. coli* myositis	*S. aureus*: 1.8; *E. coli*: 1.8. All at 2 h	No sterile inflammation model used. Study evaluated parameters only up to 2 h post injection with infected muscle uptake lower than most organs. Scintigraphy of *S. aureus* myositis showed minimal elevation of infected muscle compared to normal, and high background signal in the lung/thoracic and abdominal regions of mouse.	[112]
	^177^Lu-kanamycin	-	Mouse	-	-	In addition to the kidneys, all other organs have similar low uptake by 24 h.	[113]
			Rabbit	-	-	Good background signal by 45 min post injection.	
Plazomicin	^99m^Tc-plazomicin	*E. coli*: 79% at 1 h	Mouse	*S. aureus* myositis	7.0 at 4 h	Sterile inflammation T/NT 1.73 at 4 h. Background organ uptake comparable to normal muscle uptake. *C. albicans* and sterile inflammation models had low uptake. Scintigraphy performed showed low background signal except for kidneys and *S. aureus* infection.	[114]
				*C. albicans* myositis	1.9 at 4 h	Performed similarly to sterile inflammation.	
Streptomycin	^99m^Tc-streptomycin	-	Mouse	-	-	Sterile inflammation T/NT of 2.4 at 6 h. High lung uptake, and even higher spleen uptake.	[115]

^99m^Tc: technetium-99m.

**Table 5:** Radiolabeled macrolides.

Antibiotic	Ligand	Peak *In Vitro* Binding	Animal Model	Infection Model	T/NT	Notes	Ref.
Azithromycin	^99m^Tc-azithromycin	*S. aureus*: 65% at 4 h	Mouse	*S. aureus* myositis	6.2 at 2 h	Higher background organ uptake than that in infected tissues.	[116]
Erythromycin	^99m^Tc-erythromycin	*S. aureus*: 50%	Mouse	*S. aureus* myositis	3.5 at 4 h	Cannot distinguish between sterile inflammation and infection. Higher background organ uptake than that in infected tissues.	[117]
Clarithromycin	^99m^Tc-clarithromycin	*S. aureus*: 65% at 1 h	Mouse	*S. aureus* myositis	7.4 at 2 h	Higher background organ uptake than that in infected tissues.	[118]
Roxithromycin	^99m^Tc-roxithromycin	-	Mouse	*S. aureus* myositis	2.9 at 24 h	Very high serum protein binding at 91%. Higher background signal in most organs than in infected tissues.	[119]

^99m^Tc: technetium-99m.

**Table 6:** Radiolabeled tetracyclines.

Antibiotic	Ligand	Peak *In Vitro* Binding	Animal Model	Infection Model	T/NT	Notes	Ref.
Doxycycline	^99m^Tc-doxycycline hyclate	-	Rat	*E. coli* myositis	2.2 at 5 h	No sterile inflammation model tested. Blood pool and myositis uptake were the same.	[120]
	^99m^Tc-doxycycline	90–99% at 24 h	Mouse	*S. aureus* myositis	2.2 at 4 h	Cannot distinguish sterile inflammation from infection.	[121]
			Rabbit	*S. aureus* myositis	3.5 at 4 h	T/NT done through scintigraphy, high thoracic up-take by 4 and 24 h, no sterile inflammation model tested.	
	^177^Lu-doxycycline	-	Mouse	-	-	Tracer was injected intraperitoneally. Utilized in oncology imaging. High uptake for intraabdominal organs after 3 h.	[122]
Tetracycline	^99m^Tc-tetracycline	-	Rat	-	-	High kidney and intestinal uptake, but other organs have relatively low uptake by 24 h.	[123]
	^99m^Tc-oxytetracycline	-		-	-	Lowest uptake in heart and lungs at 0.1% ID/organ compared to the other analogs	
	^99m^Tc-chlotetracycline	-		-	-	Highest background signal among other tetracycline-derived compounds.	
	^99m^Tc-demethylchlortetracycline	-		-	-	More liver accumulation than kidney at 24 h, opposite of oxytetracycline. Low heart and lung uptake.	
	^131^I-tetracycline	-	Rat	*S. aureus* myositis	2.4 at 24 h	No sterile inflammation model used. Very limited biodistribution data.	[124]
Tigecycline	^99m^Tc-tigecycline	-	Rat	*E. coli*, *S. aureus* myositis	*S. aureus*: 2.9; *E. coli*: 2.4	T/NT ratio timing unclear. Sterile inflammation model done but no T/NT ratio reported, only graphs were presented; looks like it cannot distinguish sterile inflammation from infection. High organ background signal.	[125]
			Rabbit	-	-	Scintigraphy in normal uninfected rabbits.	

^99m^Tc: technetium-99m; ^177^Lu: Lutetium-177; ^131^I: Iodine-131.

**Table 7:** Radiolabeled miscellaneous antibiotics.

Antibiotic	Ligand	Peak *In Vitro* Binding	Animal Model	Infection Model	T/NT	Notes	Ref.
Sulfanilamide	^99m^TcN-sulfanilamide ferrocene carboxamide	*S. aureus*: 69%, *E. coli*: 62%. All at 1 h	Mouse	*S. aureus* myositis	*S. aureus*: 2.9 at 30 min	Infection uptake lower than background organ uptake. Blood pool uptake 30× higher; cannot distinguish infection from sterile inflammation	[126]
Sulfadiazine	^99m^Tc-sulfadiazine	-	Mouse	*E. coli* myositis	5.9 at 4 h	No sterile inflammation model. Lung biodistribution was comparable to normal thigh muscle.	[127]
			Rabbit	*E. coli* myositis	-	Scintigraphy showed very high thoracic background signal than infected thigh.	
		-	Mouse	*S. aureus* myositis	3.0 at 1 h	Background organ uptake much higher than infected muscle, with lungs and stomach increasing accumulation over time.	[128]
			Rabbit	Bacillus myositis	2.21 at 1 h	Abstract only available.	[129]
	^18^F-Al-NOTA-sulfadiazine	-	-	-	-	Used for tumor detection. Able to detect tumor well.	[130]
Sulfadimidine	^99m^Tc-sulfadimidine	-	Mouse	*E. coli* myositis	1.5 at 3 h	Sterile inflammation T/NT 1. Infection uptake lower than background organ uptake.	[131]
Clindamycin	^99m^Tc-clindamycin	*S. aureus*: 95–98% at 1 h	Rat	*S. aureus* myositis	*S. aureus*: 2.6 at 4 h	Cannot distinguish sterile inflammation (T/NT 2.02) from infection. High blood pool uptake comparable to infection even at 24 h. Scintigraphy shows low abdominal background.	[132]
Lincomycin	^99m^Tc-lincomycin	*S. aureus*: 99%, *E. coli*: 84%. All at 4 h	Rat	*S. aureus* myositis	*S. aureus*: 1.5 at 4 h	Sterile inflammation T/NT 1.2. While infected thigh has higher uptake than most organs, it is not by much.	[133]
			Rabbit	*S. aureus* myositis	-	Authors presented joint scintigraphy in rabbits, with surrounding muscle demonstrating moderate background signal.	
Trimethoprim	^11^C-trimethoprim	-	Mouse	-	-	Very short half-life. Used for tumor imaging but demonstrated high intestinal uptake.	[134]
		-	Human	-	-	See Table 1	
	^18^F-fluoropropyl-trimethoprim	-	Mouse	*E. coli*, *S. aureus*, *P. aeruginosa* myositis	*E. coli*: 2.5; *S. aureus*: ~1, *P. aeruginosa*: 4.0. All at 2 h	T/NT ratio obtained via PET. Sterile inflammation T/NT 1. Tumor T/NT 1.3. Sterile inflammation from *P. aeruginosa* infected mice was ~3. High small intestinal uptake, but very low lung and other organ uptake.	[135]
			Rhesus monkeys	-	-	Not an infection model. Heart had increasing uptake over time, while lung uptake remained low.	
Metronidazole	^99m^Tc-metronidazole	-	Rat	*E. coli* myositis	5.5 at 24 h	T/NT calculated using infected and sterile inflamed muscles, low organ background uptake.	[136]
		-	Rabbit	*E. coli* myositis	-	Scintigraphy showed high thoracic background signal and no discernible difference between infection and sterile inflammation.	
	^99m^TcN-PNP5-metronidazole-DTC	-	Mouse	-	-	Used for tumor hypoxia measurement but has adequate washout by 4 h; however lung background signal was as high as tumor signal.	[137]
Nitrofurantoin	^99m^Tc-nitrofurantoin	*E. coli*: 50–65% at 1 h	Rat	*E. coli* myositis	3.7 at 2 h	Blood pool uptake higher than in some organ. Sterile inflammation T/NT of 1. No lung biodistribution data.	[138]
			Rabbit	*E. coli* myositis	-	Scintigraphy at 1 h showed good localization of *E. coli* infection, though thoracic and abdominal background higher than desired.	
	^125^I-nitrofurantoin	-	Mouse	-	-	Biodistribution showed lower lung uptake, but very high intestinal uptake. However, blood pool values remained high after 1.5 h. No infection model used.	[139]
Polymyxin B	^99m^Tc-polymyxin B	*E. coli*: 36%; *P. aeruginosa*: 31.5%; *A. baumanii*: 37.4%; *K. pneumoniae*: 45%; *S. aureus*: 15.9%; *E. faecalis*: 18.5%. All at 1 h	Mouse	*E. coli*, *P. aeruginosa*, *A. baumanii*, *S. aureus*, *E. faecalis* myositis	*E. coli*: 4.5; *P. aeruginosa*: 4; *A. baumanii*: 4; *S. aureus*: 2.5; *E. faecalis*: 2.5. All at 6 h	All infection loci are reported as T/NT ratios. No sterile inflammation model. Organ background 2–5 times normal muscle uptake at 6 h. Blood pool uptake remains higher than most organs at 6 h.	[140]
Colistin	^99m^Tc-colistin	-	Mouse	-	-	Serum binding 30%, no infection model used. High background signal in the abdomen via scintigraphy, but low ex vivo intestinal uptake.	[141]
	^177^Lu-colistin	-	-	-	-	Synthesized.	[142]
Linezolid	^131^I-linezolid	-	Rat	*S. aureus* myositis	*S. aureus*: 11.1 at 1 h	Sterile inflammation T/NT 3. No uptake values for uninfected muscle compared to other organs. Lung had low uptake, while stomach had very high uptake.	[143]
	^18^F-linezolid	-	Rat	*M. tuberculosis* pneumonia	-	No T/NT ratio reported. Pharmacokinetic study. Good penetration into infected foci.	[144]
Vancomycin	^201^Tl-vancomycin	-	Rat	-	-	Biodistribution only	[145]
	^18^F-BODIPY-FL-vancomycin	*E. faecalis*, *S. captis*, *S. aureus*, *S. epidermidis*: ~37–62%; *C. acnes*: <20%. All at 30 min	Mouse	*S. aureus*, *E. coli* myositis	*S. aureus*: 3.0; *E. coli*: 2.7. All at 1 h	Sterile inflammation T/NT 1.91. Gram negative bacteria (four species) showed <5% *in vitro* binding. Blood pool uptake was higher than infection, high lung uptake.	[146,147]
	^18^F-PQ-VE1-vancomycin	*E. faecalis*, *S. captis*, *S. aureus*, *S. epidermidis*: 37–65%; *C. acnes*: ~35%. All at 30 min	Mouse	*S. aureus*, *E. coli* myositis	*S. aureus*: 1.5; *E. coli*: 1.2. All at 1 h	Sterile inflammation T/NT 1.23. Gram negative bacteria (four species) showed <5% *in vitro* binding; cannot distinguish infection from sterile inflammation. Higher background signal than FDG, higher blood pool uptake than in infection site.	
	^18^F-FB-vancomycin	-	-	-	-	Rapidly degraded *in vitro* and *in vivo*, only urine accumulation was observed.	
	^55^Co(II)-Vancomycin	-	-	-	-	Synthesized. Abstract only.	[148]
	^99m^Tc-vancomycin	-	Rat	*S. aureus* endocarditis	-	Synthesized. Abstract only with no T/NT reported.	[149]
Tazobactam	^99m^Tc-tazobactram	-	Rat	*P. aeruginosa*, *S. enterica* myositis	*P. aeruginosa*: 10.3; *S. enterica*: 7.6. All at 2 h	Sterile inflammation T/NT 1.3. Organ biodistribution improved after 24 h. Lung uptake similar to normal muscles.	[150]
			Rabbit	*P. aeruginosa*, *S. enterica* myositis	-	Scintigraphy showed slightly higher *P. aeruginosa* signal than *S. enterica*. However, thoracic background was quite high at 2 h, visually similar to infection signal.	

^99m^Tc: technetium-99m; ^99m^TcN: technetium-99m nitrido; ^11^C: Carbon-11; ^125^I: Iodine-125; ^131^I: Iodine-131; ^177^Lu: lutetium-177; ^201^Tl: thallium-201; ^18^F: fluorine-18; ^55^Co: cobalt-55; BODIPY-FL: Boron-Dipyrromethene-Fluorine; PNP5: bis-dimethoxypropylphosphinoethyl-ethoxyethylamine; PQ-VE1: 9,10-phenanthrenequinone-vinyl ether 1; FB: fluoroborate; NOTA: 1,4,7-triazacyclononane-1,4,7-triacetic acid; Al: aluminum.

**Table 8:** Radiolabeled antimycobacterials.

Antibiotic	Ligand	Peak *In Vitro* Binding	Animal Model	Infection Model	T/NT	Notes	Ref.
Isoniazid	^99m^Tc-isoniazid	-	Rabbit	*M. tuberculosis* myositis	2.0 at 2 h	No sterile inflammation reported, and no organ distribution reported.	[151]
	^99m^T(CO)_3_-isoniazid	*M. tuberculosis*: No binding	-	-	-	No *in vitro* binding, not further pursued.	
	^99m^Tc-HYNIC-isoniazid	*M. tuberculosis*: No binding	-	-	-	No *in vitro* binding, not further pursued.	
	^18^F-fluoroisonicotinic acid hydrazide	*M. tuberculosis*: 100% at 8 h	Mouse	*M. tuberculosis* pneumonia	1.7 at 40 min	T/NT ratio obtained from PET imaging. No sterile inflammation model used. No direct correlation between uptake and MTB lesions.	[152]
	^99m^Tc-alginate-isoniazid	-	Rabbit	-	-	Drug biodistribution study. Blood pool still retained a significant amount of radiotracer after 24 h.	[153]
Rifampin	^11^C-rifampin	-	Mouse	*M. tuberculosis* pneumonia (PK)	-	Drug PK study. Necrotic lung *M. tuberculosis* lesions had significantly lower drug penetration compared to uninfected lung tissue (63%).	[154]
		-	Rabbit	*M. tuberculosis* meningitis (PK)	-	Drug PK study. Rifampin penetration into infected brain lesions was limited and spatially heterogeneous.	[27]
		-	Mouse	*S. aureus* bone implant (PK)	-	Drug PK study. Higher rifampin dose increased drug penetration, and 3 weeks of vancomycin and high rifampin dosing was non-inferior to 6 weeks of vancomycin and standard rifampin dosing in *S. aureus* bone implant infection murine model.	[26]
		-	Human	-	-	See Table 1.	
	^99m^Tc-rifampin	-	Rat	MRSA myositis	5.7 at 2 h	Sterile inflammation T/NT of 1. No lung biodistribution done. Biodistribution similar to ^99m^Tc-rufloxacin.	[155]
			Rabbit	MRSA myositis	-	Distinguishes infection vs. inflammation well (not quantified), though high thoracic background signal was observed.	
Rifabutin	^99m^Tc-rifabutin	*M. tuberculosis*: ~60% at 1.5 h	Rat	*M. tuberculosis* myositis	4.3 at 2 h	Sterile inflammation T/NT of 1.1. No lung biodistribution done. Biodistribution similar to ^99m^Tc(CO)_3_-tosufloxacin-DTC.	[156]
	^99m^Tc(CO)_3_-rifabutin-DTC	*M. tuberculosis*: ~50% at 1.5 h	Rat	*M. tuberculosis* myositis	4.3 at 2 h	Sterile inflammation T/NT of 1. No lung biodistribution done. Biodistribution similar to ^99m^Tc(CO)_3_-tosufloxacin-DTC.	[157]
			Rabbit	*M. tuberculosis* myositis	-	Scintigraphy showed high thoracic background, comparable to infected muscle.	
Pretomanid	^18^F-pretomanid	*M. tuberculosis*: 75–83% at 3 h	Mouse	*M. tuberculosis* meningitis (PK)	-	Serum protein binding 74–83% at 3 h. Drug PK study. Brain MTB lesions had lower drug penetration than non-infectious lesions in brain parenchyma and lung.	[33]
			Rabbit	*M. tuberculosis* meningitis (PK)	-	Very low penetration into infected brain lesions.	
		-	Human	-	-	See Table 1.	
Bedaquiline	^76^Br-bedaquiline	-	Mouse	*M. tuberculosis* pneumonia (PK)	-	Poor penetration into infected lung lesions.	[158]
Pyrazinamide	^18^F-pyrazinamide	-	Mouse	*M. tuberculosis* pneumonia	-	Findings suggest rapid defluorination *in vivo*, plateauing after 1 h at 40% defluorination.	[159]
Ethambutol	^99m^Tc-ethambutol	*M. tuberculosis*: ~70% at 1.5 h	Mouse	*M. tuberculosis* myositis	1.8 at 4 h	No mention of lung biodistribution, and no sterile inflammation model.	[160]
			Rabbit	*M. tuberculosis* myositis	-	Scintigraphy showed good localization of infected muscle, but thoracic background looked high at 2 h.	
		-	Human	-	-	See Table 1.	

^99m^Tc: technetium-99m; ^99m^T(CO)_3_: technetium-99m carbonyl; ^11^C: Carbon-11; ^18^F: fluorine-18; ^76^Br: bromine-76; HYNIC: 6-hydrazinonicotinic acid; (PK): indicating PK study to evaluate drug penetration into infected tissues; MRSA: Methicillin-resistant staphylococcus aureus.

**Table 9:** Radiolabeled antivirals.

Antiviral	Ligand	Animal Model	Infection Model	T/NT	Notes	Ref.
Ganciclovir	^18^F-FHPG	Rat	HSV encephalitis	3.2 at 55 min	Rats were infected via nostril inhalation. Olfactory region had the highest T/NT uptake of 3.2 at 55 min. No other biodistribution noted. No other viruses used for control.	[161]
		-	-	-	*In vitro* study comparing non-infected versus CMV infected cells showed targeted accumulation in infected cells.	[162]
	^18^F-FHBG	Rat	-	-	Detection of transgenic tumors expressing HSV1-thymidine kinase that were implanted in rats.	[163]
		Human	-	-	See Table 1.	
Penciclovir	^18^F-FPCV	Mouse	-	-	Detection of transgenic tumors expressing HSV1-thymidine kinase that were implanted in mice.	[164]
Dolutegravir	^18^F-dolutegravir	Rhesus macaques	-	-	Confirmed elimination route through the liver, gall bladder and kidneys. Also confirmed low CNS penetration in mouse studies.	[165]
Tenofovir	^18^F-FPMPA	Rat	-	-	PK study largely mirroring rat ^14^C-PMPA distribution with exception of lungs and kidneys.	[166]
Oseltamivir	^11^C-oseltamivir	Japanese macaque	-	-	Sterile inflammation model used to simulate a viral infection profile. All measured parameters—brain concentration, brain-to-plasma concentration ratio, and plasma-to-brain transfer rate—were unchanged from inflammation. Not used for infection diagnosis.	[167]

^18^F: fluorine-18; ^11^C: carbon-11; FHPG: (9-[(1-[^18^F]Fluoro-3-hydroxy-2-propoxy)methyl]guanine); FHBG: 9-(4-^18^F-fluoro-3-[hydroxymethyl]butyl)guanine; FPMPA: *S*-(1-(6-amino-9H-purin-9-yl)-3-fluoropropan-2-yloxy)methylphosphonic acid; FPCV: 8-[^18^F] fluoropenciclovir; HSV: Herpes Simplex Virus; CMV: Cytomegalovirus.

**Table 10:** Radiolabeled antifungals.

Antifungal	Ligand	Peak *In Vitro* Binding	Animal Model	Infection Model	T/NT	Notes	Ref.
Fluconazole	^99m^Tc-fluconazole	*C. albicans*: 38%; *A. fumigatus*: 18%, mammalian: 12%. All at 1 h	Mouse	*C. albicans*, *A. fumigatus* myositis	*C. albicans*: ~3.5; *A. fumigatus*: ~1.5. All at 2 h	Sterile inflammation T/NT ~1.5 at 2 h. Biodistribution only evaluated bladder and liver. Scintigraphy showed low uptake, and very high abdominal background signal.	[168]
	^99m^Tc-Fluconazole-PLA-POLOX	-	Mouse	*C. albicans* myositis	AUC T/NT 1.6 after 4 h	~4× Higher blood pool uptake at 4 h than ^99m^Tc-fluconazole. ~2× higher liver uptake than parent compound. Low lung uptake. No sterile inflammation model.	[169]
	^99m^Tc-Fluconazole-PLA-PEG	-			AUC T/NT 1.5 after 4 h	~5× Higher blood pool uptake at 4 h than ^99m^Tc-fluconazole. ~2× higher liver uptake than parent compound. No sterile inflammation model. Low lung uptake.	
	^18^F-Fluconazole	-	Rabbit	*C. albicans* myositis	AUC T/NT was ~1.3 after 2 h	Liver, muscles, blood, and other organs assessed had similar uptake by 2 h. No sterile inflammation model.	[170]
		-	Human	-	-	See Table 1.	
Caspofungin	^99m^Tc(CO)_3_-caspofungin	-	Mouse	*C. albicans*, *A. niger* myositis	Biodistribution: *C. albicans*: 5.1; *A. niger*: 3.6. All at 12 h. Scintigraphy: *C. albicans*: 9.5; *A. niger*: 13.4. All at 12 h	Plasma protein binding 78.7%. Sterile inflammation T/NT 1.1. Blood pool uptake was comparable to, if not higher than most organs except for liver/kidney even after 12 h. Scintigraphy appeared more promising than biodistribution data, especially for *A. niger* infection.	[171]
Anidulafungin	^99m^Tc(CO)_3_-anidulafungin	-	Mouse	*S. aureus*, *C. albicans*, *A. fumigatus* myositis	*S. aureus*: 1.6; *C. albicans*: 5.9; *A. fumigatus*: 6.3. All at 6 h	Plasma protein binding 77%. Sterile inflammation T/NT 1.4 at 6 h. Blood pool remains higher than in most organs at 6 h. Lung uptake was comparable to normal muscle uptake. Infection uptake was higher than all organ uptake by significant amount. No scintigraphy done.	[172]
Amphotericin B (AmB)	^99m^Tc-AmB	*A. fumigatus*: 1.1%; *R. oryzae*: 0.2%. All at 2 h	-	-	-	In vitro data only. The manuscript’s Supplementary Materials showed that a wide variety of mold/fungi had higher accumulation with ^68^Ga-AmB than ^99m^Tc-AmB.	[173]
	^68^Ga-AmB	*A. fumigatus* and *R. oryzae*: ~1.1%. All at 2 h					
	^99m^Tc(CO)_3_-AmB	*C. albicans*: 39.9% at 1 h	Mouse	*C. albicans*, *A. niger* myositis	*C. albicans*: 4.7; *A. niger*: 2.4	T/NT ratio collection time unclear (3 or 6 h). Plasma protein binding 77.6%, sterile inflammation T/NT 1.5. Blood pool had higher uptake than most organs tested, except for liver and bladder. No lung distribution tested.	[174]
	^99m^TcN-AmB	*C. albicans*: 14.2% at 1 h	-	-	-	Plasma protein binding 53.0%. No *in vivo* studies done.	

^99m^Tc: technetium-99m; ^18^F: fluorine-18; ^68^Ga: gallium-68; PEG: poly(ethylene glycol); PLA: poly(D,L-lactic acid); POLOX: poloxamer.

**Table 11:** Criteria for evaluating translational potential radiolabeled antimicrobial tracers.

Criteria	Definition
1. *In vivo* nuclear imaging	SPECT or PET imaging suggesting favorable performance with good target-to-non-target ratio. Preferably in multiple animals.
2. Sterile inflammation model	Sterile inflammation control with T/NT ratio approaching 1
3. Non-target pathogen model	Infection model using a non-target pathogen demonstrates appropriately low uptake
4. Target signal retention	Sustained or increasing target tissue uptake over time. Uptake rate = (last %ID/g − 1st %ID/g)/(last timepoint − 1st timepoint)
5. Complete organ biodistribution	Favorable biodistribution data including vital organs
6. Multiple target infection models	Tracer evaluated in more than one target infection model
Optional: Pharmacokinetic parameters	Tracer pharmacokinetic data considered when available

**Table 12:** Tracers with higher translation potential.

**Drug Name**	Plazomicin	Caspofungin	Anidulafungin	Trimethoprim
**Radioligand**	^99m^Tc-plazomicin	^99m^Tc(CO)_3_-caspofungin	^99m^Tc(CO)_3_-anidulafungin	^18^F-fluoropropyl-trimethoprim
**Reference**	[114]	[171]	[172]	[135]
**Injection Dose**	35 MBq	0.37–1.1 MBq	150 MBq	370 MBq
**Infection Model**	*S. aureus* myositis	*C. albicans*, *A. niger* myositis	*C. albicans*, *A. fumigatus* myositis	*E. coli*, *S. aureus*, *P. aeruginosa* myositis
**Nuclear Imaging Done**	Planar SPECT	SPECT/CT	Planar SPECT	PET/CT
**Imaging clearly showing area of uptake?**	Yes	Yes	Yes	Yes
**T/NT based** **on imaging**	No	Yes *C. albicans*: 9.5 (12 h) *A. niger*: 13.4 (12 h)	No	Yes *E. coli*: 2.8 (2 h) *S. aureus*: 1 (2 h)
**Target Signal %ID/g** **(hrs post injection)**	7.8 (4 h)	*C. albicans*: 5.6 (12 h) *A. niger*: 2.9 (12 h)	*C. albicans*: 5.8 (6 h) *A. fumigatus*: 5 (6 h)	-
**Target Signal Retention** **(%ID/g per hr)**	+0.25	*C. albicans*: −0.01 *A. niger*: +0.12	*C. albicans*: +0.05 *A. fumigatus*: −0.03	-
**Complete ex vivo biodistribution**	Yes	Yes	Yes	Yes
**T/NT based on biodistribution** **(hrs post injection)**	7 (4 h)	*C. albicans*: 6.9 (12 h) *A. niger*: 3.6 (12 h)	*C. albicans*: 6.4 (6 h) A. fumigatus: 6.25 (6 h)	-
**Number of Target Inflammation model(s) used**	1, *S. aureus*	2, *C. albicans* and *A. niger*	2, *C. albicans* and *A. fumigatus*	2, *E. coli* and *S. aureus*
**Other non-target infectious inflammation model (name, T/NT, hrs post injection)**	Yes (*C. albicans*, 1.9, 4 h)	No	Yes (*S. aureus*, 1.6, 6 h)	Yes (*P. aeruginosa*, 3, 2 h)
**Sterile inflammation**	Yes	Yes	Yes	Yes
**Washout kinetics (blood)**	Described. First order kinetic, 2.6 %ID/g 4 h post-injection	Not described. 5.7 %ID/g 12 h post injection	First order kinetic, 3.6 and 1.6 %ID/g at 4, 6 h post injection, respectively	-

## Data Availability

No new data were created or analyzed in this study. Data sharing is not applicable to this article.

## Supplementary Materials

The following supporting information can be downloaded at: https://www.mdpi.com/article/10.3390/ijms27125313/s1. References [32,33,39,40,41,43,44,45,46,47,48,49,54,57,59,60,66,71,86,88,89,91,93,95,96,98,101,102,105,107,109,110,111,112,113,115,116,117,118,119,120,121,123,125,126,128,131,132,138,139,140,141,143,144,145,146,147,154,158,159,165,166,167,168,169,170,174,185,186,187,188,189,190,191,192,193,194,195,196,197] are cited in the Supplementary Materials.
