# Supplementary material for: Radiolabeled Antimicrobials for Infection Imaging: A Scoping Review
Source: Int J Mol Sci. 2026 Jun 11;27(12):5313. doi: 10.3390/ijms27125313 (PMC13299976; doi:10.3390/ijms27125313)
Supplement: Supplementary file 1 [file ijms-27-05313-s001.zip › Supplemental Tables.pdf]

Supplementary Table S1. Antimicrobial Names.

Drug Class	Subclass	Drug Names	Example Search (Publish or Perish)
Fluoroquinolones	2nd generation	Ciprofloxacin, norfloxacin, ofloxacin, pefloxacin, lomefloxacin, fleroxacin, rufloxacin, enrofloxacin, sarafloxacin, danofloxacin, difloxacin, pazufloxacin, temafloxacin	"radiolabeled ciprofloxacin" in keyword field
	3rd generation	Levofloxacin, sparfloxacin, gatifloxacin, tosufloxacin, prulifloxacin	
	4th generation	Moxifloxacin, gemifloxacin, trovafloxacin, clinafloxacin, sitafloxacin, garenoxacin	
	5th generation	Delafloxacin, nemonoxacin	
Beta-lactams	Penicillins	Benzylpenicillin, amoxicillin, oxacillin, naficillin	"radiolabeled amoxicillin" in keyword field
	Cephalosporins, 1st generation	Cefazolin, cephalexin, cephadrine, cefonicid	
	Cephalosporins, 2nd generation	Cefuroxime, cefaclor, cefprozil, cefamandole, cefoxitin, cefotetan, cefmetazole	
	Cephalosporins, 3rd generation	Ceftriaxone, cefotaxime, ceftizoxime, cefpodoxime, cefixime, ceftazidime, cefoperazone	
	Cephalosporins, 4th generation	Cefepime, cefpirome, cefozopran	
	Carbapenems	Imipenem, meropenem, ertapenem, doripenem	
Aminoglycosides	—	Gentamicin, tobramycin, kanamycin, plazomicin, streptomycin	
Macrolides	—	Azithromycin, erythromycin, clarithromycin, roxithromycin	
Tetracyclines	—	Doxycycline, tetracycline, tigecycline, eravacycline, omadacycline, minocycline, demeclocycline, lymecycline, oxytetracycline, sarecycline	
Miscellaneous Antimicrobials	—	Sulfanilamide, sulfadiazine, sulfadimidine, clindamycin, lincomycin, trimethoprim, metronidazole, nitrofurantoin, polymyxin B, colistin, linezolid, vancomycin, tazobactam, daptomycin	
Anti-mycobacterials	—	Isoniazid, rifampin, rifabutin, pretomanid, bedaquiline, pyrazinamide, ethambutol	
Antivirals	—	Ganciclovir, penciclovir, dolutegravir, bictegravir, tenofovir, oseltamivir	"radiolabeled ganciclovir" in keyword field; also searched "radiolabeled antivirals" to broaden search
Antifungals	—	Fluconazole, posaconazole, voriconazole, itraconazole, isavuconazole, caspofungin, anidulafungin, micafungin, amphotericin B, ibrexafungerp, olorofim, rizafungin	

List of all antimicrobial names used for literature search organized by class, generation (if applicable). An example of search phrase used in Publish or Perish software also provided for Fluoroquinolones, Beta-lactams, and Antivirals.

Supplementary Table S2. Radioisotope Names.

SPECT Radioisotopes	PET Radioisotopes
67Ga: Gallium-67	11C: Carbon-11
99mTc: Technetium-99m	13N: Nitrogen-13
111In: Indium-111	18F: Fluorine-18
125I: Iodine-125	64Cu: Copper-64
131I: Iodine-131	68Ga: Gallium-68
177Lu: Lutetium-177	89Zr: Zirconium-89

List of all SPECT and PET enabled radioisotopes used in the screening process.

Supplementary Table S3. Radioisotope Breakdown of Existing Research.

Radioisotope	Subtype	Radioligands	Animal Studies	Clinical Evaluation
**^{99m}Tc**	Plain ^{99m}Tc-	71	95	14
	$^{99m}Tc(CO)_3$- (carbonyl)	20	19	0
	^{99m}TcN- (nitrido)	13	13	0
	^{99m}Tc(V)O-	1	1	0
**^{99m}Tc Subtotal**		**105**	**128**	**14**
^{18}F	—	21	18	8
^{11}C	—	4	5	3
^{177}Lu	—	4	4	0
^{68}Ga	—	3	2	0
^{125}I	—	2	2	0
^{131}I	—	2	1	0
^{76}Br	—	1	1	0
^{201}Tl	—	1	1	0
^{55}Co	—	1	0	0
^{65}Ga	—	1	0	0

Breakdown of all radioligands that were synthesized shown by radioisotopes, preclinical studies, and clinical evaluations.

Supplementary Table S4. Number of Preclinical Research Articles by Decade .

Decade	Preclinical Article Count	Clinical Article Count
1970s	3	0
1980s	1	0
1990s	7	5
2000s	23	10
2010s	86	5
2020 - Present	23	5

Count of preclinical and clinical research articles included in this review by decade of publication. .
