# Supplementary material for: Radiolabeled Antimicrobials for Infection Imaging: A Scoping Review
Source: Int J Mol Sci. 2026 Jun 11;27(12):5313. doi: 10.3390/ijms27125313 (PMC13299976; doi:10.3390/ijms27125313)
Supplement: Supplementary file 1 [file ijms-27-05313-s001.zip › Supplementary Note.pdf]

Supplementary Note

Antibacterials

Fluoroquinolones

Fluoroquinolones, which target the bacterial DNA gyrase and topoisomerase IV, constitute the antibiotic class most broadly explored as potential radioligands. Radiolabeled derivatives of second-, third-, and fourth-generation fluoroquinolones have been synthesized, with several advancing to clinical evaluation, whereas no reports were identified for radiolabeled first- or fifth-generation compounds. As fluoroquinolones are renally excreted, their radiolabeled forms show high kidney and bladder uptake, as well as variable liver uptake due to hepatic metabolism.

Second Generation Fluoroquinolones (FQ)

Besides ^{99m}Tc-CIP (Infecton) which was evaluated in clinical trials, CIP has been synthesized with other conjugates but each had shortcomings (Table 2) [39, 40, 41, 43-47]. Lomefloxacin and ofloxacin demonstrated similarly low background uptake across normal organs, but the authors did not report the post-injection time point at which biodistribution was measured. Infected-muscle uptake for both tracers was less than twofold higher than lung activity, suggesting limited contrast [54]. Given the large number of other fluoroquinolone-based tracers already reported with more favorable performance, further development of these ligands appears to be a lower priority. Other FQs of this generation either had high background seen on scintigraphy, cannot localize to infection or cannot distinguish infection from sterile inflammation [48, 49, 57].

Third Generation Fluoroquinolones:

^{99m}Tc-Levofloxacin, which also reached the clinical evaluation phase, initially showed its promise for detection of the gram positive bacteria *S. aureus* in a rat myositis model [59]. Amongst gram negative bacteria, preclinical studies demonstrated better detection for detection of *P. aeruginosa* than S. typhi or *E. coli* [60, 185]. Other not yet mentioned radiolabeled FQ in this generation is ^{99m}Tc-sparfloxacin had limited data available [66].

Fourth Generation Fluoroquinolones:

Unlike previous generations of FQ, no fourth generation FQs reached clinical evaluation. ^{99m}Tc-prulifloxacin underwent scintigraphy that showed good distinction of infected thigh versus normal thigh, but thoracic area exhibited high background which might not be good for pneumonia detection [86]. Furthermore, while trovafloxacin technetium-99m conjugates showed some promise, ^{18}F-trovafloxacin had decrease ligand accumulation in the infected muscle[32]. ^{99m}Tc-gatifloxacin were unable to detect *E. coli* myositis[71].

Beta lactams

Beta-lactam antibiotics, named for the characteristic beta-lactam ring in their structure, inhibit penicillin binding proteins, which catalyze cross-linking of peptidoglycan chains required for bacterial cell wall synthesis [186]. Like quinolones, most beta lactams are renally excreted with some liver metabolism, so demonstrate high activity in the urinary tract and in the liver.

Penicillin

Penicillin G (benzylpenicillin) has been labeled with Lutetium-177 and Technetium-99m for pharmacokinetic studies. It demonstrated the expected renal accumulation followed by rapid clearance [88, 187]. Amoxicillin tagged with Technetium-99m showed higher serum protein binding than its parent molecule. However, lack of biodistribution data

from the lungs precludes predictions about potential performance in pneumonia [89]. Other *S. aureus* targeting penicillin such as oxacillin and nafcillin have not been radiolabeled for diagnostic applications.

Cephalosporins

Cephalosporins also contain a β-lactam ring but are fused with a dihydrothiazine ring instead of the thiazolidine ring of penicillin. Technitium-99m radiolabeled first generation and second generation cephalosporins including cefazolin, cefuroxime, and cefuroxime axetil all showed unfavorably high background organ uptake exceeding that of target infected thigh muscle [91, 93, 188]. Other second generation cephalosporins like cefoxitin and cefotetan have not yet been labeled. Ceftriaxone, a third-generation cephalosporin, was labeled as ^{99m}Tc-ceftriaxone. It demonstrated superior uptake in a rat *E. coli* myositis model compared to *S. aureus* infection [95]. However, scintigraphy and ex vivo biodistribution studies were discordant with scintigraphy showing high lung/thoracic uptake with low infection uptake, which will limit clinical infection detection [96]. ^{99m}Tc-ceftizoxime localized *S. aureus* implant infection but scintigraphy results were not impressive [98]. In contrast, several studies of ^{99m}Tc-cefotaxime and ^{99m}Tc-ceftazidime both had difficulties in distinguishing infections [101, 102]. Of note, ceftazidime targets gram-negative bacteria, so future investigators should utilize gram-negative pathogens to induce myositis to fully assess its radiotracer capabilities [102].

Carbapenems

Carbapenems are a class of beta-lactam antibiotics that have very broad spectrum of activity, especially among antibiotic resistant bacteria. Amongst the carbapenems, meropenem and ertapenem have been radiolabeled. Exploration of ^{99m}Tc–meropenem by Sakr et al showed promise for detection of tumor hypoxia, but poor performance for identification or *E. coli* infection in a mouse model [105]. Consideration of other carbapenems such as imipenem, and doripenem as potential radioligands might be fruitful.

Aminoglycosides

^{99m}Tc-gentamicin has been synthesized and suggested for applications to kidney imaging [107]. However, no further development, including fluorine labeling, towards use in infection imaging was pursued. ^{99m}Tc-tobramycin also has not been tested in infection settings, though it has been used to measure lung clearance in rats and to assess biodistribution in sheep [109, 110]. ^{99m}Tc-streptomycin has been synthesized but only tested in sterile inflammation models and showed a T/NT of 2.4. Relative to other labeled aminoglycosides, uptake in other organs was higher than that of inflammation [115]. On the other hand, Roohi et al. evaluated 99mTc-kanamycin in an *S. aureus* myositis model and observed appreciable organ washout only after 24 hours [111]. Additionally, the scintigraphy performed did not clearly show an clear uptake by the infected muscle[111]. Consistent with these findings, Widyasari et al. reported unimpressive performance of the ligand in both *S. aureus* and *E. coli* myositis models; scintigraphy showed high organ background with only minimal signal increase at the *S. aureus* infection site [112]. ^{177}Lu-kanamycin showed lower hepatic background than ^{99m}Tc-plazomicin in mice, but was also not advanced to infection models [113]. Please refer to table 4 for more detailed information for each study.

Macrolides

Macrolides bind to the 50S subunit of bacterial ribosomes to inhibit protein synthesis. Macrolides can be used to treat some intracellular pathogens due to their ability to rapidly accumulate inside host cells [189]. The first macrolide, erythromycin, and subsequent derivatives, have been labeled with technetium-99m. ^{99m}Tc-erythromycin, ^{99m}Tc-azithromycin, ^{99m}Tc-clarithromycin, and ^{99m}Tc-roxithromycin all showed disappointingly high background organ uptake which exceeded that of infected muscle [115-119]. Although their myositis T/NT ratios were comparable to the ^{99m}Tc-CIP T/NT,

these compounds may penetrate muscle poorly. Additionally, labeled erythromycin had overall low uptake values and did not distinguish sterile inflammation from infection [117]. There is no radiolabeled version of newly developed nafithromycin. This antibiotic class rapidly accumulates in host cells, which likely explains the particularly high background signal, making it appear suboptimal for infectious disease imaging (Table 5).

Tetracyclines

The tetracycline class inhibits the 30S ribosomal subunit and has diverse indications, including infectious disease, dermatologic, and rheumatologic applications. Like the macrolides, it also exhibits rapid accumulation within host cells [190]. Radiolabeled forms have undergone limited evaluation for detection of infection (Table 6). While Technitium-99m labeled doxycycline hyclate showed lower background organ uptake than ^{99m}Tc-doxycycline [120, 121], it did not distinguish sterile inflammation from infection; ^{99m}Tc-doycycline hyclate studies did not include a sterile inflammation model. Similar to radiolabeled doxycycline, ^{99m}Tc-tigecycline was unable to distinguish infection from inflammation [125]. Biodistribution studies of ^{99m}Tc-tetracycline and related analogs have also been performed. Of particular interest, ^{99m}Tc-oxytetracycline exhibited minimal residual pulmonary activity at two hours, suggesting potential for further evaluation in pneumonia models [123]. Other tetracyclines such as minocycline, omadacycline, eravacycline, and sarecycline have not been radiolabeled. Given the track record of other labeled tetracyclines, future investigators should prioritize other antibiotic classes over tetracyclines. Please refer to table 6 for more detailed information for each study.

Miscellaneous Antibiotics

This section discusses antibacterials with various mechanisms of action. Unlike the previously discussed classes, these represent diverse mechanisms with fewer radiolabeled examples per category.

Glycopeptides

Vancomycin is a glycopeptide antibiotic known for its activity against Methicillin-Resistant Staphylococcus aureus (MRSA) and gram-positive bacteria. Spoelstra et al. produced several radiolabeled vancomycin derivatives, including ^{18}F-FB-vancomycin, ^{18}F-BODIPY-FL–vancomycin, and ^{18}F-PQ-VE1–vancomycin [147], the latter two of which showed selective binding to gram positive bacteria *in vitro*. In *S. aureus* and *E. coli* murine myositis models, ^{18}F-BODIPY-FL-vancomycin bound *E. coli* at 60 min post injection, but biodistribution revealed lower uptake by infected muscles than blood pool and high background organ activity. In summary, ^{18}F-BODIPY-FL-vancomycin can distinguish *S. aureus* and *E. coli* from sterile inflammation, and ^{18}F-PQ-VE1-vancomycin selectively accumulated in *S. aureus* compared to *E. coli* infection but had higher background than ^{18}F-FDG [146]. ^{201}Tl-Tl(III)-Vancomycin was synthesized but never used in infection models [145].

Sulfonamides

Sulfonamides act as competitive inhibitors of dihydropteroate synthase. Several radiolabeled derivatives have been synthesized such as ^{99m}Tc-sulfadiazine, ^{99m}Tc-sulfadimidine, and ^{99m}Tc-N-sulfanilamide, but their application is limited due to high background organ uptake that exceeds uptake in infected muscle [126, 128, 131].

Nitrofurantoin

Nitrofurantoin in its reduced form damages bacterial DNA, ribosomes and other macromolecules. Because bacterial cells activate the drug more efficiently than mammalian cells, its effects are more pronounced in bacteria. This antibiotic concentrates particularly well urine. In a rat *E. coli* myositis model, ^{99m}Tc-nitrofurantoin showed higher uptake in most organs and blood pool compared to uninfected muscle. In a rabbit model, scintigraphy showed higher uptake by

https://doi.org/10.3390/ijms27125313

infected muscle, though the thoracic and abdominal regions showed high background [138]. ^{125}I-nitrofurantoin in healthy mice showed very low lung uptake, which suggests potential for pneumonia detection [139]. Additional evaluation in clinically relevant models is needed to define the diagnostic role of radiolabeled nitrofurantoin.

Polymyxin B

Polymyxin B is used for resistant gram-negative infections. It binds to lipopolysaccharide to disrupt the outer membrane. Auletta et al. demonstrated that ^{99m}Tc-HYNIC–polymyxin B selectively targeted Gram-negative bacteria, including *P. aeruginosa*, *E. coli*, *Klebsiella pneumoniae*, and *Acinetobacter baumannii*, over the Gram-positive organisms *Enterococcus faecalis* and *Staphylococcus aureus*. Unfortunately this tracer showed high background organ uptake, especially in the lung [140]. Colistin, a close relative of polymyxin B, has a similar biodistribution, but has not been tested in infection models [141].

Oxazolidinones

Linezolid, the first licensed oxazolidinone, inhibits initiation of bacterial protein synthesis in gram-positive bacteria. A study using ^{131}I-linezolid in a *S. aureus* myositis model found a T/NT ratio of 11. However, it is unclear if the high T/NT ratio reflects strong infection localization or poor radiotracer penetration into normal muscle tissue as absolute uptake in uninfected muscle was not reported [143]. ^{18}F-linezolid was evaluated in a clinically relevant murine pneumonia model of *Mycobacterium tuberculosis*, but failed to distinguish infected from noninfected lung tissue. Despite the negative result, this study demonstrates that PET is useful for *in situ* measurement of antibiotic distribution across tissue compartments [144]. The second generation oxazolidinone, Tedizolid, has not been directly labeled [191] or evaluated for imaging.

Clindamycin

Lastly, clindamycin is a lincosamide antibiotic that inhibits bacterial 50S ribosome. ^{99m}Tc-clindamycin was unable to distinguish sterile inflammation from infection [132].

Antimycobacterial

Antimycobacterial cover drugs from multiple antibiotic classes. In this section we focus on those that uniquely target mycobacteria.

Isoniazid

Isoniazid, which is a prodrug that its metabolite inhibits the synthesis of mycolic acids, has been labeled with technetium-99m and fluorine-18, with ^{18}F-INH showing greater promise than ^{99m}Tc-INH for detecting *M. tuberculosis* (TB) infection in a murine model. Infected lung tissue demonstrated increased ^{18}F-INH uptake, but T/NT ratio at 40 minutes was a modest 1.67. Notably, a sterile inflammation model was not included and would have been an informative comparator. ^{11}C-INH, ^{99m}Tc-INH Solid Nanoparticles, and ^{99m}Tc-alginate-INH were all created for TB treatment and drug biodistribution purposes. ^{99m}T(CO)$_3$ and ^{99m}Tc-HYNIC-isoniazid did not demonstrate *in vitro* binding with *M. tuberculosis* so were not evaluated *in vivo* [192].

Other antimycobacterial

While radiolabeled INH has shown accumulation at sites of infection, other radiolabeled antituberculosis agents such as ^{11}C-rifampin, ^{18}F-fluoropyrazinamide, ^{18}F-fluoropretomanid, and ^{76}Br-bedaquiline conversely showed decreased tracer uptake in TB lesions [33, 154, 158, 159].

https://doi.org/10.3390/ijms27125313

Experience with radiolabeled antimycobacterial is summarized in Table 8.

Antivirals

Evaluation of radiolabeled antivirals has been limited compared to antibacterials. Antivirals tend to be more pathogen specific, targeting a certain virus or family of viruses, than antibacterials. The handful of radiolabeled antivirals studied to date have focused on HIV and Herpes viruses, and have been used for biodistribution or PK assessments.

Herpesviruses

Human pathogenic herpesviruses include herpes simplex virus (HSV), varicella-zoster virus (VZV), cytomegalovirus (CMV), and Epstein–Barr virus (EBV). Several nucleoside analog antiviral drugs are used to treat herpesvirus infections by inhibiting viral DNA synthesis, including acyclovir, ganciclovir, and penciclovir. These agents require activation by viral thymidine kinase to exert their antiviral effects. Radiolabeled derivatives of ganciclovir (^{18}F-FHBG) and penciclovir (^{18}F-FPCV) have been developed but were only evaluated for detecting transgenic tumors expressing HSV1-thymidine kinase in mice. Other radiolabeled nucleoside analogs, such as the uracil-based tracers FIAU and FEAU, have been used primarily for reporter gene imaging following target cell transfection [193, 194]. These applications are reviewed elsewhere [195, 196].

Human Immunodeficiency Virus

Since onset of the HIV epidemic in the 1980s, there has been remarkable progress in development of antiretrovirals targeting stages of the viral lifecycle. Integrase strand transfer inhibitors, which target the HIV enzyme integrase, are a cornerstone of modern antiretroviral therapy. One integrase strand transfer inhibitor, dolutegravir (DTG), was labeled with fluorine-18 [165]. This radiolabeled drugs have been used for PK studies, as opposed to imaging of viral activity. *In vivo* PET using ^{18}F-DTG confirmed elimination via the liver, kidneys, and gallbladder, as well as lack of CNS penetration. Bictegravir, a new generation of integrase inhibitor, has yet been labeled.

Nucleoside reverse transcriptase inhibitors, which interfere with the reverse transcription step of the HIV lifecycle, form the backbone of many current ART regimens. Within this class, only tenofovir was labeled with PET capable fluorine-18, but thus far applications have been limited to PK studies[166]. Ideally, radioligands will be developed for imaging of HIV reservoirs, which would allow non-invasive assessment of clinical interventions and cure strategies.

Influenza

One other antiviral has been labeled with carbon-11 isoseltamivir [167]. This is a selective neuraminidase inhibitor used to treat Influenza virus infection. Oseltamavir was radiolabeled as part of an attempt to understand why this drug with low CNS penetration is associated with increased neuropsychiatric adverse events in young people. Although it was hypothesized that immune activation would increase brain oseltamivir concentration, there was no difference in tracer uptake between baseline and after sterile immune activation in Japanese macaques.

Table 9 summarizes studies on radiolabeled antivirals.

Antifungals

As with antivirals, investigation of labeled antifungals has been limited. Unlike antivirals, nearly all antifungals have high serum protein binding (80 to 99%) [197], which may limit their utility as radiotracers for detection of infection. Both mentioned antifungals below have predominant renal metabolism.

https://doi.org/10.3390/ijms27125313

Fluconazole

Fluconazole inhibits 14-α lanosterol demethylase, which is crucial for synthesizing the fungal cell membrane component ergosterol. Fluconazole has been labeled with technetium-99m and fluorine-18 [168, 170]. Consistent with lack of activity against *Aspergillus* spp, ^{99m}Tc-fluconazole did not distinguish *A. fumigatus* infection from sterile inflammation, but the T/NT ratio for *C. albicans* infection was promising at 3.5. Further evaluation of biodistribution by Nogueira de Assis et al showed higher 2-hour blood pool uptake of ^{99m}Tc-fluconazole compared to lung and spleen; by 4 hours, background uptake had improved. Using AUC, the same group showed ^{99m}Tc-fluconazole had a T/NT of only 1.6 for *C. albicans* myositis. Other encapsulated versions of ^{99m}Tc-fluconazole were evaluated, but demonstrated worse background performance without appreciable T/NT gain [169]. ^{18}F-fluconazole had less background blood pool and organ uptake than ^{99m}Tc-fluconazole, but T/NT was suboptimal at 1.3 and a control sterile inflammation model was not assessed [170].

Amphotericin B

Amphotericin B (AmB) is a polyene antifungal that destabilizes the fungal membrane. Though ^{99m}Tc-AmB did not progress to *in vivo* assessment, the analog $^{99m}Tc(CO)_3$-AmB performed similarly to $^{99m}Tc(CO)_3$-caspofungin in terms of serum protein binding and T/NT ratios in biodistribution studies. Additional evaluation of $^{99m}Tc(CO)_3$-AmB by scintigraphy would be informative [174].

Experience with radiolabeled antifungals (Table 10) highlights the importance of *in vivo* evaluation in appropriate infection models.

References

1. Naqvi, S.A.R. ^{99m}Tc-labeled antibiotics for infection diagnosis: Mechanism, action, and progress. *Chem. Biol. Drug Des.* **2022**, *99*, 56–74. https://doi.org/10.1111/cbdd.13923.
2. Signore, A.; Bentivoglio, V.; Varani, M.; Lauri, C. Current Status of SPECT Radiopharmaceuticals for Specific Bacteria Imaging. *Semin. Nucl. Med.* **2023**, *53*, 142–151. https://doi.org/10.1053/j.semnuclmed.2022.12.001.
3. Ordonez, A.A.; Jain, S.K. Pathogen-Specific Bacterial Imaging in Nuclear Medicine. *Semin. Nucl. Med.* **2018**, *48*, 182–194. https://doi.org/10.1053/j.semnuclmed.2017.11.003.
4. Northrup, J.D.; Mach, R.H.; Sellmyer, M.A. Radiochemical Approaches to Imaging Bacterial Infections: Intracellular versus Extracellular Targets. *Int. J. Mol. Sci.* **2019**, *20*, 5808. https://doi.org/10.3390/ijms20225808.
5. Welling, M.M.; Hensbergen, A.W.; Bunschoten, A.; Velders, A.H.; Scheper, H.; Smits, W.K.; Roestenberg, M.; van Leeuwen, F.W.B. Fluorescent imaging of bacterial infections and recent advances made with multimodal radiopharmaceuticals. *Clin. Transl. Imaging* **2019**, *7*, 125–138. https://doi.org/10.1007/s40336-019-00322-7.
6. Liu, Sichen. 2026. "Radiolabeled Antibiotics for Infection Imaging: A Scoping Review." OSF. February 25. doi:10.17605/OSF.IO/KA2DN.
7. Harzing, A.W. *Publish or Perish*; Tarma Software Research Ltd: London, United Kingdom, 2007.
8. Team, T.E. *EndNote*, version 21; Clarivate: Philadelphia, PA, USA, 2013.
9. Ferro-Flores, G.; Avila-Rodríguez, M.A.; García-Pérez, F.O. Imaging of bacteria with radiolabeled ubiquicidin by SPECT and PET techniques. *Clin. Transl. Imaging* **2016**, *4*, 175–182. https://doi.org/10.1007/s40336-016-0178-7.
10. Sachdeva, A.; Mitra, J.B.; Mukherjee, A. Ubiquicidin derived peptides for infection imaging. *Nucl. Med. Biol.* **2025**, *146–147*, 109049. https://doi.org/10.1016/j.nucmedbio.2025.109049.
11. Vinjamuri, S.; Solanki, K.; Bomanji, J.; Siraj, Q.; Britton, K.; Hall, A.; O'Shaughnessy, E.; Das, S. Comparison of 99mTc infecton imaging with radiolabelled white-cell imaging in the evaluation of bacterial infection. *Lancet* **1996**, *347*, 233–235. https://doi.org/10.1016/S0140-6736(96)90407-9.
12. Britton, K.E.; Wareham, D.W.; Das, S.S.; Solanki, K.K.; Amaral, H.; Bhatnagar, A.; Katamihardja, A.H.S.; Malamitsi, J.; Moustafa, H.M.; Soroa, V.E.; et al. Imaging bacterial infection with ^{99m}Tc-ciprofloxacin (Infecton). *J. Clin. Pathol.* **2002**, *55*, 817–823. https://doi.org/10.1136/jcp.55.11.817.

13. Dumarey, N.; Blocklet, D.; Appelboom, T.; Tant, L.; Schoutens, A. Infecton is not specific for bacterial osteo-articular infective pathology. *Eur. J. Nucl. Med. Mol. Imaging* **2002**, *29*, 530–535. https://doi.org/10.1007/s00259-001-0749-2.
14. Larikka, M.J.; Ahonen, A.K.; NiemelÄ, O.; Puronto, O.; Junila, J.A.; HÄMÄLÄInen, M.M.; Britton, K.; SyrjÄLÄ, H.P. ^{99m}Tc-ciprofloxacin (Infecton) imaging in the diagnosis of knee prosthesis infections. *Nucl. Med. Commun.* **2002**, *23*, 167–170.
15. Lee, M.; Yoon, M.; Hwang, K.H.; Choe, W. Tc-99m Ciprofloxacin SPECT of Pulmonary Tuberculosis. *Nucl. Med. Mol. Imaging* **2010**, *44*, 116–122. https://doi.org/10.1007/s13139-010-0021-4.
16. Sarda, L.; Crémieux, A.-C.; Lebellec, Y.; Meulemans, A.; Lebtahi, R.; Hayem, G.; Génin, R.; Delahaye, N.; Huten, D.; Le Guludec, D. Inability of ^{99m}Tc-Ciprofloxacin Scintigraphy to Discriminate Between Septic and Sterile Osteoarticular Diseases. *J. Nucl. Med.* **2003**, *44*, 920–926.
17. Yapar, Z.; Kibar, M.; Yapar, F.A.; Toğrul, E.; Kayaselçuk, U.; Sarpel, Y. The efficacy of technetium-99m ciprofloxacin (Infecton) imaging in suspected orthopaedic infection: A comparison with sequential bone/gallium imaging. *Eur. J. Nucl. Med.* **2001**, *28*, 822–830. https://doi.org/10.1007/s002590100555.
18. Malamitsi, J.; Giamarellou, H.; Kanellakopoulou, K.; Dounis, E.; Grecka, V.; Christakopoulos, J.; Koratzanis, G.; Antoniadou, A.; Panoutsopoulos, G.; Batsakis, C.; et al. Infecton: A ^{99m}Tc-ciprofloxacin radiopharmaceutical for the detection of bone infection. *Clin. Microbiol. Infect.* **2003**, *9*, 101–109. https://doi.org/10.1046/j.1469-0691.2003.00506.x.
19. Langer, O.; Brunner, M.; Zeitlinger, M.; Ziegler, S.; Müller, U.; Dobrozemsky, G.; Lackner, E.; Joukhadar, C.; Mitterhauser, M.; Wadsak, W.; et al. *In vitro* and *in vivo* evaluation of [^{18}F]ciprofloxacin for the imaging of bacterial infections with PET. *Eur. J. Nucl. Med. Mol. Imaging* **2005**, *32*, 143–150. https://doi.org/10.1007/s00259-004-1646-2.
20. Brunner, M.; Langer, O.; Dobrozemsky, G.; Müller, U.; Zeitlinger, M.; Mitterhauser, M.; Wadsak, W.; Dudczak, R.; Kletter, K.; Müller, M. [^{18}F]Ciprofloxacin, a new positron emission tomography tracer for noninvasive assessment of the tissue distribution and pharmacokinetics of ciprofloxacin in humans. *Antimicrob. Agents Chemother.* **2004**, *48*, 3850–3857. https://doi.org/10.1128/aac.48.10.3850-3857.2004.
21. Fischman, A.J.; Livni, E.; Babich, J.W.; Alpert, N.M.; Bonab, A.; Chodosh, S.; McGovern, F.; Kamitsuka, P.; Liu, Y.Y.; Cleeland, R.; et al. Pharmacokinetics of [^{18}F]fleroxacin in patients with acute exacerbations of chronic bronchitis and complicated urinary tract infection studied by positron emission tomography. *Antimicrob. Agents Chemother.* **1996**, *40*, 659–664. https://doi.org/10.1128/aac.40.3.659.
22. Ammar, A.; Fatima, S.; Mir, K.; Butt, S.T.; Batool, S.; Saeed, M.A.; Marwat, N.; Ahmed, N. Utility of Tc-99m-labeled levofloxacin as an infection-imaging agent in musculoskeletal infections. *Pak. J. Nucl. Med.* **2020**, *10*, 13–19.
23. Kaul, A.; Hazari, P.P.; Rawat, H.; Singh, B.; Kalawat, T.C.; Sharma, S.; Babbar, A.K.; Mishra, A.K. Preliminary evaluation of technetium-99m-labeled ceftriaxone: Infection imaging agent for the clinical diagnosis of orthopedic infection. *Int. J. Infect. Dis.* **2013**, *17*, e263–e270. https://doi.org/10.1016/j.ijid.2012.10.011.
24. Ahmed, N.; Fatima, S.; Saeed, M.A.; Zia, M.; Irfan Ullah, J. ^{99m}Tc-Ceftizoxime: Synthesis, characterization and its use in diagnosis of diabetic foot osteomyelitis. *J. Med. Imaging Radiat. Oncol.* **2019**, *63*, 61–68. https://doi.org/10.1111/1754-9485.12841.
25. Lee, I.K.; Jacome, D.A.; Cho, J.K.; Tu, V.; Young, A.J.; Dominguez, T.; Northrup, J.D.; Etersque, J.M.; Lee, H.S.; Ruff, A.; et al. Imaging sensitive and drug-resistant bacterial infection with [^{11}C]-trimethoprim. *J. Clin. Investig.* **2023**, *132*, e156679. https://doi.org/10.1172/JCI156679.
26. Gordon, O.; Lee, D.E.; Liu, B.; Langevin, B.; Ordonez, A.A.; Dikeman, D.A.; Shafiq, B.; Thompson, J.M.; Sponseller, P.D.; Flavahan, K.; et al. Dynamic PET-facilitated modeling and high-dose rifampin regimens for Staphylococcus aureus orthopedic implant-associated infections. *Sci. Transl. Med.* **2021**, *13*, eabl6851. https://doi.org/10.1126/scitranslmed.abl6851.
27. Tucker, E.W.; Guglieri-Lopez, B.; Ordonez, A.A.; Ritchie, B.; Klunk, M.H.; Sharma, R.; Chang, Y.S.; Sanchez-Bautista, J.; Frey, S.; Lodge, M.A.; et al. Noninvasive ^{11}C-rifampin positron emission tomography reveals drug biodistribution in tuberculous meningitis. *Sci. Transl. Med.* **2018**, *10*, eaau0965. https://doi.org/10.1126/scitranslmed.aau0965.
28. Singh, N.; Bhatnagar, A. Clinical Evaluation of Efficacy of 99mTc-Ethambutol in Tubercular Lesion Imaging. *Tuberc. Res. Treat.* **2010**, *2010*, 618051. https://doi.org/10.1155/2010/618051.
29. Bhattacharya, B.; Damle, N.; Ranjan, P.; Arora, G.; Prakash, S.; Nischal, N.; Jorwal, P.; Kumar, A.; Tyagi, A.; Wig, N. ^{99m}Tc-Ethambutol Scintigraphy with Single-Photon Emission Computed Tomography/Computed Tomography in Lymph Node Tuberculosis: An Initial Experience. *Indian J. Nucl. Med.* **2022**, *37*, 323–328. https://doi.org/10.4103/ijnm.ijnm_207_21.
30. Kartamihardja, A.H.S.; Kurniawati, Y.; Gunawan, R. Diagnostic value of ^{99m}Tc-ethambutol scintigraphy in tuberculosis: Compared to microbiological and histopathological tests. *Ann. Nucl. Med.* **2018**, *32*, 60–68. https://doi.org/10.1007/s12149-017-1220-1.

31. Tewson, T.J.; Yang, D.; Wong, G.; Macy, D.; DeJesus, O.J.; Nickles, R.J.; Perlman, S.B.; Taylor, M.; Frank, P. The synthesis of fluorine-18 lomefloxacin and its preliminary use in human studies. *Nucl. Med. Biol.* **1996**, *23*, 767–772. https://doi.org/10.1016/0969-8051(96)00071-6.
32. Fischman, A.J.; Babich, J.W.; Bonab, A.A.; Alpert, N.M.; Vincent, J.; Callahan, R.J.; Correia, J.A.; Rubin, R.H. Pharmacokinetics of [^{18}F]trovafloxacin in healthy human subjects studied with positron emission tomography. *Antimicrob. Agents Chemother.* **1998**, *42*, 2048–2054. https://doi.org/10.1128/aac.42.8.2048.
33. Mota, F.; Ruiz-Bedoya, C.A.; Tucker, E.W.; Holt, D.P.; De Jesus, P.; Lodge, M.A.; Erice, C.; Chen, X.; Bahr, M.; Flavahan, K.; et al. Dynamic ^{18}F-Pretomanid PET imaging in animal models of TB meningitis and human studies. *Nat. Commun.* **2022**, *13*, 7974. https://doi.org/10.1038/s41467-022-35730-3.
34. Yaghoubi, S.; Barrio, J.R.; Dahlbom, M.; Iyer, M.; Namavari, M.; Satyamurthy, N.; Goldman, R.; Herschman, H.R.; Phelps, M.E.; Gambhir, S.S. Human Pharmacokinetic and Dosimetry Studies of [^{18}F]FHBG: A Reporter Probe for Imaging Herpes Simplex Virus Type-1 Thymidine Kinase Reporter Gene Expression. *J. Nucl. Med.* **2001**, *42*, 1225–1234.
35. Fischman, A.J.; Alpert, N.M.; Livni, E.; Ray, S.; Sinclair, I.; Callahan, R.J.; Correia, J.A.; Webb, D.; Strauss, H.W.; Rubin, R.H. Pharmacokinetics of 18F-labeled fluconazole in healthy human subjects by positron emission tomography. *Antimicrob. Agents Chemother.* **1993**, *37*, 1270–1277. https://doi.org/10.1128/aac.37.6.1270.
36. Solanki, K.K.; Bomanji, J.; Siraj, Q.; Small, M.; Britton, K.E. Tc-99m "Infecton"—A new class of radiopharmaceutical for imaging infection. *J. Nucl. Med.* **1993**, *34*, 119.
37. Zhang, H.; Jiang, N.-y.; Zhu, L. Experimental studies on imaging of infected site with ^{99m}Tc-Iabeled ciprofloxacin in mice. *Chin. Med. J.* **2009**, *122*, 1907–1909.
38. Sarda, L.; Saleh-Mghir, A.; Peker, C.; Meulemans, A.; Crémieux, A.-C.; Le Guludec, D. Evaluation of ^{99m}Tc-Ciprofloxacin Scintigraphy in a Rabbit Model of Staphylococcus aureus Prosthetic Joint Infection. *J. Nucl. Med.* **2002**, *43*, 239–245.
39. Halder, K.K.; Nayak, D.K.; Baishya, R.; Sarkar, B.R.; Sinha, S.; Ganguly, S.; Debnath, M.C. ^{99m}Tc-labeling of ciprofloxacin and nitrofuryl thiosemicarbazone using fac-[$^{99m}Tc(CO)_3(H_2O)_3$] core: Evaluation of their efficacy as infection imaging agents. *Metallomics* **2011**, *3*, 1041–1048. https://doi.org/10.1039/C1MT00068C.
40. Satpati, D.; Arjun, C.; Krishnamohan, R.; Samuel, G.; Banerjee, S. ^{68}Ga-labeled Ciprofloxacin Conjugates as Radiotracers for Targeting Bacterial Infection. *Chem. Biol. Drug Des.* **2016**, *87*, 680–686. https://doi.org/10.1111/cbdd.12701.
41. Koźmiński, P.; Gawęda, W.; Rzewuska, M.; Kopatys, A.; Kujda, S.; Dudek, M.K.; Halik, P.K.; Królicki, L.; Gniazdowska, E. Physicochemical and Biological Study of ^{99m}Tc and ^{68}Ga Radiolabelled Ciprofloxacin and Evaluation of [^{99m}Tc]Tc-CIP as Potential Diagnostic Radiopharmaceutical for Diabetic Foot Syndrome Imaging. *Tomography* **2021**, *7*, 829–842. https://doi.org/10.3390/tomography7040070.
42. Langer, O.; Mitterhauser, M.; Brunner, M.; Zeitlinger, M.; Wadsak, W.; Mayer, B.X.; Kletter, K.; Muller, M. Synthesis of fluorine-18-labeled ciprofloxacin for PET studies in humans. *Nucl. Med. Biol.* **2003**, *30*, 285–291. https://doi.org/10.1016/s0969-8051(02)00444-4.
43. Zijlstra, S.; Gunawan, J.; Freytag, C.; Burchert, W. Synthesis and evaluation of fluorine-18 labelled compounds for imaging of bacterial infections with pet. *Appl. Radiat. Isot.* **2006**, *64*, 802–807. https://doi.org/10.1016/j.apradiso.2006.02.095.
44. Goethals, P.; Volkaert, A. Preparation of N′4-[^{11}C]methyl-ciprofloxacin for positron emission tomography studies. *J. Label. Compd. Radiopharm.* **2002**, *45*, 213–216. https://doi.org/10.1002/jlcr.545.
45. Sachin, K.; Kim, E.M.; Cheong, S.J.; Jeong, H.J.; Lim, S.T.; Sohn, M.H.; Kim, D.W. Synthesis of N_4′-[^{18}F]fluoroalkylated ciprofloxacin as a potential bacterial infection imaging agent for PET study. *Bioconjug. Chem.* **2010**, *21*, 2282–2288. https://doi.org/10.1021/bc1002983.
46. Fang, S.; Jiang, Y.; Gan, Q.; Ruan, Q.; Xiao, D.; Zhang, J. Design, Preparation, and Evaluation of a Novel ^{99m}TcN Complex of Ciprofloxacin Xanthate as a Potential Bacterial Infection Imaging Agent. *Molecules* **2020**, *25*, 5837.
47. Papasavva, A.; Pirmettis, N.N.; Shegani, A.; Papadopoulou, E.; Kiritsis, C.; Georgoutsou-Spyridonos, M.; Mastellos, D.C.; Chiotellis, A.; Kyprianidou, P.; Pelecanou, M.; et al. Synthesis and Evaluation of $^{99m}Tc(CO)_3$ Complexes with Ciprofloxacin Dithiocarbamate for Infection Imaging. *Pharmaceutics* **2024**, *16*, 1210.
48. Rubin, R.H.; Livni, E.; Babich, J.; Alpert, N.M.; Liu, Y.-Y.; Tham, E.; Prosser, B.; Cleeland, R.; Callahan, R.J.; Correia, J.A.; et al. Pharmacokinetics of Fleroxacin as Studied by Positron Emission Tomography and [^{18}F]Fleroxacin. *Am. J. Med.* **1993**, *94*, 31S–37S. https://doi.org/10.1016/S0002-9343(20)31137-2.
49. Ibrahim, I.T.; Motaleb, M.A.; Attalah, K.M. Synthesis and biological distribution of ^{99m}Tc-norfloxacin complex, a novel agent for detecting sites of infection. *J. Radioanal. Nucl. Chem.* **2010**, *285*, 431–436. https://doi.org/10.1007/s10967-010-0607-4.

50. Nayak, D.; Baishya, R.; Halder, K.K.; Sen, T.; Sarkar, B.; Ganguly, S.; Das, M.K.; Debnath, M. Evaluation of ^{99m}Tc(i)-tricarbonyl complexes of fluoroquinolones for targeting bacterial infection. *Metallomics* **2012**, *4*, 1197–1208.
51. Zhang, S.; Zhang, W.; Wang, Y.; Jin, Z.; Wang, X.; Zhang, J.; Zhang, Y. Synthesis and biodistribution of a novel ^{99m}TcN complex of norfloxacin dithiocarbamate as a potential agent for bacterial infection imaging. *Bioconjug. Chem.* **2011**, *22*, 369–375. https://doi.org/10.1021/bc100357w.
52. Motaleb, M.A. Radiochemical and biological characteristics of ^{99m}Tc-difloxacin and ^{99m}Tc-pefloxacin for detecting sites of infection. *J. Label. Compd. Radiopharm. Off. J. Int. Isot. Soc.* **2010**, *53*, 104–109.
53. El-Ghany, E.A.; El-Kolaly, M.T.; Amine, A.M.; El-Sayed, A.S.; Abdel-Gelil, F. Synthesis of ^{99m}Tc-pefloxacin: A new targeting agent for infectious foci. *J. Radioanal. Nucl. Chem.* **2005**, *266*, 131–139. https://doi.org/10.1007/s10967-005-0881-8.
54. Motaleb, M.A. Preparation and biodistribution of ^{99m}Tc-lomefloxacin and ^{99m}Tc-ofloxacin complexes. *J. Radioanal. Nucl. Chem.* **2007**, *272*, 95–99. https://doi.org/10.1007/s10967-006-6786-3.
55. Motaleb, M.A.; Ayoub, S.M. Preparation, quality control, and biodistribution of ^{99m}Tc-rufloxacin complex as a model for detecting sites of infection. *Radiochemistry* **2013**, *55*, 610–614. https://doi.org/10.1134/S1066362213060088.
56. Shah, S.Q.; Khan, M.R. Radiocharacterization of the ^{99m}Tc–rufloxacin complex and biological evaluation in Staphylococcus aureus infected rat model. *J. Radioanal. Nucl. Chem.* **2011**, *288*, 373–378. https://doi.org/10.1007/s10967-010-0923-8.
57. Shahzad, S.; Qadir, M.A.; Rasheed, R.; Ahmad, A.; Shafiq, M.I.; Ahmed, M.; Noreen, S.; Ali, A.; Shahzadi, S.K.; Javed, M. A new method for synthesis of ^{99m}Tc-enorfloxacin: An infection imaging agent. *Lat. Am. J. Pharm.* **2016**, *35*, 259–264.
58. Siaens, R.H.; Rennen, H.J.; Boerman, O.C.; Dierckx, R.; Slegers, G. Synthesis and comparison of ^{99m}Tc-enrofloxacin and ^{99m}Tc-ciprofloxacin. *J. Nucl. Med.* **2004**, *45*, 2088–2094.
59. Naqvi, S.; Ishfaq, M.; Khan, Z.; Nagra, S.; Bukhari, I.; Hussain, A.; Mahmood, N.; Shahzad, S.; Haq, A.; Bokhari, T. ^{99m}Tc labeled levofloxacin as an infection imaging agent: A novel method for labeling levofloxacin using cysteine·HCl as co-ligand and *in vivo* study. *Turk. J. Chem.* **2012**, *36*, 267–277. https://doi.org/10.3906/kim-1107-69.
60. Shahzad, S.; Qadir, M.A.; Rasheed, R.; Anwar, S.; Ahmed, M. *In vivo* studies ^{99m}Tc-levofloxacin freeze dried kits in Salmonella typhi, Pseudoman aeruginosa, and Escherichia coli. *Lat. Am. J. Pharm.* **2015**, *34*, 760–765.
61. Shah, S.Q.; Khan, M.R. Radiosynthesis and biodistribution of ^{99m}Tc-tricarbonyl complex of temafloxacin dithiocarbamate: A potential Streptococci pneumoniae infection radiotracer. *J. Radioanal. Nucl. Chem.* **2011**, *288*, 411–416. https://doi.org/10.1007/s10967-010-0936-3.
62. Shah, S.Q.; Khan, M.R. Synthesis of $^{99m}Tc(CO)_3$-Pazufloxacin Dithiocarbamate Complex and Biodistribution in Experimentally Induced Infection in Female Nude Mice. *Synth. React. Inorg. Met.-Org. Nano-Met. Chem.* **2012**, *42*, 190–195. https://doi.org/10.1080/15533174.2011.609516.
63. Shah, S.Q.; Khan, M.R. Synthesis of ^{99m}Tc-Pazufloxacin dithiocarbamate complex and biological evaluation in Wister rats artificially infected with. *J. Radioanal. Nucl. Chem.* **2011**, *288*, 511–516. https://doi.org/10.1007/s10967-010-0954-1.
64. Moustapha, M.E.; Motaleb, M.A.; Shweeta, H.; Farouk, M. Synthesis and biological evaluation of technetium-sarafloxacin complex for infection imaging. *J. Radioanal. Nucl. Chem.* **2016**, *307*, 699–705. https://doi.org/10.1007/s10967-015-4188-0.
65. Shah, S.Q.; Khan, M.R. $^{99m}Tc(CO)_3$-tosufloxacin dithiocarbamate complexation and radiobiological evaluation in male Wister rat model. *J. Radioanal. Nucl. Chem.* **2011**, *288*, 485–490. https://doi.org/10.1007/s10967-010-0943-4.
66. Singh, A.K.; Verma, J.; Bhatnagar, A.; Ali, A. Tc-99m labeled Sparfloxacin: A specific infection imaging agent. *World J. Nucl. Med.* **2003**, *2*, 103–109.
67. Eid Moustapha, M.; Shweeta, H.A.; Motaleb, M.A. Technetium-labeled danofloxacin complex as a model for infection imaging. *Arab. J. Chem.* **2016**, *9*, S1928–S1934. https://doi.org/10.1016/j.arabjc.2014.10.017.
68. Shah, S.Q.; Khan, M.R. Radiolabeling of gemifloxacin with technetium-99m and biological evaluation in artificially Streptococcus pneumoniae infected rats. *J. Radioanal. Nucl. Chem.* **2011**, *288*, 307–312. https://doi.org/10.1007/s10967-010-0916-7.
69. Shahzad, S.; Qadir, M.A.; Rasheed, R.; Ahmed, M. Synthesis of ^{99m}Tc-gemifloxacin freeze dried kits and their biodistribution in biodistribution in Salmonella typhi, Pseudomonas aeruginosa and Klebsiella Pneumonia. *Arab. J. Chem.* **2019**, *12*, 664–670. https://doi.org/10.1016/j.arabjc.2015.10.002.
70. Khoramrouz, S.J.; Erfani, M.; Athari Allaf, M. Technetium-99m Tricarbonyl Labeled a Broad-spectrum Quinolone as a Specific Imaging Agent in Infection Diseases. *Iran. J. Pharm. Res.* **2017**, *16*, 611–618.
71. Motaleb, M.A.; El-Kolaly, M.T.; Ibrahim, A.B.; Abd El-Bary, A. Study on the preparation and biological evaluation of 99mTc-gatifloxacin and 99mc-cefepime complexes. *J. Radioanal. Nucl. Chem.* **2011**, *289*, 57–65. https://doi.org/10.1007/s10967-011-1058-2.

72. Shah, S.Q.; Khan, A.U.; Khan, M.R. Radiosynthesis and biological evaluation of ^{99m}TcN-sitafloxacin dithiocarbamate as a potential radiotracer for Staphylococcus aureus infection. *J. Radioanal. Nucl. Chem.* **2011**, *287*, 827–832. https://doi.org/10.1007/s10967-010-0833-9.
73. Shah, S.Q.; Khan, A.U.; Khan, M.R. Radiosynthesis and biological evolution of $^{99m}Tc(CO)_3$-sitafloxacin dithiocarbamate complex: A promising Staphylococcus aureus infection radiotracer. *J. Radioanal. Nucl. Chem.* **2011**, *288*, 131–136. https://doi.org/10.1007/s10967-010-0880-2.
74. Chattopadhyay, S.; Saha Das, S.; Chandra, S.; De, K.; Mishra, M.; Ranjan Sarkar, B.; Sinha, S.; Ganguly, S. Synthesis and evaluation of ^{99m}Tc-moxifloxacin, a potential infection specific imaging agent. *Appl. Radiat. Isot.* **2010**, *68*, 314–316. https://doi.org/10.1016/j.apradiso.2009.10.030.
75. Shah, S.Q.; Khan, M.R. Radiosynthesis and biological evaluation of the ^{99m}Tc-tricarbonyl moxifloxacin dithiocarbamate complex as a potential Staphylococcus aureus infection radiotracer. *Appl. Radiat. Isot.* **2011**, *69*, 686–690. https://doi.org/10.1016/j.apradiso.2011.01.003.
76. Fischman, A.J.; Babich, J.W.; Alpert, N.M.; Vincent, J.; Wilkinson, R.A.; Callahan, R.J.; Correia, J.A.; Rubin, R.H. Pharmacokinetics of ^{18}F-labeled trovafloxacin in normal and Escherichia coli-infected rats and rabbits studied with positron emission tomography. *Clin. Microbiol. Infect.* **1997**, *3*, 63–72. https://doi.org/10.1111/j.1469-0691.1997.tb00253.x.
77. Shah, S.Q.; Khan, M.R. Radiosynthesis and biodistribution of the ^{99m}Tc-trovafloxacin complex as a potential methicillin resistant Staphylococcus aureus infection radiotracer. *J. Radioanal. Nucl. Chem.* **2011**, *288*, 525–530. https://doi.org/10.1007/s10967-011-0991-4.
78. Shah, S.Q.; Khan, M.R. Radiocomplexation and biological characterization of the ^{99m}TcN-trovafloxacin dithiocarbamate: A novel methicillin-resistant *Staphylococcus aureus* infection imaging agent. *J. Radioanal. Nucl. Chem.* **2011**, *288*, 215–220. https://doi.org/10.1007/s10967-010-0903-z.
79. Shah, S.Q.; Khan, M.R. Synthesis of the $^{99m}Tc(CO)_3$-trovafloxacin dithiocarbamate complex and biological characterization in artificially methicillin-resistant Staphylococcus aureus infected rats model. *J. Radioanal. Nucl. Chem.* **2011**, *288*, 297–302. https://doi.org/10.1007/s10967-010-0914-9.
80. Shah, S.Q.; Khan, A.U.; Khan, M.R. Synthesis, biological evaluation and biodistribution of the ^{99m}Tc-Garenoxacin complex in artificially infected rats. *J. Radioanal. Nucl. Chem.* **2011**, *288*, 207–213. https://doi.org/10.1007/s10967-010-0896-7.
81. Shah, S.Q.; Khan, A.U.; Khan, M.R. Radiosynthesis and biodistribution of ^{99m}TcN-Garenoxacin dithiocarbamate complex a potential infection imaging agent. *J. Radioanal. Nucl. Chem.* **2011**, *288*, 59–64. https://doi.org/10.1007/s10967-010-0871-3.
82. Shah, S.Q.; Khan, A.U.; Khan, M.R. $^{99m}Tc(CO)_3$–Garenoxacin dithiocarbamate synthesis and biological evolution in rats infected with multiresistant Staphylococcus aureus and penicillin-resistant Streptococci. *J. Radioanal. Nucl. Chem.* **2011**, *288*, 171–176. https://doi.org/10.1007/s10967-010-0892-y.
83. Shah, S.Q.; Khan, M.R.; Ali, S.M. Radiosynthesis of $^{99m}Tc(CO)_3$-Clinafloxacin Dithiocarbamate and Its Biological Evaluation as a Potential Staphylococcus aureus Infection Radiotracer. *Nucl. Med. Mol. Imaging* **2011**, *45*, 248–254. https://doi.org/10.1007/s13139-011-0106-8.
84. Shah, S.Q.; Khan, M.R. Synthesis of ^{99m}TcN-clinafloxacin Dithiocarbamate Complex and Comparative Radiobiological Evaluation in Staphylococcus aureus Infected Mice. *World J. Nucl. Med.* **2014**, *13*, 154–158. https://doi.org/10.4103/1450-1147.144813.
85. Shah, S.; Khan, M. Synthesis of techentium-99m labeled clinafloxacin (^{99m}Tc–CNN) complex and biological evaluation as a potential Staphylococcus aureus infection imaging agent. *J. Radioanal. Nucl. Chem.* **2011**, *288*, 423–428.
86. Shah, S.Q.; Khan, A.U.; Khan, M.R. ^{99m}Tc-prulifloxacin in artificially infected animals. Radiosynthesis and biological evaluation. *Nuklearmedizin* **2011**, *50*, 134–140. https://doi.org/10.3413/Nukmed-0334-10-07.
87. El-Kawy, O.A.; Farah, K. Radiocomplexation and biological evaluation of nemonoxacin in mice infected with multiresistant Staphylococcus aureus and penicillin-resistant Streptococci. *J. Radioanal. Nucl. Chem.* **2015**, *306*, 123–130. https://doi.org/10.1007/s10967-015-4069-6.
88. Shahzad, M.A.; Naqvi, S.A.R.; Rasheed, R.; Yameen, M.; Anjum, F.; Ahmed, M.T.; Hussain, Z.; Gillani, S.J.H. Radiolabeling of benzylpenicillin with lutetium-177: Quality control and biodistribution study to develop theranostic infection imaging agent. *Pak. J. Pharm. Sci.* **2017**, *30*, 2349–2354.
89. Shahzadi, S.K.; Qadir, M.A.; Shabnam, S.; Javed, M. ^{99m}Tc-amoxicillin: A novel radiopharmaceutical for infection imaging. *Arab. J. Chem.* **2019**, *12*, 2533–2539. https://doi.org/10.1016/j.arabjc.2015.04.003.
90. Durkan, K.; Tuncel, A.; Yurt, F. *In vitro* evaluation of ^{99m}Tc-sultamicillin for infection imaging. *Biopharm. Drug Dispos.* **2021**, *42*, 285–293. https://doi.org/10.1002/bdd.2281.

91. El-Tawoosy, M. Preparation and biological distribution of ^{99m}Tc-cefazolin complex, a novel agent for detecting sites of infection. *J. Radioanal. Nucl. Chem.* **2013**, *298*, 1215–1220. https://doi.org/10.1007/s10967-013-2593-9.
92. Sanad, M.H.; Eh, B. Performance characteristics of biodistribution of ^{99m}Tc-cefprozil for *in vivo* infection imaging. *J. Anal. Sci. Technol.* **2014**, *5*, 32. https://doi.org/10.1186/S40543-014-0032-3.
93. Chattopadhyay, S.; Ghosh, M.; Sett, S.; Das, M.K.; Chandra, S.; De, K.; Mishra, M.; Sinha, S.; Ranjan Sarkar, B.; Ganguly, S. Preparation and evaluation of ^{99m}Tc-cefuroxime, a potential infection specific imaging agent: A reliable thin layer chromatographic system to delineate impurities from the ^{99m}Tc-antibiotic. *Appl. Radiat. Isot.* **2012**, *70*, 2384–2387. https://doi.org/10.1016/j.apradiso.2012.06.007.
94. Yurt Lambrecht, F.; Durkan, K.; Unak, P. Preparation, quality control and stability of ^{99m}Tc-cefuroxime axetil. *J. Radioanal. Nucl. Chem.* **2008**, *275*, 161–164. https://doi.org/10.1007/s10967-007-6999-0.
95. Mostafa, M.; Motaleb, M.A.; Sakr, T.M. Labeling of ceftriaxone for infective inflammation imaging using 99mTc eluted from 99Mo/99mTc generator based on zirconium molybdate. *Appl. Radiat. Isot.* **2010**, *68*, 1959–1963. https://doi.org/10.1016/j.apradiso.2010.04.031.
96. Sohaib, M.; Khurshid, Z.; Roohi, S. Labelling of ceftriaxone with 99mTc and its bio-evaluation as an infection imaging agent. *J. Label. Compd. Radiopharm.* **2014**, *57*, 652–657. https://doi.org/10.1002/jlcr.3235.
97. Fazli, A.; Salouti, M.; Mazidi, M. ^{99m}Tc-ceftriaxone, as a targeting radiopharmaceutical for scintigraphic imaging of infectious foci due to Staphylococcus aureus in mouse model. *J. Radioanal. Nucl. Chem.* **2013**, *298*, 1227–1233. https://doi.org/10.1007/s10967-013-2523-x.
98. Teixeira, L.E.M.; Soares, G.G.; Teixeira, H.C.; Takenaka, I.K.T.M.; Diniz, S.O.F.; de Andrade, M.A.P.; Cardoso, V.N.; de Araújo, I.D. Efficacy of ^{99m}Tc-Labeled Ceftizoxime in the Diagnosis of Subclinical Infections Associated with Titanium Implants in Rats. *Surg. Infect.* **2015**, *16*, 352–357. https://doi.org/10.1089/sur.2014.186.
99. Costa, P.H.; Diniz, S.O.; Cardoso, V.N.; Tarabal, B.; Takenaka, I.; Braga, O.; Vidigal, P.V.; Gelape, C.L.; Araujo, I.D. Scintigraphic imaging with technetium-99M-labelled ceftizoxime is a reliable technique for the diagnosis of deep sternal wound infection in rats. *Acta Cir. Bras.* **2015**, *30*, 632–638. https://doi.org/10.1590/S0102-865020150090000008.
100. Mirshojaei, S.F.; Gandomkar, M.; Najafi, R.; Sadat Ebrahimi, S.E.; Babaei, M.H.; Shafiei, A.; Talebi, M.H. Radio labeling, quality control and biodistribution of 99mTc-cefotaxime as an infection imaging agent. *J. Radioanal. Nucl. Chem.* **2011**, *287*, 21–25. https://doi.org/10.1007/s10967-010-0826-8.
101. Ilem-Ozdemir, D.; Asikoglu, M.; Ozkilic, H.; Yilmaz, F.; Hosgor-Limoncu, M.; Ayhan, S. Gamma scintigraphy and biodistribution of ^{99m}Tc-cefotaxime sodium in preclinical models of bacterial infection and sterile inflammation. *J. Label. Compd. Radiopharm.* **2016**, *59*, 109–116. https://doi.org/10.1002/jlcr.3374.
102. Mirshojaei, S.F.; Erfani, M.; Shafiei, M. Evaluation of ^{99m}Tc-ceftazidime as bacterial infection imaging agent. *J. Radioanal. Nucl. Chem.* **2013**, *298*, 19–24. https://doi.org/10.1007/s10967-013-2418-x.
103. Motaleb, M.A. Preparation of ^{99m}Tc-cefoperazone complex, a novel agent for detecting sites of infection. *J. Radioanal. Nucl. Chem.* **2007**, *272*, 167–171. https://doi.org/10.1007/s10967-006-6754-y.
104. Koźmiński, P.; Rzewuska, M.; Piądłowska, A.; Halik, P.; Gniazdowska, E. Synthesis, physicochemical and *in vitro* biological evaluation of ^{99m}Tc-cefepime radioconjugates, and development of DTPA-cefepime single vial kit formulation for labelling with technetium-99m. *J. Radioanal. Nucl. Chem.* **2022**, *331*, 2883–2894. https://doi.org/10.1007/s10967-022-08363-5.
105. Sakr, T.M.; Motaleb, M.A.; Ibrahim, I.T. ^{99m}Tc–meropenem as a potential SPECT imaging probe for tumor hypoxia. *J. Radioanal. Nucl. Chem.* **2012**, *292*, 705–710. https://doi.org/10.1007/s10967-011-1481-4.
106. Naqvi, S.A.R.; Jabbar, T.; Alharbi, M.A.; Noureen, A.; Alharbi, N.K.; Sherazi, T.A.; Shahzadi, A.; Ahmed, A.E.; Afzal, M.S.; Imran, M.B. Radiosynthesis, quality control, biodistribution, and infection-imaging study of a new ^{99m}Tc-labeled ertapenem radiopharmaceutical. *Front. Chem.* **2022**, *10*, 1020387. https://doi.org/10.3389/fchem.2022.1020387.
107. Ozker, K.; Urgancioğlu, I. ^{99m}Tc-gentamicin: Chemical and biological evaluation. *Eur. J. Nucl. Med.* **1981**, *6*, 173–176. https://doi.org/10.1007/bf00253169.
108. Amina Watson, R.A.; Landon, J.; Edwards, C.R.W.; Shaw, E.J. Improved ^{125}I-ligands for gentamicin radioimmunoassay. *J. Antimicrob. Chemother.* **1979**, *5*, 673–680. https://doi.org/10.1093/jac/5.6.673.
109. Dhanani, J.A.; Goodman, S.; Ahern, B.; Cohen, J.; Fraser, J.F.; Barnett, A.; Diab, S.; Bhatt, M.; Roberts, J.A. Comparative lung distribution of radiolabeled tobramycin between nebulized and intravenous administration in a mechanically-ventilated ovine model, an observational study. *Int. J. Antimicrob. Agents* **2021**, *57*, 106232. https://doi.org/10.1016/j.ijantimicag.2020.106232.

110. Van't Veen, A.; Gommers, D.; Verbrugge, S.J.; Wollmer, P.; Mouton, J.W.; Kooij, P.P.; Lachmann, B. Lung clearance of intratracheally instilled ^{99m}Tc-tobramycin using pulmonary surfactant as vehicle. *Br. J. Pharmacol.* **1999**, *126*, 1091–1096. https://doi.org/10.1038/sj.bjp.0702405.
111. Roohi, S.; Mushtaq, A.; Jehangir, M.; Malik, S.A. Synthesis, quality control and biodistribution of ^{99m}Tc-Kanamycin. *J. Radioanal. Nucl. Chem.* **2006**, *267*, 561–566, https://doi.org/10.1007/s10967-006-0087-8.
112. Widyasari, E.; Halimah, I.; Sugiharti, R.J.; Sriyani, M.; Daruwati, I.; Iswahyudi, I.; Isabela, E.; Nuraeni, W. Biological Evaluation of ^{99m}Tc-Kanamycin for Infection Imaging. *Indones. J. Phys. Nucl. Appl.* **2017**, *2*, 34. https://doi.org/10.24246/ijpna.v2i1.34-41.
113. Akbar, M.U.; Bokhari, T.H.; Khalid, M.; Ahmad, M.R.; Roohi, S.; Hina, S.; Mehmood, S.; Sohaib, M.; Jabbar, T. Radiolabeling, quality control, and biological characterization of ^{177}Lu-labeled kanamycin. *Chem. Biol. Drug Des.* **2017**, *90*, 425–431. https://doi.org/10.1111/cbdd.12960.
114. El-Kawy, O.A.; Abdelaziz, G.; Abdel-Razek, A.S. Radiolabeling, characterization, and preclinical evaluation of plazomicin: A potential tracer for bacterial infection. *Chem. Biol. Drug Des.* **2022**, *99*, 688–702. https://doi.org/10.1111/cbdd.14007.
115. Ercan, M.T.; Aras, T.; Unsal, I.S. Evaluation of ^{99m}Tc-erythromycin and ^{99m}Tc-streptomycin sulphate for the visualization of inflammatory lesions. *Int. J. Radiat. Appl. Instrum. B* **1992**, *19*, 803–806. https://doi.org/10.1016/0883-2897(92)90143-m.
116. Sanad, M.H. Labeling and biological evaluation of ^{99m}Tc-azithromycin for infective inflammation diagnosis. *Radiochemistry* **2013**, *55*, 539–544. https://doi.org/10.1134/S1066362213050159.
117. Abdel-Ghaney, I.Y.; Sanad, M.H. Synthesis of ^{99m}Tc-Erythromycin Complex as a Model for Infection Sites Imaging. *Radiochemistry* **2013**, *55*, 418–422. https://doi.org/10.1134/S1066362213040139.
118. Borai, E.H.; Sanad, M.H.; Fouzy, A.S.M. Optimized chromatographic separation and biological evaluation of ^{99m}Tc-clarithromycin for infective inflammation diagnosis. *Radiochemistry* **2016**, *58*, 84–91. https://doi.org/10.1134/S1066362216010136.
119. Rizvi, S.F.A.; Tariq, S.; Mehdi, M.; Hassan, A.J. Synthesis of ^{99m}Tc-roxithromycin: A novel diagnostic agent to discriminate between septic and aseptic inflammation. *Chem. Biol. Drug Des.* **2019**, *93*, 1166–1174. https://doi.org/10.1111/cbdd.13412.
120. İlem-Özdemir, D.; Asikoglu, M.; Ozkilic, H.; Yilmaz, F.; Hosgor-Limoncu, M.; Ayhan, S. ^{99m}Tc-Doxycycline hyclate: A new radiolabeled antibiotic for bacterial infection imaging. *J. Label. Compd. Radiopharm.* **2014**, *57*, 36–41. https://doi.org/10.1002/jlcr.3135.
121. Rizvi, S.F.A.; Jabbar, T.; Shahid, W.; Sanad, M.H.; Zhang, H. Facile One-Pot Strategy for Radiosynthesis of ^{99m}Tc-Doxycycline to Diagnose Staphylococcus aureus in Infectious Animal Models. *Appl. Biochem. Biotechnol.* **2022**, *194*, 2672–2683. https://doi.org/10.1007/s12010-022-03856-1.
122. Milanović, Z.; Janković, D.; Vranješ-Đurić, S.; Radović, M.; Prijović, Ž.; Zavišić, G.; Perić, M.; Stanković, D.; Mirković, M. ^{177}Lu-doxycycline as potential radiopharmaceutical: Electrochemical characterization, radiolabeling, and biodistribution in tumor-bearing mice. *Int. J. Radiat. Biol.* **2021**, *97*, 1687–1695. https://doi.org/10.1080/09553002.2021.1976864.
123. Dewanjee, M.K.; Fliegel, C.; Treves, S.; Davis, M.A. ^{99m}Tc-tetracyclines: Preparation and biological evaluation. *J. Nucl. Med.* **1974**, *15*, 176–182.
124. Philip, L.H.; Taylor, A.; Chauncey, D.M.; Schelbert, H. Comparison of ^{131}I-tetracycline and ^{67}Ga-citrate as abscess localizing agents. *Nuklearmedizin* **1977**, *16*, 76–78. https://doi.org/10.1055/s-0037-1620610.
125. Saleem, S.M.; Jabbar, T.; Imran, M.B.; Noureen, A.; Sherazi, T.A.; Afzal, M.S.; Rab Nawaz, H.Z.; Ramadan, M.F.; Alkahtani, A.M.; Alsuwat, M.A.; et al. Radiosynthesis and Preclinical Evaluation of [^{99m}Tc]Tc-Tigecycline Radiopharmaceutical to Diagnose Bacterial Infections. *Pharmaceuticals* **2024**, *17*, 1283. https://doi.org/10.3390/ph17101283.
126. Essouissi, I.; Ghali, W.; Saied, N.M.; Saidi, M. Synthesis and evaluation of ^{99m}Tc-N-sulfanilamide ferrocene carboxamide as bacterial infections detector. *Nucl. Med. Biol.* **2010**, *37*, 821–829. https://doi.org/10.1016/j.nucmedbio.2010.04.139.
127. Ahmed, M.T.; Naqvi, S.A.R.; Rasheed, R.; Zahoor, A.F.; Usman, M.; Hussain, Z. Technetium-99m-Labeled Sulfadiazine: A Targeting Radiopharmaceutical for Scintigraphic Imaging of Infectious Foci Due To Escherichia coli in Mouse and Rabbit Models. *Appl. Biochem. Biotechnol.* **2017**, *183*, 374–384. https://doi.org/10.1007/s12010-017-2451-2.
128. Essouissi, I.; Darghouth, F.; Saied, N.M.; Saidi, M.; Kanoun, A.; Saidi, M. Radiolabeling, Quality Control, and Biodistribution of 99mTc-Sulfadiazine as an Infection Imaging Agent. *Radiochemistry* **2015**, *57*, 307–311. https://doi.org/10.1134/S106636221503011x.
129. Ahmed, M.T.; Yameen, M.; Munir, B.; Asim, S.; Usman, M.; Naqvi, S.A.R.; Gillani, J.A.H.; Rasheed, R.; Shahzad, M.A. Evaluation of ^{99m}Tc-sulfadiazine as Bacillus microorganisms infection imaging agent using animal model. *Pak. J. Pharm. Sci.* **2018**, *31*, 1495–1499.
130. Liu, F.; Zhao, B.; Xia, X.; Yan, J.; Yu, F.; Yan, G.; Hu, J.; Chen, S.; Wang, Y.; Liu, H.; et al. Al^{18}F labeled sulfonamide-conjugated positron emission tomography tracer *in vivo* tumor-targeted imaging. *J. Cell. Biochem.* **2019**, *120*, 17006–17014. https://doi.org/10.1002/jcb.28961.

131. Amin, A.M.; Ibrahim, I.T.; Attallah, K.M.Z.; Ali, S. ^{99m}Tc-sulfadimidine as a potential radioligand for differentiation between septic and aseptic inflammations. *Radiochemistry* **2014**, *56*, 72–75.
132. Hina, S.; Rajoka, M.I.; Roohi, S.; Haque, A.; Qasim, M. Preparation, Biodistribution, and Scintigraphic Evaluation of ^{99m}Tc-Clindamycin: An Infection Imaging Agent. *Appl. Biochem. Biotechnol.* **2014**, *174*, 1420–1433. https://doi.org/10.1007/s12010-014-1075-z.
133. Bokhari, T.H.; Rizvi, S.F.A.; Roohi, S.; Hina, S.; Mushtaq, A.; Khalid, M.; Iqbal, M. Preparation, biodistribution and scintigraphic evaluation of ^{99m}Tc-lincomycin. *Pak. J. Pharm. Sci.* **2015**, *28*, 1965–70.
134. Sellmyer, M.A.; Lee, I.; Hou, C.; Lieberman, B.P.; Zeng, C.; Mankoff, D.A.; Mach, R.H. Quantitative PET Reporter Gene Imaging with [^{11}C]Trimethoprim. *Mol. Ther.* **2017**, *25*, 120–126. https://doi.org/10.1016/j.ymthe.2016.10.018.
135. Sellmyer, M.A.; Lee, I.; Hou, C.; Weng, C.C.; Li, S.; Lieberman, B.P.; Zeng, C.; Mankoff, D.A.; Mach, R.H. Bacterial infection imaging with [^{18}F]fluoropropyl-trimethoprim. *Proc. Natl. Acad. Sci. USA* **2017**, *114*, 8372–8377. https://doi.org/10.1073/pnas.1703109114.
136. Iqbal, A.; Naqvi, S.A.R.; Rasheed, R.; Mansha, A.; Ahmad, M.; Zahoor, A.F. Radiosynthesis and Biodistribution of ^{99m}Tc-Metronidazole as an *Escherichia coli* Infection Imaging Radiopharmaceutical. *Appl. Biochem. Biotechnol.* **2018**, *185*, 127–139. https://doi.org/10.1007/s12010-017-2641-y.
137. Kong, D.J.; Lu, J.; Ye, S.Z.; Wang, X.B. Synthesis and biological evaluation of a novel asymmetrical ^{99m}Tc-nitrido complex of metronidazole derivative. *J. Label. Compd. Radiopharm.* **2007**, *50*, 1137–1142. https://doi.org/10.1002/jlcr.1292.
138. Shah, S.Q.; Khan, A.U.; Khan, M.R. Radiosynthesis of ^{99m}Tc-nitrofurantoin a novel radiotracer for *in vivo* imaging of Escherichia coli infection. *J. Radioanal. Nucl. Chem.* **2011**, *287*, 417–422. https://doi.org/10.1007/s10967-010-0697-z.
139. El-Azony, K.M.; El-Mohty, A.A.; Seddik, U.; Khater, S.I. Radioiodination and bioevaluation of nitrofurantoin for urinary tract imaging. *J. Label. Compd. Radiopharm.* **2012**, *55*, 315–319. https://doi.org/10.1002/jlcr.2942.
140. Auletta, S.; Galli, F.; Varani, M.; Campagna, G.; Conserva, M.; Martinelli, D.; Santino, I.; Signore, A. *In vitro* and *In vivo* Evaluation of ^{99m}Tc-Polymyxin B for Specific Targeting of Gram-Bacteria. *Biomolecules* **2021**, *11*, 232. https://doi.org/10.3390/biom11020232.
141. Kumar, P.; Shanbhag, N.C.; Chaudhari, P.; Mohanty, B.; Thakur, R.; Sasidharan, G.M. Radiolabeling and preclinical evaluation of technetium-99m labeled colistin. *Appl. Radiat. Isot.* **2024**, *214*, 111524. https://doi.org/10.1016/j.apradiso.2024.111524.
142. Karpuz, M.; Özgenç, E.; Gündoğdu, E.; Burak, Z. Pre-study on radiolabeling of colistin with Lutetium-177 to develop theranostic infection agent. *J. Res. Pharm.* **2022**, *26*, 397–407.
143. Yurt Lambrecht, F.; Yilmaz, O.; Durkan, K.; Unak, P.; Bayrak, E. Preparation and biodistribution of [^{131}I]linezolid in animal model infection and inflammation. *J. Radioanal. Nucl. Chem.* **2009**, *281*, 415–419. https://doi.org/10.1007/s10967-009-0006-x.
144. Mota, F.; Jadhav, R.; Ruiz-Bedoya, C.A.; Ordonez, A.A.; Klunk, M.H.; Freundlich, J.S.; Jain, S.K. Radiosynthesis and Biodistribution of ^{18}F-Linezolid in Mycobacterium tuberculosis-Infected Mice Using Positron Emission Tomography. *ACS Infect. Dis.* **2020**, *6*, 916–921. https://doi.org/10.1021/acsinfecdis.9b00473.
145. Jalilian, A.; Hosseini, M.; Karimian, A.; Saddadi, F.; Sadeghi, M. Preparation and biodistribution of [^{201}Tl](III)vancomycin complex in normal rats. *Nukleonika* **2006**, *51*, 203–208.
146. Spoelstra, G.B.; Braams, L.M.; Ijpma, F.F.A.; van Oosten, M.; Feringa, B.L.; Szymanski, W.; Elsinga, P.H.; van Dijl, J.M. Bacteria-targeted imaging using vancomycin-based positron emission tomography tracers can distinguish infection from sterile inflammation. *Eur. J. Nucl. Med. Mol. Imaging* **2025**, *52*, 1878–1889. https://doi.org/10.1007/s00259-024-06997-z.
147. Spoelstra, G.B.; Blok, S.N.; Reali Nazario, L.; Noord, L.; Fu, Y.; Simeth, N.A.; IJpma, F.F.A.; van Oosten, M.; van Dijl, J.M.; Feringa, B.L.; et al. Synthesis and preclinical evaluation of novel ^{18}F-vancomycin-based tracers for the detection of bacterial infections using positron emission tomography. *Eur. J. Nucl. Med. Mol. Imaging* **2024**, *51*, 2583–2596. https://doi.org/10.1007/s00259-024-06717-7.
148. Jalilian, A.R.; Yousef, Y.K.; Rowshanfarzad, P.; Sabet, M.; Kamali-Dehghan, M.; Majdabadi, A. Preparation and preliminary evaluation of [^{55}Co](II) vancomycin. *Nucl. Sci. Tech.* **2008**, *19*, 347–353.
149. Kış, T.; Köse, Ş.; Yılmaz, O.; Kış, M.; Yurt, F.; Acar, E.; Bekiş, R.; Yılmaz, C.; Barış, M.; Diniz, G.; et al. Evaluation of ^{99m}Technetium-Vancomycin Imaging Potential in Experimental Rat Model for the Diagnosis of Infective Endocarditis. *Curr. Med. Imaging Rev.* **2021**, *17*, 781–789. https://doi.org/10.2174/1573405616666201229161850.
150. Rasheed, R.; Naqvi, S.A.R.; Gillani, S.J.H.; Zahoor, A.F.; Jielani, A.; Saeed, N. ^{99m}Tc-tazobactam, a novel infection imaging agent: Radiosynthesis, quality control, biodistribution, and infection imaging studies. *J. Label. Compd. Radiopharm.* **2017**, *60*, 242–249. https://doi.org/10.1002/jlcr.3494.

151. Singh, A.K.; Verma, J.; Bhatnager, A.; Sen, S. Tc-99m Isoniazid: A specific agent for diagnosis of tuberculosis. *World J. Nucl. Med.* **2003**, *2*, 292–305.
152. Weinstein, E.A.; Liu, L.; Ordonez, A.A.; Wang, H.; Hooker, J.M.; Tonge, P.J.; Jain, S.K. Noninvasive determination of 2-[^{18}F]-fluoroisonicotinic acid hydrazide pharmacokinetics by positron emission tomography in Mycobacterium tuberculosis-infected mice. *Antimicrob. Agents Chemother.* **2012**, *56*, 6284–6290. https://doi.org/10.1128/aac.01644-12.
153. Samad, A.; Sultana, Y.; Khar, R.K.; Aqil, M.; Kalam, M.A.; Chuttani, K.; Mishra, A.K. Radiolabeling and evaluation of alginate blend-isoniazid microspheres by 99mTc for the treatment of tuberculosis in rabbit model. *J. Drug Target.* **2008**, *16*, 509–515. https://doi.org/10.1080/10611860802201076.
154. DeMarco, V.P.; Ordonez, A.A.; Klunk, M.; Prideaux, B.; Wang, H.; Zhuo, Z.; Tonge, P.J.; Dannals, R.F.; Holt, D.P.; Lee, C.K.K.; et al. Determination of [^{11}C]Rifampin Pharmacokinetics within Mycobacterium tuberculosis-Infected Mice by Using Dynamic Positron Emission Tomography Bioimaging. *Antimicrob. Agents Chemother.* **2015**, *59*, 5768–5774, doi:doi:10.1128/aac.01146-15.
155. Shah, S.Q.; Khan, A.U.; Khan, M.R. Radiosynthesis and biodistribution of ^{99m}Tc-rifampicin: A novel radiotracer for in-vivo infection imaging. *Appl. Radiat. Isot.* **2010**, *68*, 2255–2260. https://doi.org/10.1016/j.apradiso.2010.05.014.
156. Shah, S.Q.; Alam, M. Synthesis of ^{99m}Tc-Rifabutin: A Potential Tuberculosis Radiodiagnostic Agent. *Infect. Disord. Drug Targets* **2017**, *17*, 185–191. https://doi.org/10.2174/1871526517666170606114650.
157. Syed, Q.S.; Saima, M. Synthesis of Labeled Rifabutin Dithiocarbamate: A Potential Mycobacterium Tuberculosis Imaging Agent. *J. Glycom. Metab.* **2017**, *1*, 12–23. https://doi.org/10.14302/issn.2572-5424.jgm-16-1352.
158. Ordonez, A.A.; Carroll, L.S.; Abhishek, S.; Mota, F.; Ruiz-Bedoya, C.A.; Klunk, M.H.; Singh, A.K.; Freundlich, J.S.; Mease, R.C.; Jain, S.K. Radiosynthesis and PET Bioimaging of ^{76}Br-Bedaquiline in a Murine Model of Tuberculosis. *ACS Infect. Dis.* **2019**, *5*, 1996–2002. https://doi.org/10.1021/acsinfecdis.9b00207.
159. Zhang, Z.; Ordonez, A.A.; Smith-Jones, P.; Wang, H.; Gogarty, K.R.; Daryaee, F.; Bambarger, L.E.; Chang, Y.S.; Jain, S.K.; Tonge, P.J. The biodistribution of 5-[^{18}F]fluoropyrazinamide in Mycobacterium tuberculosis-infected mice determined by positron emission tomography. *PLoS ONE* **2017**, *12*, e0170871. https://doi.org/10.1371/journal.pone.0170871.
160. Shah, S.Q.; Ullah, N. Preclinical Evaluation of ^{99m}Tc-Ethambutol, an Alternative Tuberculosis Diagnostic Tool. *Radiochemistry* **2019**, *61*, 233–237. https://doi.org/10.1134/S1066362219020176.
161. Buursma, A.R.; de Vries, E.F.; Garssen, J.; Kegler, D.; van Waarde, A.; Schirm, J.; Hospers, G.A.; Mulder, N.H.; Vaalburg, W.; Klein, H.C. [^{18}F]FHPG positron emission tomography for detection of herpes simplex virus (HSV) in experimental HSV encephalitis. *J. Virol.* **2005**, *79*, 7721–7727. https://doi.org/10.1128/jvi.79.12.7721-7727.2005.
162. Vries, E.F.d.; van Waarde, A.; Harmsen, M.C.; Mulder, N.H.; Vaalburg, W.; Hospers, G.A. [^{11}C]FMAU and [^{18}F]FHPG as PET tracers for herpes simplex virus thymidine kinase enzyme activity and human cytomegalovirus infections. *Nucl. Med. Biol.* **2000**, *27*, 113–119. https://doi.org/10.1016/s0969-8051(99)00105-5.
163. Muñoz-Álvarez, K.A.; Altomonte, J.; Laitinen, I.; Ziegler, S.; Steiger, K.; Esposito, I.; Schmid, R.M.; Ebert, O. PET imaging of oncolytic VSV expressing the mutant HSV-1 thymidine kinase transgene in a preclinical HCC rat model. *Mol. Ther.* **2015**, *23*, 728–736. https://doi.org/10.1038/mt.2015.12.
164. Gambhir, S.S.; Bauer, E.; Black, M.E.; Liang, Q.; Kokoris, M.S.; Barrio, J.R.; Iyer, M.; Namavari, M.; Phelps, M.E.; Herschman, H.R. A mutant herpes simplex virus type 1 thymidine kinase reporter gene shows improved sensitivity for imaging reporter gene expression with positron emission tomography. *Proc. Natl. Acad. Sci. USA* **2000**, *97*, 2785–2790. https://doi.org/10.1073/pnas.97.6.2785.
165. Tisseraud, M.; Goutal, S.; Bonasera, T.; Goislard, M.; Desjardins, D.; Le Grand, R.; Parry, C.M.; Tournier, N.; Kuhnast, B.; Caillé, F. Isotopic Radiolabeling of the Antiretroviral Drug [^{18}F]Dolutegravir for Pharmacokinetic PET Imaging. *Pharmaceuticals* **2022**, *15*, 587. https://doi.org/10.3390/ph15050587.
166. Di Mascio, M.; Srinivasula, S.; Bhattacharjee, A.; Cheng, L.; Martiniova, L.; Herscovitch, P.; Lertora, J.; Kiesewetter, D. Antiretroviral tissue kinetics: *In vivo* imaging using positron emission tomography. *Antimicrob. Agents Chemother.* **2009**, *53*, 4086–4095. https://doi.org/10.1128/aac.00419-09.
167. Seki, C.; Oh-Nishi, A.; Nagai, Y.; Minamimoto, T.; Obayashi, S.; Higuchi, M.; Takei, M.; Furutsuka, K.; Ito, T.; Zhang, M.R.; et al. Evaluation of [^{11}C]oseltamivir uptake into the brain during immune activation by systemic polyinosine-polycytidylic acid injection: A quantitative PET study using juvenile monkey models of viral infection. *EJNMMI Res.* **2014**, *4*, 24. https://doi.org/10.1186/s13550-014-0024-8.
168. Lupetti, A.; Welling, M.M.; Mazzi, U.; Nibbering, P.H.; Pauwels, E.K. Technetium-99m labelled fluconazole and antimicrobial peptides for imaging of Candida albicans and Aspergillus fumigatus infections. *Eur. J. Nucl. Med. Mol. Imaging* **2002**, *29*, 674–679. https://doi.org/10.1007/s00259-001-0760-7.

169. de Assis, D.N.; Araújo, R.S.; Fuscaldi, L.L.; Fernandes, S.O.A.; Mosqueira, V.C.F.; Cardoso, V.N. Biodistribution of free and encapsulated ^{99m}Tc-fluconazole in an infection model induced by Candida albicans. *Biomed. Pharmacother.* **2018**, *99*, 438–444. https://doi.org/10.1016/j.biopha.2018.01.021.
170. Fischman, A.J.; Alpert, N.M.; Livni, E.; Ray, S.; Sinclair, I.; Elmaleh, D.R.; Weiss, S.; Correia, J.A.; Webb, D.; Liss, R. Pharmacokinetics of ^{18}F-labeled fluconazole in rabbits with candidal infections studied with positron emission tomography. *J. Pharmacol. Exp. Ther.* **1991**, *259*, 1351–1359. https://doi.org/10.1016/S0022-3565(25)20553-5.
171. Reyes, A.L.; Fernández, L.; Rey, A.; Terán, M. Development and evaluation of ^{99m}Tc-tricarbonyl-caspofungin as potential diagnostic agent of fungal infections. *Curr. Radiopharm.* **2014**, *7*, 144–150. https://doi.org/10.2174/1874471007666141015121838.
172. El-Kawy, O.A.; Sayed, M.S.; Abdel-Razek, A.S. Preparation and evaluation of ^{99m}Tc-anidulafungin: A potential radiotracer for fungal infection. *J. Radioanal. Nucl. Chem.* **2020**, *325*, 683–694. https://doi.org/10.1007/s10967-020-07274-7.
173. Page, L.; Ullmann Andrew, J.; Schadt, F.; Wurster, S.; Samnick, S. *In vitro* Evaluation of Radiolabeled Amphotericin B for Molecular Imaging of Mold Infections. *Antimicrob. Agents Chemother.* **2020**, *64*, e02377-19. https://doi.org/10.1128/aac.02377-19.
174. Fernández, L.; Terán, M. Development and Evaluation of ^{99m}Tc-Amphotericin Complexes as Potential Diagnostic Agents in Nuclear Medicine. *Int. J. Infect.* **2017**, *4*, e62150.
175. Schottelius, M.; Simecek, J.; Hoffmann, F.; Willibald, M.; Schwaiger, M.; Wester, H.J. Twins in spirit—Episode I: Comparative preclinical evaluation of [^{68}Ga]DOTATATE and [^{68}Ga]HA-DOTATATE. *EJNMMI Res.* **2015**, *5*, 22. https://doi.org/10.1186/s13550-015-0099-x.
176. Ruiz-Bedoya, C.A.; Mota, F.; Tucker, E.W.; Mahmud, F.J.; Reyes-Mantilla, M.I.; Erice, C.; Bahr, M.; Flavahan, K.; de Jesus, P.; Kim, J.; et al. High-dose rifampin improves bactericidal activity without increased intracerebral inflammation in animal models of tuberculous meningitis. *J. Clin. Investig.* **2022**, *132*, e155851. https://doi.org/10.1172/jci155851.
177. Yaghoubi, S.S.; Gambhir, S.S. PET imaging of herpes simplex virus type 1 thymidine kinase (HSV1-tk) or mutant HSV1-sr39tk reporter gene expression in mice and humans using [^{18}F]FHBG. *Nat. Protoc.* **2006**, *1*, 3069–3075. https://doi.org/10.1038/nprot.2006.459.
178. Gajdács, M. The Concept of an Ideal Antibiotic: Implications for Drug Design. *Molecules* **2019**, *24*, 892. https://doi.org/10.3390/molecules24050892.
179. Roberts, J.A.; Pea, F.; Lipman, J. The Clinical Relevance of Plasma Protein Binding Changes. *Clin. Pharmacokinet.* **2013**, *52*, 1–8. https://doi.org/10.1007/s40262-012-0018-5.
180. Yu, W.; MacKerell, A.D., Jr. Computer-Aided Drug Design Methods. *Methods Mol. Biol.* **2017**, *1520*, 85–106. https://doi.org/10.1007/978-1-4939-6634-9_5.
181. Finazzi, S.; Luci, G.; Olivieri, C.; Langer, M.; Mandelli, G.; Corona, A.; Viaggi, B.; Di Paolo, A. Tissue Penetration of Antimicrobials in Intensive Care Unit Patients: A Systematic Review-Part I. *Antibiotics* **2022**, *11*, 1164. https://doi.org/10.3390/antibiotics11091164.
182. Jager, N.G.L.; van Hest, R.M.; Lipman, J.; Roberts, J.A.; Cotta, M.O. Antibiotic exposure at the site of infection: Principles and assessment of tissue penetration. *Expert Rev. Clin. Pharmacol.* **2019**, *12*, 623–634. https://doi.org/10.1080/17512433.2019.1621161.
183. Shah, S.; Lai, J.; Basuli, F.; Martinez-Orengo, N.; Patel, R.; Turner, M.L.; Wang, B.; Shi, Z.D.; Sourabh, S.; Peiravi, M.; et al. Development and preclinical validation of 2-deoxy 2-[^{18}F]fluorocellobiose as an Aspergillus-specific PET tracer. *Sci. Transl. Med.* **2024**, *16*, eadl5934. https://doi.org/10.1126/scitranslmed.adl5934.
184. Sheng, J.; Zhang, T. Advancing drug development with "Fit-for-Purpose" modeling informed approaches. *J. Pharmacokinet. Pharmacodyn.* **2025**, *52*, 52. https://doi.org/10.1007/s10928-025-09995-2.
185. El-ghany, E.A.; Amin, A.M.; El-kawy, O.A. and Amin, M. Technetium-99m labeling and freeze-dried kit formulation of levofloxacin (L-Flox): a novel agent for detecting sites of infection. *J Label Compd Radiopharm*, **2007**, *50*, 25-31.
186. Bush, K.; Bradford, P.A. β-Lactams and β-Lactamase Inhibitors: An Overview. *Cold Spring Harb Perspect Med*. **2016**, *6*, a025247.
187. Bokhari, T.H.; Akbar, M.U.; HINA, S.; USMAN, M.; Haq, A.; Roohi, S.; Saeed, S. Direct Labelling of Medically Interesting 99mTc-benzyl Penicillin. *Oxidation Communications*, **2016**, *1*, 187.
188. Yurt Lambrecht, F.; Yilmaz, O.; Unak, P.; Seyitoglu, B.; Durkan, K.; Baskan, H. Evaluation of 99mTc-Cefuroxime axetil for imaging of inflammation. *Journal of Radioanalytical and Nuclear Chemistry*, **2008**, *277*,491-494.
189. Bosnar, M.; Kelnerić, Z.; Munić, V.; Eraković, V.; Parnham, M.J. Cellular Uptake and Efflux of Azithromycin, Erythromycin, Clarithromycin, Telithromycin, and Cethromycin. *Antimicrobial Agents and Chemotherapy*, **2005**, *49*, 2372-2377.
190. Gabler, W.L. Fluxes and accumulation of tetracyclines by human blood cells. Res *Commun Chem Pathol Pharmacol*, **1991**, *72*, 39-51.

191. Karpuz, M.; Atlihan-Gundogdu, E.; Demir, E.S.; Senyigit, Z. Radiolabeled Tedizolid Phosphate Liposomes for Topical Application: Design, Characterization, and Evaluation of Cellular Binding Capacity. *AAPS PharmSciTech*, **2021**, *22*, 62.
192. Samuel, G.; Kothari, K.; Banerjee, S.; Das, T.; Subramanian, S.; Kameshwaran, M.; Pillai, M.R.A.; Venkatesh, M. On the 99mTc-labeling of isoniazid with different 99mTc cores. *J Label Compd Radiopharm*, **2005**, *48*, 363-377.
193. Soghomonyan, S.; Hajitou, A.; Rangel, R.; Trepel, M.; Pasqualini, R.; Arap, W.; Gelovani, J.G.; Alauddin, M.M. Molecular PET imaging of HSV1-tk reporter gene expression using [^{18}F]FEAU. *Nat Protoc*, **2007**, *2*, 416-23.
194. Hackman, T.; Doubrovin, M.; Balatoni, J.; Beresten, T.; Ponomarev, V.; Beattie, B.; Finn, R.; Bornmann, W.; Blasberg, R.; Gelovani, J.G. Imaging expression of cytosine deaminase-herpes virus thymidine kinase fusion gene (CD/TK) expression with [124I]FIAU and PET. *Mol Imaging*, **2002**, *1*, 36-42.
195. Alauddin, M.M. Positron emission tomography (PET) imaging with (18)F-based radiotracers. *Am J Nucl Med Mol Imaging*, **2012**, *2*, 55-76.
196. Lau, C.-Y.; Martinez-Orengo, N.; Lyndaker, A.; Flavahan, K.; Johnson, R.F.; Shah, S.; Hammoud, D.A. Advances and Challenges in Molecular Imaging of Viral Infections. *J Infect Dis.*, **2023**, *228*, S270-S280.
197. Schäfer-Korting, M.; Korting, H.C.; Rittler, W.; Obermüller, W.; Influence of serum protein binding on the in vitro activity of anti-fungal agents. *Infection*, **1995**, *23*, 292-297.

Disclaimer/Publisher's Note: The statements, opinions and data contained in all publications are solely those of the individual author(s) and contributor(s) and not of MDPI and/or the editor(s). MDPI and/or the editor(s) disclaim responsibility for any injury to people or property resulting from any ideas, methods, instructions or products referred to in the content.
